# Determining the OPTIMAL DTI analysis method for application in cerebral small vessel disease

**DOI:** 10.1016/j.nicl.2022.103114

**Published:** 2022-07-13

**Authors:** Marco Egle, Saima Hilal, Anil M Tuladhar, Lukas Pirpamer, Steven Bell, Edith Hofer, Marco Duering, James Wason, Robin G Morris, Martin Dichgans, Reinhold Schmidt, Daniel J Tozer, Thomas R. Barrick, Christopher Chen, Frank-Erik de Leeuw, Hugh S Markus

**Affiliations:** a Stroke Research Group, Department of Clinical Neurosciences, University of Cambridge, Cambridge, United Kingdom; b Department of Pharmacology, National University of Singapore, Singapore; c Memory Ageing and Cognition Center, National University Health System, Singapore; d Department of Neurology, Donders Center for Medical Neuroscience, Radboud University Medical Center, Nijmegen, the Netherlands; e Department of Neurology, Medical University of Graz, Graz, Austria; f Institute for Medical Informatics, Statistics and Documentation, Medical University of Graz, Graz, Austria; g Institute for Stroke and Dementia Research, University Hospital, LMU Munich, Munich, Germany; h Medical Image Analysis Center (MIAC) and Department of Biomedical Engineering, University of Basel, Basel, Switzerland; i Population Health Sciences Institute, Newcastle University, Baddiley-Clark Building, Newcastle Upon Tyne, United Kingdom; j Department of Psychology (R.G.M.), King's College, Institute of Psychiatry, Psychology and Neuroscience, London, United Kingdom; k Munich Cluster for Systems Neurology (SyNergy), Munich, Germany; l German Center for Neurodegenerative Diseases (DZNE), Munich, Germany; m Neurosciences Research Centre, Institute for Molecular and Clinical Sciences, St George’s, University of London, United Kingdom

**Keywords:** _AxD_, _Axial diffusivity_, _AD_, _Alzheimer, s disease_, _Adj. R2_, _Model’s explained variance adjusted by the number of predictors_, _AIC_, _Akaike information criterion_, _ANCOVA_, _Analysis of covariance_, _ASPS-Fam_, _Austrian Stroke Prevention Study_, _AUC_, _Area under the curve_, _CSF_, _Cerebral spinal fluid_, _DSEG θ_, _Diffusion tensor image segmentation θ_, _DSM_, _Diagnostic and Statistical Manual of Mental Disorders_, _DTI_, _Diffusion tensor imaging_, _FA_, _Fractional anisotropy_, _FACT_, _Fiber assignment by continuous tracking_, _Geff_, _Global efficiency network measure_, _HR_, _Hazard ratio_, _MCI_, _Mild cognitive impairment_, _MD_, _Mean diffusivity_, _MD median_, _The median of mean diffusivity histogram measure_, _OPTIMAL_, _OPtimising mulTImodal MRI markers for use as surrogate markers in trails of Vascular Cognitive Impairment due to cerebrAl small vesseL disease_, _OR_, _Odds ratio_, _PC1_, _Scores of the first principal component_, _PCA_, _Principal component analysis_, _PH_, _Normalised peak height of the histogram_, _pkval_, _peak value of the histogram_, _PSMD_, _Peak width skeletonized mean diffusivity_, _RD_, _Radial diffusivity_, _ROC_, _Receiver operating curve_, _RUN DMC_, _Radboud University Nijmegen Diffusion tensor and Magnetic resonance imaging Cohort_, _SCANS_, _St George’s Cognition and Neuroimaging in Stroke_, _SVD_, _Cerebral small vessel disease_, _TBSS_, _Tract-based spatial statistics_, _TPM_, _Tissue probability maps_, _VD_, _Vascular dementia_, _WM_, _White matter_, _WMH_, _White matter hyperintensity_, Small vessel disease, Diffusion tensor imaging, Dementia, Surrogate marker, Cognition

## Abstract

**Background:**

DTI is sensitive to white matter (WM) microstructural damage and has been suggested as a surrogate marker for phase 2 clinical trials in cerebral small vessel disease (SVD). The study’s objective is to establish the best way to analyse the diffusion-weighted imaging data in SVD for this purpose. The ideal method would be sensitive to change and predict dementia conversion, but also straightforward to implement and ideally automated. As part of the OPTIMAL collaboration, we evaluated five different DTI analysis strategies across six different cohorts with differing SVD severity.

**Methods:**

Those 5 strategies were: (1) conventional mean diffusivity WM histogram measure (MD median), (2) a principal component-derived measure based on conventional WM histogram measures (PC1), (3) peak width skeletonized mean diffusivity (PSMD), (4) diffusion tensor image segmentation θ (DSEG θ) and (5) a WM measure of global network efficiency (Geff). The association between each measure and cognitive function was tested using a linear regression model adjusted by clinical markers. Changes in the imaging measures over time were determined. In three cohort studies, repeated imaging data together with data on incident dementia were available. The association between the baseline measure, change measure and incident dementia conversion was examined using Cox proportional-hazard regression or logistic regression models. Sample size estimates for a hypothetical clinical trial were furthermore computed for each DTI analysis strategy.

**Results:**

There was a consistent cross-sectional association between the imaging measures and impaired cognitive function across all cohorts. All baseline measures predicted dementia conversion in severe SVD. In mild SVD, PC1, PSMD and Geff predicted dementia conversion. In MCI, all markers except Geff predicted dementia conversion. Baseline DTI was significantly different in patients converting to vascular dementia than to Alzheimer’ s disease. Significant change in all measures was associated with dementia conversion in severe but not in mild SVD. The automatic and semi-automatic measures PSMD and DSEG θ required the lowest minimum sample sizes for a hypothetical clinical trial in single-centre sporadic SVD cohorts.

**Conclusion:**

DTI parameters obtained from all analysis methods predicted dementia, and there was no clear winner amongst the different analysis strategies. The fully automated analysis provided by PSMD offers advantages particularly for large datasets.

## Highlights

• We were not able to identify a single optimal diffusion-weighted imaging analysis strategy across all 6 cohorts.
• Diffusion tensor imaging measures at baseline predicted dementia conversion in cerebral small vessel disease and mild cognitive impairment.
• Diffusion tensor imaging measures at baseline may be sensitive to differentiate between later vascular dementia vs Alzheimer’s disease dementia.
• Diffusion tensor imaging measures significantly changed over time in cohorts with cerebral small vessel disease and cohorts with mild cognitive impairment. Change in diffusion tensor imaging measures were only consistently associated with dementia conversion in severe SVD.
• The diffusion tensor imaging measures PSMD and DSEG required the lowest minimum sample sizes for a hypothetical clinical trial in patients with sporadic cerebral small vessel disease and mild cognitive impairment.

## 1 Introduction

Cerebral small vessel disease (SVD) is a highly prevalent condition which causes lacunar stroke, vascular cognitive impairment (VCI) and dementia (Wardlaw et al., 2019). Characteristic appearances seen on magnetic resonance imaging (MRI) include white matter hyperintensities (WMH), lacunes, cerebral microbleeds, and enlarged perivascular spaces (Pantoni, 2010). Cognitive impairment is common, with early impairment of executive function and information processing speed (Lawrence et al., 2013). SVD is the most common vascular pathology underlying VCI, although only a small proportion of patients with radiological features of SVD will progress to dementia within a few years (Amin Al Olama et al., 2020, Debette et al., 2019, Debette and Markus, 2010, Lawrence et al., 2017, Prins et al., 2004, Wu et al., 2019).

Despite the enormous global burden of SVD in causing stroke, dementia and, disability, there are few effective treatments for the disease (Smith and Markus, 2020). One challenge is that large sample sizes are required to examine new therapies using clinical outcomes such as recurrent stroke, cognitive decline and dementia (Amin Al Olama et al., 2020, Baykara et al., 2016, Benjamin et al., 2016, Lawrence et al., 2015, Nam et al., 2017). Moreover, cognitive testing has been shown to be relatively insensitive as an endpoint measure in phase 2 trials. Hence, there is increasing interest in the use of other markers including MRI as a surrogate endpoint to allow evaluation of therapies with smaller sample sizes in phase 2 trials, prior to scaling to primary clinical endpoints in larger phase 3 trials (Baykara et al., 2016, Benjamin et al., 2016, Lawrence et al., 2015, Schmidt et al., 2004).

Diffusion tensor imaging (DTI) has been proposed as a promising surrogate marker in SVD trials (Baykara et al., 2016, van den Brink et al., 2022, Zeestraten et al., 2017, 2016). DTI measures have been shown to be sensitive to white matter (WM) damage in SVD both within WMH and in apparently ‘normal appearing’ WM (O’Sullivan et al., 2004, Pasi et al., 2016). In cross-sectional studies DTI measures correlated with cognitive impairment more strongly than WMH (Croall et al., 2017, Lawrence et al., 2013, Tuladhar et al., 2015). Recent longitudinal studies have also demonstrated that both baseline DTI, and change in DTI, predict future dementia risk (Egle et al., 2022, Power et al., 2019, van Uden et al., 2016, Zeestraten et al., 2017). Changes in DTI markers can be detected over periods as little as 1–3 years, which are time durations often used in phase 2 clinical trials (Markus et al., 2021, Nitkunan et al., 2008, van Leijsen et al., 2019, Williams et al., 2019, Williams et al., 2017, Zeestraten et al., 2016).

However, DTI analysis can be time consuming, and automated or semi-automated analysis techniques that are robust across different sites and populations are attractive, particularly for clinical trials. Such methods include Peak width of Skeletonized Mean diffusivity (PSMD), a DTI-derived measure based on skeletonization and histogram analysis, (Baykara et al., 2016) and Diffusion tensor image SEGmentation θ (DSEG θ) a diffusion tensor image (DTI) segmentation technique to describe SVD related changes in a single unitary score across the whole cerebrum ((Baykara et al., 2016, Williams et al., 2017). Previous analyses have tended to focus on one DTI histogram measure such as MD median or MD peak height. However, it is possible that analyzing multiple measures improves prediction. The reason for this is that different DTI parameters represent slightly different aspects of pathology; for example radial diffusivity (RD) has been suggested as a marker of myelin loss and axial diffusivity (AxD) as a marker of demyelination (Song et al., 2002, Winklewski et al., 2018). Techniques such as principal component analysis which summarize different DTI measures may therefore show better prediction than a single histogram measure. Lastly advanced MRI diffusion data and tractography can be used to reconstruct WM tracts and networks, and measures of the integrity of such brain networks have been shown to correlate strongly with impaired cognitive function, and predict future dementia risk (Lawrence et al., 2018, Lawrence et al., 2014, Tuladhar et al., 2016b).

The overall aim of the OPTIMAL (OPtimising mulTImodal MRI markers for use as surrogate markers in trials of Vascular Cognitive Impairment due to cerebrAl small vesseL disease) collaboration is to find the most efficient way of using MRI measures in clinical trials. A previous OPTIMAL study demonstrated that conventional WM DTI measures such as MD median predicted impaired cognitive function and dementia conversion in SVD and MCI patient cohorts (Egle et al., 2022). In this OPTIMAL study, we compared the performance of different strategies of quantifying diffusion-weighted imaging, including MD median, in predicting conversion to dementia and impaired cognitive function. We then used the data to determine sample sizes for a potential intervention study using the different DTI approaches as endpoints. WM ultrastructural damage, characterised by DTI, has been associated with cognitive not only in patients with symptomatic SVD, but also in elderly community populations, and in cohorts with MCI; in all groups WM damage due to SVD has been postulated to play a role in cognitive impairment. For this reason, we assessed the performance of makers across a range of cohorts including both severe and mild SVD, healthy elderly community subjects, and subjects with MCI. For our analyses, we used all-cause dementia as the primary outcome measure. This is because is the most relevant outcome measures for a clinical trial, because accurate diagnosis of subtypes in life may be inaccurate and related to the later point because population-based studies have shown at post-mortem that most cases of dementia have a mixed pathology often including vascular changes of SVD (Schneider et al., 2007, Toledo et al., 2013).

## 2 Materials and methods

### 2.1 Cohorts studied

Five prospective longitudinal cohort studies were included (Table 1) which had differing severity of SVD, ranging from severe symptomatic disease presenting with lacunar stroke and confluent white matter hyperintensities, to a community population in whom “asymptomatic” SVD was detected on MRI. In addition, we included a cohort with mild cognitive impairment (MCI). They were:

i. St George’s Cognition and Neuroimaging in Stroke (SCANS) included 121 individuals with severe symptomatic SVD defined as a symptomatic lacunar infarct with confluent WMH (Fazekas grade >=2) (Fazekas et al., 1987) (Zeestraten et al., 2016)
ii. Radboud University Nijmegen Diffusion tensor and Magnetic Resonance Imaging Cohort (RUN DMC) study included 503 predominantly mild symptomatic SVD patients defined as the presence of lacunes and or any WMH on neuroimaging and accompanying stroke, subacute cognitive or motor symptoms (van Norden et al., 2011)
iii. PRESERVE multicenter clinical trial with imaging included 111 patients with severe symptomatic SVD defined as a symptomatic lacunar infarct with confluent WMH (Fazekas grade >=2) (Fazekas et al., 1987) (Croall et al., 2017)
iv. HARMONISATION study included 127 patients with mild cognitive impairment (MCI) impaired in at least one cognitive domain of a formal neuropsychological test battery, with or without a history of stroke (Hilal et al., 2017)
v. Austrian Stroke Prevention Study (ASPS-Fam), a community population of 382 individuals free of dementia and stroke as well as demonstrating normal neurological function in whom SVD was severity was assessed on MRI (Seiler et al., 2014)
vi. CADASIL cohort of 58 patients with monogenic SVD confirmed by genetic testing or skin biopsy (Baykara et al., 2016).

**Table 1:** Overview about the cohort studies included in the OPTIMAL project. The table shows the number of patients enrolled, the respective inclusion criteria and the type of dementia diagnosis given in each study.

**Cohort**	**No of patients**	**Country**	**Duration**	**Inclusion criteria**	**Dementia diagnosis**	**Vascular Dementia**	**Alzheimer’s Disease**
SCANS (Zeestraten et al., 2016) (Severe SVD)	121	United Kingdom	3 years imaging measures 5 years clinical measures	Symptomatic SVD, defined as a clinical lacunar stroke syndrome with MRI evidence of an anatomically corresponding lacunar infarct, and with confluent regions of WMH graded ≥ 2 on the modified Fazekas scale (Fazekas et al., 1987)	Diagnostic and Statistical Manual of Mental Disorders V	–	–
RUN DMC (van Norden et al., 2011) (Mild SVD)	503	The Netherlands	9 years	SVD, defined as the presence of lacunes and or WMH on neuroimaging and accompanying acute (lacunar) or subacute (cognitive, motor) symptoms	Diagnostic and Statistical Manual of Mental Disorders IV	National Institute of Neurological Disorders and Stroke– Association Internationale pour la Recherché et l’Enseignement en Neurosciencescriteria (Erkinjuntti, 1994)	National Institute on Aging and Alzheimer's Association criteria (McKhann et al., 2011)
PRESERVE (Croall et al., 2017) (Severe SVD)	111	United Kingdom	2 years	Symptomatic SVD, defined as a clinical lacunar stroke syndrome with MRI evidence of an anatomically corresponding lacunar infarct, and with confluent regions of WMH graded ≥ 2 on the modified Fazekas scale (Fazekas et al., 1987)	–	–	–
HARMONISATION (Hilal et al., 2017) (Mild cognitive impairment)	127	Singapore	2 years	Subgroup of patients with mild cognitive impairment (MCI) impaired in at least one cognitive domain of a formal neuropsychological test battery, with or without a history of stroke	Diagnostic and Statistical Manual of Mental Disorders IV	National Institute of Neurological Disorders and Stroke– Association Internationale pour la Recherché et l’Enseignement en Neurosciencescriteria (Erkinjuntti, 1994)	National Institute on Aging and Alzheimer's Association criteria (McKhann et al., 2011)
ASPS-Fam (Seiler et al., 2014) (Community population)	382	Austria	Only baseline included	Being free of dementia and stroke as well as demonstrating normal neurological function	–	–	–

CADASIL (Baykara et al., 2016) (Monogenic SVD)	58	Germany	1.5 years	Diagnosis of CADASIL confirmed by genetic testing or skin biopsy	–	–	–

SVD = small vessel disease, WMH = white matter hyperintensities, MCI = mild cognitive impairment.

**Table 2:** **Overview over the cohorts.** Clinical markers, imaging markers and sample sizes both at baseline and longitudinal are shown.

	**SCANS**	**RUN DMC**	**HARMONISATION**	**PRESERVE**	**ASPS-Fam**	**CADASIL**
*Demographics*	*Mean (SD)*	*Mean (SD)*	*Mean (SD)*	*Mean (SD)*	*Mean (SD)*	*Mean (SD)*
Age (SD)	70.01 (9.75)	65.62 (8.81)	72.23 (8.47)	68.07 (9.11)	65.43 (10.67)	47.90 (9.77)
Sex, male (%)	78 (0.65)	284 (0.57)	57 (0.45)	43 (0.39)	139 (0.40)	26 (0.45)
Included in cross-sectional analysis	yes	yes	yes	yes	yes	yes
Sample size with complete DTI measures at baseline	113	435	127	101	256	54
Dementia cases with complete baseline imaging	18	50	23	–	–	–
Included in longitudinal analysis	yes	yes	yes	yes	no	yes
Sample size in longitudinal analysis with complete repeated DTI measures	97	267	127	81	–	53
Dementia cases with complete repeated imaging	17	12	23	–	–	–
*Baseline complete DTI parameters*	*Mean (SD)*	*Mean (SD)*	*Mean (SD)*	*Mean (SD)*	*Mean (SD)*	*Mean (SD)*
MD Median (mm^2^/s)	8.01e-04 (4.09e-05)	8.28e-04 (3.57e-05)	8.82e-04 (6.08e-05)	7.87e-04 (4.28e-05)	7.69e-04 (3.04e-05)	8.89e-04 (1.30e-04)
PSMD (mm^2^/s)	3.80e-04 (1.14e-04)	3.45e-04 (7.39e-05)	3.60e-04 (7.71e-05)	3.93e-04 (9.77e-05)	2.97e-04 (5.31e-05)	5.63e-04 (1.88e-04)
DSEG θ (mm^2^/s)	22.04 (9.62)	21.01 (9.41)	32.31 (8.24)	47.65 (4.01)	49.95 (8.13)	22.39 (12.36)
Geff	7.94 (2.32)	3.90e-03 (8.41e-04)	0.41 (0.22)	0.17 (0.10)	4.36 (1.65)	2.16 (1.16)

DTI = Diffusion tensor imaging, MD Median = Mean diffusivity Median of the WM histogram, PC1 = Scores of the first principal component, PSMD = Peak width of skeletonized mean diffusivity, DSEG θ = Diffusion tensor image segmentation θ, Geff = Global efficiency network measure.

Full details of the cohorts are given in Table 1.

### 2.2 Standard protocol approvals, registrations, and patient consents

Local ethical approval was obtained for each cohort study and each participant gave written informed consent according to the Declaration of Helsinki.

### 2.3 Magnetic resonance imaging acquisitions and clinical measures

Fluid-attenuated inversion recovery (FLAIR), T1-weighted and diffusion-weighted data were acquired in all cohorts. The imaging and clinical details of all cohorts have been fully described previously and are briefly outlined and referenced below.

#### 2.3.1 SCANS

MRI scanning took place at baseline and over 3 yearly follow-up sessions using a 1.5-T General Electric Signa HDxt MRI system (Zeestraten et al., 2016). Imaging acquisition and parameters are described in **eTable 1**. Age-standardized cognitive test scores sensitive in detecting patterns of cognitive impairment in SVD were used to form an index measure of Global Cognition (**eTable 2**) (Lawrence et al., 2015). Mini Mental State Examination (MMSE) test scores were additionally obtained (Folstein et al., 1975). Dementia was diagnosed employing the Diagnostic and Statistical Manual of Mental Disorders V and assessed up to 5 years (Table 1) (Zeestraten et al., 2017).

#### 2.3.2 RUN DMC

MRI scanning was performed at baseline and 2 follow-up time points (5 years, 9 years). MRI acquisition was based on 1.5-T Siemens Magnetom Avanto MRI machine (**eTable 1**) (van Norden et al., 2011). Age-standardised scores were used to compute a Global Cognition index score, (**eTable 2**) (van Uden et al., 2015). MMSE test scores were also obtained (Folstein et al., 1975). Dementia diagnosis was based on DSM-IV criteria as described previously and assessed up to 9 years (Table 1) (van Uden et al., 2016). Alzheimer’s disease and vascular dementia diagnosis was based on National Institute on Aging–Alzheimer’s Association criteria (NIA-AA) (McKhann et al., 2011) and the National Institute of Neurological Disorders and Stroke–Association Internationale pour la Recherché et l’Enseignement en Neurosciences criteria (NINDS-AIREN) (Erkinjuntti, 1994).

#### 2.3.3 Preserve

MRI acquisition took place on eight 3 T MRI scanners (3 Philips Achieva TX, 1 Philips Achieva, 1 Philips Ingenia, 1 Siemens Verio, 1 Siemens Prisma, 1 Siemens Magnetom Prisma fit) at baseline and 2 years (**eTable 3**). (Croall et al., 2017) Neuropsychological test scores related to executive function, processing speed and memory were age-normalised and used to create a Global Cognition index score (Markus et al., 2021) (**eTable 2**). Montreal Cognitive Assessment (MOCA) test scores were also employed in this study (Nasreddine et al., 2005). No dementia diagnoses were made in this cohort.

#### 2.3.4 Harmonisation

Imaging data were acquired on a 3 T Siemens Magnetom Trio Tim system (**eTable 1**) (Hilal et al., 2017). Baseline and 2 years follow-up DTI data was available. Cognitive test scores were standardised to the mean and standard deviation derived from all patients without dementia enrolled in HARMONISATION to form a measure of Global Cognition (**eTable 2**). Only cognitive measures of patients with MCI were part of this study. MOCA test scores were additionally included in the study (Nasreddine et al., 2005). Dementia diagnosis was based on DSM-IV criteria and assessed up to 2 years (Table 1). Dementia diagnosis subtyping was also based on the NIA-AA (McKhann et al., 2011) and NINDS-AIREN criteria (Erkinjuntti, 1994).

#### 2.3.5 ASPS-Fam

Magnetic resonance acquisition was performed on a 3 T Tim Trio whole body scanner (**eTable 1**). Only DTI measures at baseline were analysed due to a low sample size on the follow-up timepoint. Dementia conversion as a clinical outcome variable was therefore not included in the analysis. Global Cognition was created based on age-standardised test scores using a principal component analysis (**eTable 2**) (Davies et al., 2018).

#### 2.3.6 CADASIL cohort

Imaging data were based on a 1.5-T GE Signa system in Munich (Baykara et al., 2016). Further details regarding acquisition parameters are found in **eTable 1**. To measure cognitive function, the Trail-making test–B was used (**eTable 2**). MMSE test scores were additionally obtained from the patients (Folstein et al., 1975). The main outcome score was normalized for age and education (Tombaugh, 2004). Dementia conversion as a variable was not included.

## 3 Magnetic resonance imaging analysis

### 3.1 Pre-processing of diffusion raw data

In SCANS, PRESERVE, HARMONISATION, ASPS-Fam and CADASIL a standardized DTI analysis protocol was employed at the central site in Cambridge (SCANS. PRESERVE, HARMONISATION, CADASIL), or the co-ordinating site was guided using an identical protocol (ASPS-Fam). Diffusion-weighted image pre-processing was carried out with the eddy correct software from ‘FDT’, FMRIB’s Diffusion Toolbox, (https://fsl.fmrib.ox.ac.uk/fsl/fslwiki/FDT) (Andersson and Sotiropoulos, 2016). In RUN DMC the diffusion-weighted images were pre-processed prior to OPTIMAL with in-house developed iteratively reweighted least squares algorithm at the Radboud University Medical Center in Nijmegen (Zwiers, 2010).

### 3.2 Five different strategies of quantifying DTI data

Five different strategies of quantifying DTI parameters were compared in each cohort:

1. Conventional WM histogram measure MD median (MD median)
2. The principal component measure (PC1)
3. Peak width skeletonized mean diffusivity (PSMD)
4. Diffusion tensor image segmentation θ (DSEG θ)
5. Global structural network efficiency measure (Geff)

All other conventional WM histogram measures included in the analysis were used to compute the PC1 measure.

#### 3.2.1 Conventional WM histogram measures including MD median

MD median and 19 other WM histogram measures requiring manual interventions were computed on the mean diffusivity (MD), fractional anisotropy (FA), axial diffusivity (AxD) and radial diffusivity (RD) DTI maps. The 5 histogram measures for each of the maps were: Median, normalised peak height (PH), peak value, skew, kurtosis. Hence, 20 histogram measures were computed for each patient.

Using SPM12b (Statistical parametric mapping), T1-weighted scans were segmented into grey matter, WM and cerebral spinal fluid tissue probability maps (TPM) (Ashburner and Friston, 2005). MD, FA, AxD and RD maps were generated with ‘DTIFIT’, ﻿part of the Functional Magnetic Resonance Imaging of the Brain (FMRIB) Software Library (FSL) (Behrens et al., 2007, 2003). Employing FMRIB Linear Image Registration Tool (FLIRT) (Jenkinson and Smith, 2001), FLAIR to T1-weighted and T1-weighted to b0 registrations (the average of all the b = 0 s mm^−2^ images in the DTI sequence) were employed (Jenkinson and Smith, 2001) and the affine transformation matrices were concatenated to generate a FLAIR-to-DTI transformation. Employing these transformations, TPMs were registered into DTI space. Hard segmentations were carried out to generate maps of tissue classes. This was done by voxel wise comparison of the different tissue probability maps, whereby voxels were allotted to the highest probability tissue class. Mask images of all WM were created based on the hard segmentation map. A histogram analysis was conducted on the MD, FA, RD and AxD maps in the WM regions. 5 WM summary measures (i.e., median, peak value, normalised peak height, skew and kurtosis) for each of the diffusion maps were obtained from normalized histograms with 1000 bins (MD range: 0–4 mm^2^s^-1^x 10^-3^, bin width: 0.004 mm^2^s^−1^ × 10^-3^; FA range: 0–1, bin width 0.001).

#### 3.2.2 The principal component measure (PC1)

The PC1 measure was computed using principal component analysis (PCA) on the 20 conventional WM histogram measures (Lê et al., 2008). PCA is an unsupervised learning method and linearly transforms variables to a lower dimensional space while retaining the maximal amount of information from the input data. The first principal component (PC1) captures the direction along which most variation in the multidimensional data is found und the underlying component scores were used as PC1 measure in each cohort.

#### 3.2.3 Peak width skeletonized mean diffusivity (PSMD)

The Peak width of skeletonized mean diffusivity **(**PSMD) is a fully automatically computed imaging marker publicly available (http://psmd-marker.com) which involves tensor fitting, skeletonizing the DTI data, applying a custom mask and computing a histogram analysis (Baykara et al., 2016, Behrens et al., 2003). The computation is divided into three main modules:

1) WM tract skeletonization using tract-based statistics (TBSS) and the FMRIB 1-mm fractional anisotropy template thresholded at an FA value of 0.2 (Smith et al., 2006). MD images were projected onto the skeleton employing the FA-derived projection parameters. In order to avoid contamination of the skeleton through CSF partial volume effects, MD skeletons were masked with a standard skeleton thresholded at an FA value of 0.3.
2) excluding CSF prone regions employing a custom mask. Brain structures adjacent to the ventricles such as the fornix were removed.
3) creating a histogram based on the MD values of voxels within the skeleton. The PSMD measure refers to the difference between the 5th and 95th percentiles of the histogram distribution.

#### 3.2.4 **Diffusion tensor image segmentation θ (DSEG** θ**)**

DSEG θ is a semiautomatic DTI marker that assigns an unique cerebral diffusion profile to each voxel that is based on the magnitudes of isotropic and anisotropic diffusion metrics (Williams et al., 2017). The processing pipeline is composed of 4 main modules:

1) excluding the cerebellum using an automated pipeline,
2) diffusion tensors fitting using ‘DTIFIT’ (Behrens et al., 2007, 2003),
3) k-median cluster analysis of the diffusion data to characterize each voxel as to belonging to one of the 16 segments that reflect the distinct magnitudes of anisotropy (q) and isotropy (p) microstructural diffusion properties,
4) calculating a DSEG summary score, θ, by comparing the volumetric spectrum of the 16 segments between patients in the form of a scalar product, θ, which computes the difference in whole brain diffusion characteristics in subjects compared to a reference brain. Clinical information of the patient's reference brain used in each cohort can be found in **eTable 4.**

##### 3.2.4.1 Global efficiency network measure (Geff)

Brain networks can be constructed using DTI tractography data, and a measure of global efficiency has been shown to predict dementia in a lacunar stroke cohort (Lawrence et al., 2018). Except for the network baseline data in RUN DMC (Tuladhar et al., 2016a), the construction of brain networks was performed using the same technique across all cohorts and the computation is summarized as follows:

1) for all cohort data 90 brain regions (80 cortical and 10 subcortical regions) defined from the AAL parcellation of the cerebral cortex and subcortical nuclei (Desikan et al., 2006) were employed as nodes, excluding those in the cerebellum.
2) employing “DTIFIT”, a diffusion tensor model was fitted to each voxel in all cohort data except for RUN DMC baseline (Jenkinson et al., 2012). In RUN DMC at baseline the diffusion tensor and FA was computed using the TrackVis Diffusion Toolkit (https://www.trackvis.org).
3) using ANTS (stnava.github.io/ANTs/), the standard space template FMRIB58_FA_1mm was registered to the patients FA maps in all cohort data except for RUN DMC at baseline. The AAL atlas was then transformed to the patient’s FA image using the nearest-neighbour interpolation method and the transformation matrix generated by the registration. In RUN DMC at baseline skull stripped T1-weighted images were nonlinearly registered to Montreal Neurological Institute (MNI) 152 template employing the Function MRI of the Brain nonlinear registration tool (FNIRT) (Andersson et al., 2007). The transformation matrix derived by registering the *b0*-images to T1-weighted subject space was used to register the AAL images to each patient’s diffusion image space with FLIRT (Jenkinson and Smith, 2001).
4) whole-brain deterministic tractography using MRtrix (Tournier et al., 2012) was performed on the principal eigenvector in all cohort data but RUN DMC at baseline. Streamlines (max number per voxel = 4, length = 20–250 nm, step size = 0.5 mm) were generated employing trilinear interpolation of the tensor field. Streamlines terminated in regions where FA<0.15 or the angle turn between consecutive vectors was greater than 45. Brain regions were classified as being linked to each other under the condition that at least one streamline terminating in region A also terminated in region B. The strength of connectivity between two seeds was determined by the streamline count adjusted by the length of the streamline in mm. In RUN DMC at baseline whole-brain deterministic tractography was applied using the fiber assignment by continuous tracking (FACT) algorithm (Tuladhar et al., 2016a). Fiber tracks were launched at the voxel’s center with FA greater than 0.2 and terminated either when leaving the brain mask, when coming across voxels with FA smaller than 0.2 or when the angle turn being greater than 60. Two brain regions were classified as being connected when the reconstructed streamline linked both regions. The weight of connectivity between two seeds was computed by multiplying the mean FA for each reconstructed streamline by the total number of reconstructed streamlines linking two regions. The number of reconstructed streamlines may correspond to the WM structure and has commonly been employed as weighting of edges in graph-theoretical studies. Under the condition that 2 edges share a similar number of streamlines, the weights may still be different when FA is considered. The connection strength was normalised by the volume of AAL regions and the differences in brain size (Brown et al., 2011).
5) based on the strength of connectivity, the connectome was reconstructed as an undirected graph and the adjacency matrix was generated as a symmetric matrix. Each matrix’s element indicated the strength of connectivity between individual pairs of brain regions. Graph-theory analysis was used to compute the summary measure weighted global network efficiency (Geff) (www.brain-connectivity-toolbox.net) (Latora and Marchiori, 2001, Rubinov and Sporns, 2010). The Geff measure indicates the degree of connectivity between nodes of the brain network relative to an idealized network where every node is connected.

### 3.3 Statistical analysis

Statistical analyses were carried out using R (version 3.6.3) with two-sided p values and p < 0.05 considered statistically significant. (R Core Team, 2019). No multiple comparisons adjustment was applied to the regression models.

#### 3.3.1 Cross-sectional analysis

Heatmaps were created reflecting the magnitude of Pearson correlation between the 20 WM DTI histogram measures in each cohort. Using MMSE and MOCA scores, boxplots were created to compare the patients’ profiles of impaired cognitive functions across the different cohorts. The association between the DTI measures and cognition was tested using linear regression while adjusting for age, sex and premorbid IQ or years of education completed. Education was log transformed in HARMONISATION to meet homoscedasticity and linearity assumptions. In the multi-centre PRESERVE study clinical site was added as a confounder into the linear model. In the CADASIL cohort one residual outlier observation was removed from the regression analysis to meet the statistical assumptions. This outlier was due to one DTI observation significantly altering the cross-sectional associations.

#### 3.3.2 Longitudinal analysis: Baseline imaging and dementia

The association between baseline imaging data and later dementia conversion was tested in SCANS, RUN DMC and HARMONISATION using a Cox proportional hazards model (Therneau and Lumley, 2015). The clinical markers such as age, sex and education or premorbid IQ were added as covariates. Follow-up time was used as the underlying time scale. We observed no violation of the proportional hazard assumption in any model. Receiver operating characteristic curves (ROC) were computed to show the diagnostic discriminatory ability of a binary classifier system with varying thresholds and the areas under the curves (AUCs) were calculated (Robin et al., 2011). In RUN DMC differences in baseline DTI measures between dementia subtypes (i.e., VD vs AD/VD vs AD) were tested using the Kruskal-Wallis test.

#### 3.3.3 Longitudinal analysis: Change in imaging measures and dementia

Repeat MRI at a later time point was available in SCANS, RUN DMC, HARMONISATION, CADASIL and PRESERVE and was used to determine the sensitivity of DTI measures to detect change. In ASPS-Fam the longitudinal sample size with DTI was too small (*N* = 64) for the longitudinal analysis of the small changes over time in a population-based sample. In the SCANS cohort with multiple follow-ups, a linear mixed model was fitted for MD Median, PSMD, DSEG θ and Geff (Bates et al., 2015). We included follow-up time as a fixed effect and included random intercepts and slopes for each participant. The average fixed effects slopes of time were interpreted as the average annualized change rate in a given imaging measure per additional year of follow-up. The statistical significance of change in DTI measures was determined using a paired *t*-test in all cohorts with 1 follow-up time point. In PRESERVE it was also tested as to whether there were differences in the temporal change of DTI measures between scanner sites while accounting for the marker’s baseline measure using an ANCOVA model with permutation (Wheeler, 2010).

To create a compound longitudinal DTI measure for the dementia analysis, a PCA was applied to the changes of 19 DTI markers used at baseline in SCANS and to all 20 DTI markers in RUN DMC and HARMONISATION (Lê et al., 2008). In SCANS no individual trajectories of AxD Median could be estimated due to limited variability in the data when using a linear mixed model. Prior to the Cox regression in SCANS, imaging measures collected post dementia diagnosis were removed and the linear mixed model was recomputed. Running a Cox regression in SCANS or logistic regression models in RUN DMC and HARMONISATION, the association between the change in DTI measures and dementia conversion was tested. ROC curves were modelled to estimate the diagnostic discriminatory ability of each predictive model and the AUCs were calculated (Robin et al., 2011).

#### 3.3.4 Sample size estimation

In the SCANS cohort with multiple follow-ups, a sample size estimation for a hypothetical clinical trial that applied each DTI marker was performed while varying the treatment effect sizes using the longpower R package (Donohue et al., 2013) for a statistical power of 0.80 and two-tailed type I error rate of 0.05. In cohorts with only one follow-up time point the imaging marker’s sample size was estimated based on the coefficient of variation with the same treatment effect sizes (Chen and Peace, 2010).

#### 3.3.5 Data availability statement

The data that support the findings of this study are available to *bona fide* researchers upon reasonable request subject to approval of the relevant regulatory and ethical bodies.

## 4 Results

### 4.1 Cognition

The degree of cognitive impairment was more in SCANS and CADASIL than RUN-DMC or ASPS-Fam as evidenced by a lower median MMSE. (eFigure 1, panel A). In HARMONISATION, patients who were more cognitively impaired had more consistently lower MOCA test scores than in PRESERVE (eFigure 1, panel B). In SCANS, RUN DMC and HARMONISATION, patients converting to dementia had overall lower baseline MMSE and MOCA test scores than patients who did not develop dementia (eFigure 1, panel C & panel D). The differences were stronger in SCANS and HARMONISATION than in RUN DMC.

### 4.2 PCA analysis

The percentage of explained variance of PC1 was highest in the CADASIL (84.4 %) group and lowest in ASPS-Fam (40 %) **(eFigure 2, eFigure 3**). In the sporadic SVD cohorts the PC1′s explained variance ranged between 57.2 and 71.5 % (**eFigure 2**). There were no patterns showing that dementia cases or dementia subtypes would be better represented on the second instead of the first principal component dimension (eFigure 4).

### 4.3 Cross-sectional results

There were significant associations between all five imaging measures and cognitive function in all SVD cohorts and in the MCI cohorts (Table 3a, Table 3b, Table 4, Table 5, Table 6, Table 7**, eTable 5a-f**). There was not a single regression model resulting in the highest explained variance (*Adj. R^2^*) and in the best model fit (AIC) across cohorts. In the single-center sporadic SVD cohorts SCANS and RUN DMC the models’ adjusted explained variances (*Adj. R^2^*) were similar within each cohort for all parameters (Table 3a). In the multicenter study PRESERVE the *Adj. R^2^* was lower in DSEG θ and Geff than in PC1, PSMD or MD Median (Table 3b). In CADASIL, PSMD explained more variance than any other measure. The overall model fit measured by the *AIC* was best for PSMD in PRESERVE, RUN DMC and CADASIL. The Geff model had the best model fit for SCANS and ASPS-Fam.

**Table 3a:** **Cross-sectional analysis between DTI and Global Cognition while accounting for clinical markers.** All imaging markers were associated with impaired cognitive function in the severe SVD (SCANS), mild SVD (RUN DMC) and MCI cohort (HARMONISATION).

	**Global Cognition**											
	*SCANS*				*RUN DMC*				*HARMONISATION*			
*Baseline* *Markers*	*β* *(95 % CI)*	*P-Value*	Adjusted R^2^	*AIC*	*β* *(95 % CI)*	*P-Value*	Adjusted R^2^	*AIC*	*β* *(95 % CI)*	*P-Value*	Adjusted R^2^	*AIC*
MD median	−0.25 (-0.38, −0.12)	**<0.001**	0.518	245.19	−0.22 (-0.31, -0.14)	**<0.001**	0.450	981.32	−0.31 (-0.47, −0.15)	**<0.001**	0.417	296.58
PC1	−0.30 (-0.43, -0.18)	**<0.001**	0.546	238.33	−0.25 (-0.34, -0.16)	**<0.001**	0.455	977.51	−0.33 (-0.49, −0.17)	**<0.001**	0.424	294.92
PSMD	−0.30 (-0.42, -0.17)	**<0.001**	0.545	238.67	−0.24 (-0.33, -0.16)	**<0.001**	0.456	976.24	−0.33 (-0.47, −0.18)	**<0.001**	0.437	291.98
DSEG θ	−0.38 (-0.53, -0.23)	**<0.001**	0.556	235.75	−0.12 (-0.22, -0.02)	**0.02**	0.424	1001.23	−0.44 (-0.62, −0.25)	**<0.001**	0.444	290.44
Geff	0.35 (0.22, 0.48)	**<0.001**	0.569	232.40	0.22 (0.14, 0.31)	**<0.001**	0.451	980.45	0.15 (0.01, 0.30)	**0.04**	0.368	306.64

MD Median = mean diffusivity median of the WM histogram, PC1 = scores of the first principal component, PSMD = peak width of skeletonized mean diffusivity, DSEG θ = diffusion tensor image segmentation θ, Geff = global efficiency network measure, β = standardized regression coefficient, 95 % CI = 95 % confidence interval, AIC = Akaike information criterion, P-Value = statistical value of significance with p < 0.05.

**Table 3b:** **Cross-sectional analysis between DTI and Global Cognition or TMT-B while accounting for the clinical markers.** In severe SVD and monogenic SVD all imaging markers were associated with impaired cognitive function. Only MD median, DSEG θ and Geff were significantly related to impaired cognitive function in the community cohort with normal neurological functioning.

	**Global Cognition**								**TMT-B**			
	*PRESERVE*				*ASPS-Fam*				*CADASIL*			
*Baseline* *Markers*	*β* *(95 % CI)*	*P-Value*	Adjusted R^2^	*AIC*	*β* *(95 % CI)*	*P-Value*	Adjusted R^2^	*AIC*	*β* *(95 % CI)*	*P-Value*	Adjusted R^2^	*AIC*
MD median	−0.39 (-0.57, −0.22)	**<0.001**	0.389	242.69	−0.14 (-0.24, −0.04)	**0.01**	0.516	488.48	−0.50 (-0.82, −0.18)	**<0.001**	0.227	133.15
PC1	0.37 (0.20, −0.54)	**<0.001**	0.375	245.01	−0.04 (-0.14, 0.06)	0.38	0.503	494.77	0.50 (0.15, 0.85)	**0.01**	0.206	134.50
PSMD	−0.41 (-0.57, −0.24)	**<0.001**	0.409	239.53	−0.03 (-0.13, 0.08)	0.64	0.501	495.32	−0.70 (-1.01, -0.39)	**<0.001**	0.357	124.11
DSEG θ	−0.19 (-0.37, −0.01)	**0.04**	0.282	258.78	−0.14 (-0.23, -0.05)	**<0.001**	0.521	486.35	−0.50 (-0.80, -0.20)	**<0.001**	0.251	131.65
Geff	−0.19 (-0.37, −0.01)	**0.04**	0.281	258.84	0.18 (0.09, 0.28)	**<0.001**	0.530	482.02	0.45 (0.09, 0.82)	**0.02**	0.243	136.43

MD Median = mean diffusivity median of the WM histogram, PC1 = scores of the first principal component, PSMD = peak width of skeletonized mean diffusivity, DSEG θ = diffusion tensor image segmentation θ, Geff = global efficiency network measure, β = standardized regression coefficient, 95 % CI = 95 % confidence interval, AIC = Akaike information criterion, P-Value = statistical value of significance with p < 0.05.

**Table 4:** **Baseline DTI measures predicting dementia conversion while accounting for the clinical markers.** Baseline PSMD and PC1 measures predicted dementia conversion in SCANS, RUN DMC and HARMONISATION. In RUN DMC MD median and DSEG was not associated with dementia. In HARMONISATION baseline Geff did not predict dementia conversion.

	**Dementia conversion**											
	*SCANS*				*RUN DMC*				*HARMONISATION*			
*Change* *Markers*	*HR* *(95 % CI)*	*P-Value*	*AIC*	*AUC*	*HR* *(95 % CI)*	*P-Value*	*AIC*	*AUC*	*HR* *(95 % CI)*	*P-Value*	*AIC*	*AUC*
MD Median	2.19 (1.51, 3.16)	**<0.001**	138.37	0.832	1.33 (1.00, 1.76)	0.05	515.69	0.828	1.78 (1.08, 2.93)	**0.02**	173.51	0.761
PC1	2.28 (1.51, 3.44)	**<0.001**	139.60	0.825	1.57 (1.15, 2.14)	**<0.001**	511.60	0.837	1.74 (1.03, 2.92)	**0.04**	174.17	0.765
PSMD	1.74 (1.29, 2.34)	**0.001**	143.70	0.804	1.45 (1.14, 1.83)	**<0.001**	511.51	0.831	1.73 (1.13, 2.65)	**0.01**	172.72	0.765
DSEG θ	3.52 (2.09, 5.92)	**<0.001**	128.21	0.908	1.33 (0.91, 1.93)	0.14	517.17	0.821	1.94 (1.06, 3.53)	**0.03**	173.67	0.753
Geff	0.37 (0.23, 0.61)	**<0.001**	138.39	0.842	0.64 (0.46, 0.89)	**0.01**	512.59	0.832	0.79 (0.49, 1.30)	0.36	177.58	0.702

MD Median = mean diffusivity median of the WM histogram, PC1 = scores of the first principal component, PSMD = peak width of skeletonized mean diffusivity, DSEG θ = diffusion tensor image segmentation θ, Geff = global efficiency network measure, AIC = Akaike information criterion, HR = hazard ratio, AUC = area under the curve, AIC = Akaike information criterion, 95 % CI = 95 % confidence interval, P-Value = statistical value of significance with p < 0.05.

**Table 5:** **Change in DTI measures in each cohort studies.** There were significant changes in all measures in all cohorts except for PRESERVE and CADASIL. In PRESERVE there was a significant change DSEG and MD Median but not for PSMD and Geff. In CADASIL there was only a marginally significant change for Geff.

	**Change in DTI over time**											
*Markers*	*PSMD*			*DSEG* θ			*Geff*			*MD Median*		
*Cohorts*	*Baseline*	*Change*	*P-Value*	*Baseline*	*Change*	*P-value*	*Baseline*	*Change*	*P-Value*	*Baseline*	*Change*	*P-Value*
SCANS a	3.78e-04 (3.58e-04, 3.98e-04)	1.40e-05 (1.03e-05, 1.77e-05)	**<0.001**	21.69 (19.90, 23.49)	1.61 (1.42, 1.80)	**<0.001**	8.12 (7.68, 8.57)	−0.18 (-0.23, −0.13)	**<0.001**	7.98e-04 **(**7.90e-04, 8.06e-04)	5.43e-06 (4.24e-06, 6.66e-06)	**<0.001**
RUN DMC b	3.04e-04 (8.29e-05)	2.67e-05 (2.64e-05)	**<0.001**	19.15 (8.83)	11.12 (5.84)	**<0.001**	10.38 (2.45)	−0.13 (0.94)	**0.02**	7.98e-04 (3.44e-05)	3.21e-06 (1.21e-05)	**<0.001**
HARMON ISATION b	3.60e-04 (7.71e-05)	2.76e-05 (4.04e-05)	**<0.001**	32.31 (8.24)	2.70 (3.33)	**<0.001**	0.41 (0.22)	−0.05 (0.12)	**<0.001**	8.82e-04 (6.08e-05)	2.24e-05 (3.62e-05)	**<0.001**
PRESERVE b	3.94e-04 (1.05e-04)	−8.77e-06 (5.59e-05)	0.16	47.83 (4.12)	8.54 (7.91)	**<0.001**	0.17 (0.11)	0.003 (0.137)	0.87	7.88e-04 (4.61e-05)	8.31e-06 (1.70e-05)	**<0.001**
CADASIL b	5.53e-04 (1.74e-04)	6.44e-05 (6.05e-05)	**<0.001**	22.00 (12.14)	9.09 (6.12)	**<0.001**	2.19 (2.04)	−0.15 (0.53)	0.05	8.82e-04 (1.21e-04)	6.73e-05 (4.78e-05)	**<0.001**

MD Median = mean diffusivity median of the WM histogram, PC1 = scores of the first principal component, PSMD = peak width of skeletonized mean diffusivity, DSEG θ = diffusion tensor image segmentation θ, Geff = global efficiency network measure.

P-Value = statistical value of significance with p < 0.05.

a linear mixed model in SCANS with the output: baseline intercept (95% confidence interval), estimated annual mean change (95% confidence interval) and p-value for each single imaging measure.

b paired *t*-test in RUN DMC, HARMONISATION, PRESERVE and CADASIL with the output: baseline mean (standard deviation), absolute mean change (standard deviation) between 2 time points and p-value for each single imaging measure.

**Table 6:** **Change in DTI measures associated with dementia conversion while accounting for the clinical markers.** Change in DTI was consistently associated with dementia conversion only in severe SVD but not in mild SVD or MCI. The AUC was highest and AIC lowest for DSEG. Change in MD Median was associated with dementia conversion in HARMONISATION.

	**Dementia conversion**											
	*SCANS*				*RUN DMC*				*HARMONISATION*			
*Change* *Markers*	*HR* *(95 % CI)*	*P-Value*	*AIC*	*AUC*	*OR* *(95 % CI)*	*P-Value*	*AIC*	*AUC*	*OR* *(95 % CI)*	*P-Value*	*AIC*	*AUC*
MD Median	2.46 (1.58, 3.83)	**<0.001**	134.67	0.789	0.90 (0.49–1.57)	0.72	83.37	0.892	1.60 (1.02, 2.63)	**0.04**	113.52	0.755
PC1	2.47 (1.52, 4.01)	**<0.001**	137.21	0.786	1.24 (0.57–2.46)	0.57	83.18	0.898	0.71 (0.44, 1.12)	0.14	115.58	0.733
PSMD	2.34 (1.60, 3.43)	**<0.001**	134.21	0.813	1.13 (0.65–1.79)	0.62	83.26	0.898	1.43 (0.91, 2.30)	0.11	115.28	0.743
DSEG θ	4.01 (2.21, 7.29)	**<0.001**	122.87	0.924	1.54 (0.78–3.28)	0.24	82.00	0.903	1.42 (0.87, 2.35)	0.16	115.77	0.723
Geff	0.49 (0.32, 0.75)	**<0.001**	140.24	0.793	0.76 (0.41–1.40)	0.36	82.68	0.897	1.06 (0.64, 1.75)	0.83	117.72	0.708

MD Median = Mean diffusivity Median of the aWM histogram, PC1 = Scores of the first principal component, PSMD = Peak width of skeletonized mean diffusivity, DSEG θ = Diffusion tensor image segmentation θ, Geff = Global efficiency network measure, AIC = Akaike information criterion, HR = hazard ratio, OR = Odd’s ratio, AUC = area under the cruve, AIC = Akaike information criterion, 95 % CI = 95 % confidence interval, P-Value = statistical value of significance with p < 0.05.

**Table 7:** **Sample size estimation per treatment arm**. Sample size estimation was lowest for DSEG and PSMD in the sporadic SVD cohorts SCANS and RUN DMC. DSEG and MD median required the lowest minimum sample size in CADASIL.

	SCANS			RUN DMC			HARMONISATION			CADASIL		
Duration of RCT	**3 years** **(3 follow-up measurements)**			**4 years** **(1 follow-up measurement)**			**2 years** **(1 follow-up measurement)**			**1.5 years** **(1 follow-up** **measurement)**		
Treatment effect	10 %	20 %	30 %	10 %	20 %	30 %	10 %	20 %	30 %	10 %	20 %	30 %
MD Median	1201	301	134	20,527	4650	1878	3795	860	348	732	166	67
PSMD	1073	269	120	1423	323	131	3101	703	284	1276	289	117
DSEG θ	417	105	47	1292	293	119	2200	499	202	656	149	60
Geff	2333	583	259	71,164	16,120	6510	8383	1899	767	19,068	4320	1745

RCT = Randomized controlled trial, MD Median = Mean diffusivity Median of the WM histogram, PC1 = Scores of the first principal component, PSMD = Peak width of skeletonized mean diffusivity, DSEG θ = Diffusion tensor image segmentation θ, Geff = Global efficiency network measure.

### 4.4 Longitudinal results

#### 4.4.1 Dementia prevalence and subtypes

In SCANS 18 out of 113 patients who had complete baseline DTI imaging measures converted to dementia within 5 years. In RUN DMC 435 patients had complete baseline DTI measures and 50 patients converted to dementia within 9 years (AD *N* = 28; VD *N* = 14; AD/VD *N* = 6; LBD *N* = 1; Unknown *N* = 1). In HARMONISATION 23 patients who had complete baseline DTI measures were diagnosed with dementia within 2 years (AD *N* = 20; VD *N* = 3). The rates of dementia conversion were higher in HARMONISATION than in SCANS or RUN DMC (eFigure 5).

#### 4.4.2 Baseline DTI in predicting future dementia conversion

**Fig. 1:**
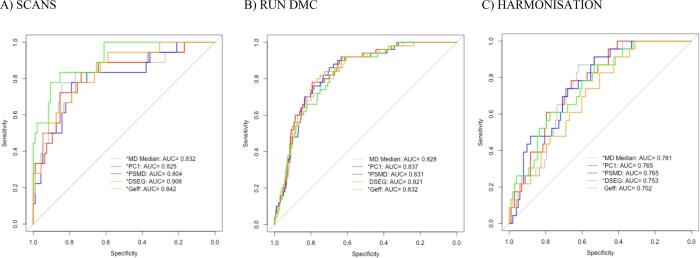
Baseline DTI measures together with the clinical markers, i.e. age, sex and education or premorbid IQ, classified dementia conversion vs no-dementia conversion better in the SVD cohorts, SCANS and RUN DMC, than in the MCI cohort HARMONISATION. MD Median = mean diffusivity median of the WM histogram, PC1 = scores of the first principal component, PSMD = peak width of skeletonized mean diffusivity, DSEG θ = diffusion tensor image segmentation θ, Geff = global efficiency network measure, AUC = area under the curve * the baseline measure significantly predicted dementia conversion independently of the clinical markers.

Complete imaging baseline data with follow-up dementia conversion were available for SCANS, RUN DMC and HARMONISATION (Table 2). Baseline PSMD and PC1 measures predicted dementia conversion in all 3 cohorts (Table 4**, eTable 6a-c**). In RUN DMC baseline MD median and DSEG θ were not associated with dementia, MD median: *HR* (95 % *CI*) = 1.33 (1.00, 1.76), DSEG θ: *HR* (95 % *CI*) = 1.33 (0.91, 1.93). In HARMONISATION baseline Geff did not predict dementia conversion, *HR* (95 % *CI*) = 0.79 (0.49, 1.30). The AUCs were overall higher in the sporadic SVD cohorts SCANS and RUN DMC than in the MCI cohort HARMONISATION (Fig. 1).

#### 4.4.3 Baseline DTI measures and subtypes of future dementia in RUN DMC

**Fig. 2:**
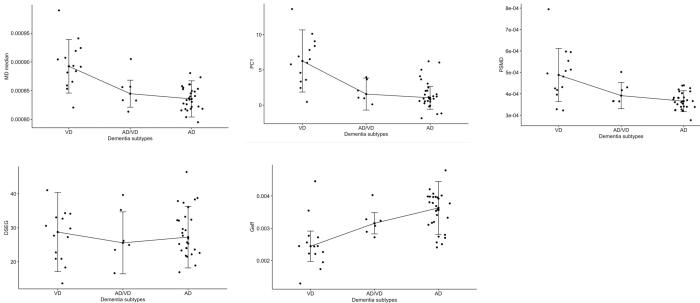
Differences in baseline DTI measures between subtypes of dementia conversion in RUN DMC. There were significant differences between VD and AD for all DTI measures except for DSEG. Median and interquartile range (IQR) were added to each dementia subtype. MD Median = mean diffusivity median of the WM histogram, PC1 = scores of the first principal component, PSMD = peak width of skeletonized mean diffusivity, DSEG θ = diffusion tensor image segmentation θ, Geff = global efficiency network measure, VD = vascular dementia, AD/VD = mixed Alzheimer’s and vascular dementia, AD = Alzheimer’s disease.

In RUN DMC there were significant differences between the subtypes for baseline PC1 (*χ^2^* = 17.19, *p* < 1.9e-04, *df* = 2), MD median (*χ^2^* = 19.00, *p* < 7.5e-05, *df* = 2), PSMD (*χ^2^* = 13.52, *p* = 0.001, *df* = 2) and Geff (*χ^2^* = 16.07, *p* = 3.2e-03, *df* = 2) (Fig. 2). For baseline DSEG no differences between the 3 subtypes were found (*χ^2^* = 0.07, *p* = 0.97, *df* = 2). Holm-Bonferroni multiple-comparison post hoc tests showed that baseline PC1 (*p* = 5.4e-05), MD median (*p* = 4.6e-05) and PSMD (*p* = 7.9e-04) were significantly higher and baseline Geff (*p* = 1.6e-04) significantly lower in VD than in the AD dementia subtype. Higher PC1, MD median, PSMD and lower Geff in VD than in AD were consistently found over 3-years intervals in RUN DMC (eFigure 6).

#### 4.4.4 Change in DTI measures over time

All imaging measures showed significant change over time except for PSMD and Geff in the multicentre PRESERVE cohort and Geff in HARMONISATION and CADASIL (Table 5). Changes in all conventional WM DTI histogram measures in SCANS, RUN DMC and HARMONISATION are shown in **eTables 8a-c**. Overall, most imaging markers significantly changed over time. PC1 explained less variation of the imaging change data in the sporadic SVD than in the MCI cohort (SCANS: 36.3 %, RUN DMC: 32.8 %, HARMONISATION: 57 %) (**eFigure 7**). In PRESERVE most conventional DTI histogram measures significantly changed and PC1 explained 31.9 % of the variance when running a PCA on all 20 conventional DTI measures (**eTable 7**). There were no differences between scanner sites for any changes in the 5 imaging measures while accounting for the measure’s baseline value and controlling for the increased Type-1 error rate with the Tukey post hoc test (**eFigure 8**).

#### 4.4.5 Change in DTI in predicting dementia conversion

Complete longitudinal imaging data with follow-up dementia conversion were available for SCANS, RUN DMC and HARMONISATION (Table 2). In SCANS all 5 imaging measures significantly changed over the 3 years after removing observations post dementia diagnosis (**eTable 9**). Changes in all imaging measures were associated with dementia conversion in SCANS, but not in RUN DMC (Table 6). In HARMONISATION change in MD Median was the only measure significantly associated with dementia conversion, *OR* (95 % *CI*) = 1.60 (1.02, 2.63). The association and model fit were strongest in the DSEG θ model in SCANS, *HR* (95 % *CI*) = 4.01 (2.21, 7.29), and also resulted in the highest *AUC* with 0.924 (Table 6). Across the 3 different cohorts, SCANS, RUN DMC and HARMONISATION, the combined measure PC1 was not a stronger predictor for dementia conversion than its underlying histogram measure MD median. Due to the low dementia incidence in RUN DMC relative to the overall cohort size (*N* = 12), a Firth's Bias-reduced logistic regression was additionally run on the RUN DMC data. In line with previous evidence, no significant association between any changes in DTI and dementia conversion was found (**eTable 10**).

#### 4.4.6 Sample size estimation for a hypothetical clinical trial

Sample sizes for a hypothetical clinical trial with varying durations and varying treatment effect sizes were calculated for the four single imaging measures in the different cohorts and are shown in Table 7. In both single-center sporadic SVD cohorts (SCANS, RUN DMC) DSEG θ and PSMD required the lowest sample size estimates. DSEG θ and MD median had the lowest sample size estimation in CADASIL. Sample size estimates were high for Geff in all 4 cohorts.

## 5 Discussion

An increasing number of studies have demonstrated that DTI measures of WM damage are associated with cognition, predict future dementia conversion and capture change over time. This has raised awareness of DTI as an attractive surrogate endpoint for clinical trials in SVD. However, how best to analyse DTI data remains uncertain. An ideal analysis technique would combine a simple automated technique, with high sensitivity to change and dementia conversion. In this study, we compared 5 different DTI analysis approaches by comparing associations of each with cognition at baseline, and with future dementia conversion.

We were not able to identify a single optimal analysis strategy. Except for the asymptomatic SVD cohort ASPS-Fam, all analysis techniques were significantly associated with global cognition or executive function at baseline. The overall fit of the regression model, measured by AIC, was worse for DSEG and Geff in PRESERVE and better for PSMD in CADASIL. In the 3 studies in which there was imaging and data on future dementia conversion, SCANS, RUN DMC and HARMONISATION, most baseline DTI measures predicted dementia. The area under the curve was larger in the SVD cohorts (SCANS & RUN DMC) compared to the MCI cohort (HARMONISATION). Baseline PC1 and PSMD predicted dementia conversion across all 3 cohorts. DSEG θ performed notably better in the SCANS cohort than in RUN DMC.

Differentiating between dementia subtypes in RUN DMC, all baseline DTI measures, except for DSEG, were significantly different between patients converting to VD dementia and those converting to AD dementia. The differences in DTI measure profiles indicated more severe WM ultrastructural damage in those converting to VD. Previous studies also showed different etio-pathogenic profiles of WM microstructure between VD and AD dementia (Fu et al., 2012, Tu et al., 2017). Whereas patients with SVD had global changes in DTI measures across many brain regions, patients with AD demonstrated more regionally confined microstructural WM changes.

There were significant changes in all DTI metrics over time in all single-center sporadic SVD and MCI cohorts. In the multicentre cohort PRESERVE, the network analysis and PSMD were not sensitive to change. The combined PC1 measure was not a better predictor for dementia conversion, as evidenced by AUC, than one of its underlying single measures, i.e., MD median. Despite the significant changes over time in all DTI measures, consistent associations with dementia conversion were only found in the severe SVD cohort, i.e., SCANS, and not in the 2 cohorts with AD dementia as the most prevalent subtype.

Since the analysis techniques were similar in their predictive ability, then ease of use and the degree of automation become important factors. PSMD allows for a fully automated analysis without any tissue segmentation, that may require checking and correction, making it suitable for rapid analysis of large data sets. PSMD performed well across all cohorts except for detecting change in the PRESERVE cohort. In the PRESERVE cohort 86% had been scanned on a Philips scanner. The sensitivity of PSMD has been shown to be affected by images from the Philips scanner especially when there was a software update during the study (https://www.psmd-marker.com/faq/) and this may be a limitation. Another automatic skeletonized measure, called Mean Skeletonized Mean Diffusivity (MSMD), was sensitive to change in PRESERVE (**eFigure 9**). MSMD may therefore offer a better marker than PSMD when using Philips scanners. A further advantage of PSMD is that it is freely available. DSEG θ is semi-automated method requiring some user input such as choosing the reference brain. It performed well across datasets and may also be useful for use in clinical trials. However, it is not yet openly available.

We studied patient cohorts with a range of SVD, from mild changes in a stroke and dementia free community population (ASPS-Fam), through to patients with severe symptomatic SVD with confluent WMH (SCANS) and patients with monogenic SVD (CADASIL). The previous OPTIMAL study showed that changes in WM microstructure may contribute to cognitive impairment in all cohorts and in those cohorts in which data on future dementia risk was available predicted dementia (Egle et al., 2022). This emphasizes a key role for WM microstructure and cognitive decline across the range of disease severity, not only in pure SVD, but also in an MCI cohort in whom the predominant future dementia type is AD, and the utility of DTI metrics in capturing this.

It was notable that in PRESERVE the amount of variance in cognition explained by DTI metrics was lower. This is likely to reflect the multicentre nature of PRESERVE, in which DTI was performed on several different scanners across different sites. In contrast the other studies utilized imaging on a single scanner. To solve these challenges associated with acquisition-related differences of diffusion MRI, a promising DTI harmonization technique has been successfully tested on various SVD cohorts (de Brito Robalo et al., 2021). Ideally, however, in a clinical trial harmonization, or even standardization, should be implemented at the level of the acquisition.

We used the data from this study to estimate sample size calculations for a trial in SVD. With a treatment effect of 20 %, which would be typical for many intervention studies, sample sizes of a few hundred would be required. Sample sizes were generally low for DSEG θ, and only slightly higher for PSMD. In contrast, the network measure Geff needed a high minimum sample size in all 4 cohorts.

The study has several strengths. We compared several different DTI parameters across multiple independent datasets. Analysis methods were evaluated on additional datasets on which they had not been developed. However, it also has limitations. Firstly, different durations and number of follow-up time points were used which may have affected the findings across cohorts with varying SVD severity. Strong associations between DTI changes and dementia conversion were only found in the cohort characterized by more severe sporadic SVD progression, with multiple observational follow-up points. Annual follow-up time points in RUN DMC may have resulted in similar findings as in SCANS. Second, different MRI acquisition parameters were employed for the different cohorts potentially affecting the results. Third, different DTI pre-processing has been applied for different cohorts, nevertheless the results were relatively consistent between the cohorts. The Geff generated from the RUN DMC data differs in scale to that from the other datasets, this is most likely due to the differences in processing between this dataset and those processed centrally. While this may be a limitation, we felt it important to preserve the compatibility between the data published here and in other papers using it. Fourth, these DTI measures were all based on single-shell DTI. However, multi-shell DTI and higher DTI models may be more informative to characterize the WM microstructure, perhaps leading to lower sample sizes.

A further consideration is that we included a range of severity of disease pathologies in our study. Some represented severe SVD in whom the predominant pathology which may cause dementia is vascular, and this was reflected in the dementia cases in SCANS being vascular. In others, such as the MCI cohort, the predominant dementia pathology is likely to be AD, although the strong prediction provided by DTI suggests that WM changes are also important. Consistent with this, most dementia cases were AD. Interestingly in RUN DMC in which the inclusion criteria were symptomatic SVD due to stroke, cognitive or motor symptoms, most dementia cases were AD emphasizing that in elderly populations mixed pathology is the major pathology underlying dementia.

In conclusion, all DTI approaches at baseline predict future dementia risk in SVD and may be useful surrogate endpoints in clinical trials of SVD. We found no clear difference between different DTI strategies but automated methods such as PSMD offer clear advantages in large studies. PSMD performed well overall except for the PRESERVE dataset, possibly due to difficulties in using it on Philips sequences. DSEG θ performed well overall. Whilst these methods did not show marked improvement over simple histogram methods, they are easier to implement in large clinical studies.

## Funding

This work was funded by a grant from Alzheimer’s Research UK (ARUK-PG2016A-1). Additional support was provided by a Cambridge University- LMU collaborative grant. ME is funded by a Priority Programme Grant from the Stroke Association (2015–02). Infrastructural support was provided by National Institute of Health Research (NIHR) Cambridge Biomedical Research Centre Dementia and Neurodegeneration Theme (146281) and the Cambridge BHF Centre of Research Excellence [RE/18/1/34212]. SB and HSM are supported by a British Heart Foundation Programme Grant (RG/16/4/32218). HSM is also supported by an NIHR Senior Investigator Award. The views expressed are those of the author(s) and not necessarily those of the NHS, NIHR or the Department of Health and Social Care. The funding organisations had no role in any of the following: design and conduct of the study; collection, management, analysis, and interpretation of the data; review, or approval of the manuscript.

This work is also supported by BrightFocus foundation (reference no. A2018165F), National Medical Research Council Transition Award (R-608–000-342–213) and Ministry of Education Tier 1 grant (R-608–000-311–114) awarded to SH.

FE de Leeuw is supported by a clinical established investigator grant from the Dutch Heart Foundation (2014 T060) and by a VIDI innovational grant from The Netherlands ZonMw (Grant No 016126351). AMT has received a grant from the Junior Staff Member Dutch Heart Foundation (2016 T044). The funders and sponsors played no role in study design or analysis. C Chen is supported by a Singapore Translational Research award from the National Research Council of Singapore.

Authors take full responsibility for the data, the analyses and interpretation, and the conduct of the research; full access to all of the data; and the right to publish any and all data. ME and HSM are study guarantors.

## 7 Ethics approval

All studies were carried out in accordance with The Code of Ethics of the World Medical Association (Declaration of Helsinki) for experiments involving humans. All studies were approved by the ethics committees of the respective institutions. SCANS was approved by the London–Wandsworth ethics committee (ukctg.nihr.ac.uk; study iD: 4577). RUN DMC was approved by the Medical Review Ethics Committee region Arnhem-Nijmegen (No. 2005/256). HARMONISATION was approved by the National Healthcare Group Domain-Specific Review Board (DSRB reference No. 2010/00017). PRESERVE was approved by the Harrow National Research Ethics Service committee (No: 11/LO/0458). ASPS-Fam was approved by the ethics committee of the Medical University of Graz, Austria (comission registration No. IRB00002556). CADASIL was approved by the LMU medical faculty (No. 299/03).

## CRediT authorship contribution statement

**Marco Egle:** Conceptualization, Methodology, Formal analysis, Writing – original draft, Writing – review & editing, Project administration. **Saima Hilal:** Conceptualization, Investigation, Writing – review & editing. **Anil M Tuladhar:** Conceptualization, Investigation, Writing – review & editing. **Lukas Pirpamer:** Conceptualization, Investigation, Writing – review & editing. **Steven Bell:** Formal analysis, Data curation, Writing – review & editing. **Edith Hofer:** Investigation, Writing – review & editing. **Marco Duering:** Conceptualization, Investigation, Software, Writing – review & editing, Funding acquisition. **James Wason:** Formal analysis, Data curation, Writing – review & editing. **Robin G Morris:** Conceptualization, Formal analysis, Writing – review & editing. **Martin Dichgans:** Conceptualization, Investigation, Writing – review & editing, Funding acquisition. **Reinhold Schmidt:** Conceptualization, Investigation, Writing – review & editing, Funding acquisition. **Daniel J Tozer:** Conceptualization, Data curation, Software, Writing – review & editing, Supervision. **Thomas R. Barrick:** Data curation, Software, Writing – review & editing. **Christopher Chen:** Conceptualization, Investigation, Writing – review & editing, Funding acquisition. **Frank-Erik de Leeuw:** Conceptualization, Investigation, Writing – review & editing, Funding acquisition. **Hugh S Markus:** Conceptualization, Methodology, Formal analysis, Writing – original draft, Writing – review & editing, Supervision, Funding acquisition.

## Declaration of Competing Interest

The authors declare that they have no known competing financial interests or personal relationships that could have appeared to influence the work reported in this paper.

## References

[b0005] Amin Al Olama, A., Wason, J.M.S., Tuladhar, A.M., van Leijsen, E.M.C., Koini, M., Hofer, E., Morris, R.G., Schmidt, R., de Leeuw, F.E., Markus, H.S., 2020. Simple MRI score aids prediction of dementia in cerebral small vessel disease. Neurology 94, e1294–e1302. Doi: 10.1212/WNL.0000000000009141. 10.1212/WNL.0000000000009141 PMC7274929 32123050

[b0010] Andersson, J.L.R., Jenkinson, M., Smith, S., 2007. Non-linear registration aka spatial normalisation, FMRIB Technical Report TRO7JA2.

[b0015] Andersson J.L.R., Sotiropoulos S.N. (2016). An integrated approach to correction for off-resonance effects and subject movement in diffusion MR imaging. Neuroimage.

[b0020] Ashburner J., Friston K.J. (2005). Unified segmentation. Neuroimage.

[b0025] Bates D., Mächler M., Bolker B.M., Walker S.C. (2015). Fitting linear mixed-effects models using lme4. J. Stat. Softw..

[b0030] Baykara E., Gesierich B., Adam R., Tuladhar A.M., Biesbroek J.M., Koek H.L., Ropele S., Jouvent E., Chabriat H., Ertl-Wagner B., Ewers M., Schmidt R., de Leeuw F.-E., Biessels G.J., Dichgans M., Duering M. (2016). A Novel Imaging Marker for Small Vessel Disease Based on Skeletonization of White Matter Tracts and Diffusion Histograms. Ann. Neurol..

[b0035] Behrens T.E.J., Woolrich M.W., Jenkinson M., Johansen-Berg H., Nunes R.G., Clare S., Matthews P.M., Brady J.M., Smith S.M. (2003). Characterization and Propagation of Uncertainty in Diffusion-Weighted MR Imaging. Magn. Reson. Med..

[b0040] Behrens T.E.J., Berg H.J., Jbabdi S., Rushworth M.F.S., Woolrich M.W. (2007). Probabilistic diffusion tractography with multiple fibre orientations: What can we gain?. Neuroimage.

[b0045] Benjamin P., Zeestraten E., Lambert C., Chis Ster I., Williams O.A., Lawrence A.J., Patel B., Mackinnon A.D., Barrick T.R., Markus H.S. (2016). Progression of MRI markers in cerebral small vessel disease: Sample size considerations for clinical trials. J. Cereb. Blood Flow Metab..

[b0050] Brown J.A., Terashima K.H., Burggren A.C., Ercoli L.M., Miller K.J., Small G.W., Bookheimer S.Y. (2011). Brain network local interconnectivity loss in aging APOE-4 allele carriers. Proc. Natl. Acad. Sci. U.S.A..

[b0055] Chen, D.-G. (Din), Peace, K.E., 2010. Clinical Trial Data Analysis Using R, Clinical Trial Data Analysis Using R. Doi: 10.1201/b10478.

[b0060] Croall I.D., Lohner V., Moynihan B., Khan U., Hassan A., Brien J.T.O., Robin G., Tozer D.J., Cambridge V.C., Harkness K., Werring D.J., Blamire A.M., Ford G.A., Barrick T.R., Markus H.S., O’Brien J.T., Morris R.G., Tozer D.J., Cambridge V.C., Harkness K., Werring D.J., Blamire A.M., Ford G.A., Barrick T.R., Markus H.S. (2017). Using DTI to assess white matter microstructure in cerebral small vessel disease (SVD) in multicentre studies. Clin. Sci..

[b0065] Davies, G., Lam, M., Harris, S.E., Trampush, J.W., Luciano, M., Hill, W.D., Hagenaars, S.P., Ritchie, S.J., Marioni, R.E., Fawns-Ritchie, C., Liewald, D.C.M., Okely, J.A., Ahola-Olli, A. V., Barnes, C.L.K., Bertram, L., Bis, J.C., Burdick, K.E., Christoforou, A., Derosse, P., Djurovic, S., Espeseth, T., Giakoumaki, S., Giddaluru, S., Gustavson, D.E., Hayward, C., Hofer, E., Ikram, M.A., Karlsson, R., Knowles, E., Lahti, J., Leber, M., Li, S., Mather, K.A., Melle, I., Morris, D., Oldmeadow, C., Palviainen, T., Payton, A., Pazoki, R., Petrovic, K., Reynolds, C.A., Sargurupremraj, M., Scholz, M., Smith, J.A., Smith, A. V., Terzikhan, N., Thalamuthu, A., Trompet, S., Van Der Lee, S.J., Ware, E.B., Windham, B.G., Wright, M.J., Yang, J., Yu, J., Ames, D., Amin, N., Amouyel, P., Andreassen, O.A., Armstrong, N.J., Assareh, A.A., Attia, J.R., Attix, D., Avramopoulos, D., Bennett, D.A., Böhmer, A.C., Boyle, P.A., Brodaty, H., Campbell, H., Cannon, T.D., Cirulli, E.T., Congdon, E., Conley, E.D., Corley, J., Cox, S.R., Dale, A.M., Dehghan, A., Dick, D., Dickinson, D., Eriksson, J.G., Evangelou, E., Faul, J.D., Ford, I., Freimer, N.A., Gao, H., Giegling, I., Gillespie, N.A., Gordon, S.D., Gottesman, R.F., Griswold, M.E., Gudnason, V., Harris, T.B., Hartmann, A.M., Hatzimanolis, A., Heiss, G., Holliday, E.G., Joshi, P.K., Kähönen, M., Kardia, S.L.R., Karlsson, I., Kleineidam, L., Knopman, D.S., Kochan, N.A., Konte, B., Kwok, J.B., Le Hellard, S., Lee, T., Lehtimäki, T., Li, S.C., Liu, T., Koini, M., London, E., Longstreth, W.T., Lopez, O.L., Loukola, A., Luck, T., Lundervold, A.J., Lundquist, A., Lyytikäinen, L.P., Martin, N.G., Montgomery, G.W., Murray, A.D., Need, A.C., Noordam, R., Nyberg, L., Ollier, W., Papenberg, G., Pattie, A., Polasek, O., Poldrack, R.A., Psaty, B.M., Reppermund, S., Riedel-Heller, S.G., Rose, R.J., Rotter, J.I., Roussos, P., Rovio, S.P., Saba, Y., Sabb, F.W., Sachdev, P.S., Satizabal, C.L., Schmid, M., Scott, R.J., Scult, M.A., Simino, J., Slagboom, P.E., Smyrnis, N., Soumaré, A., Stefanis, N.C., Stott, D.J., Straub, R.E., Sundet, K., Taylor, A.M., Taylor, K.D., Tzoulaki, I., Tzourio, C., Uitterlinden, A., Vitart, V., Voineskos, A.N., Kaprio, J., Wagner, M., Wagner, H., Weinhold, L., Wen, K.H., Widen, E., Yang, Q., Zhao, W., Adams, H.H.H., Arking, D.E., Bilder, R.M., Bitsios, P., Boerwinkle, E., Chiba-Falek, O., Corvin, A., De Jager, P.L., Debette, S., Donohoe, G., Elliott, P., Fitzpatrick, A.L., Gill, M., Glahn, D.C., Hägg, S., Hansell, N.K., Hariri, A.R., Ikram, M.K., Jukema, J.W., Vuoksimaa, E., Keller, M.C., Kremen, W.S., Launer, L., Lindenberger, U., Palotie, A., Pedersen, N.L., Pendleton, N., Porteous, D.J., Räikkönen, K., Raitakari, O.T., Ramirez, A., Reinvang, I., Rudan, I., Rujescu, D., Schmidt, R., Schmidt, H., Schofield, P.W., Schofield, P.R., Starr, J.M., Steen, V.M., Trollor, J.N., Turner, S.T., Van Duijn, C.M., Villringer, A., Weinberger, D.R., Weir, D.R., Wilson, J.F., Malhotra, A., McIntosh, A.M., Gale, C.R., Seshadri, S., Mosley, T.H., Bressler, J., Lencz, T., Deary, I.J., 2018. Study of 300,486 individuals identifies 148 independent genetic loci influencing general cognitive function. Nat. Commun. https://doi.org/10.1038/s41467-018-04362-x.

[b0070] de Brito Robalo, B.M., Jan Biessels, G., Chen, C., Dewenter, A., Duering, M., Hilal, S., Koek, H.L., Kopczak, A., Yin Ka Lam, B., Leemans, A., Mok, V., Onkenhout, L.P., van den Brink, H., de Luca, A., 2021. Diffusion MRI harmonization enables joint-analysis of multicentre data of patients with cerebral small vessel disease. NeuroImage Clin. 32, 102886. https://doi.org/10.1016/j.nicl.2021.102886. 10.1016/j.nicl.2021.102886 PMC8609094 34911192

[b0075] Debette S., Markus H.S. (2010). The clinical importance of white matter hyperintensities on brain magnetic resonance imaging: Systematic review and meta-analysis. BMJ.

[b0080] Debette S., Schilling S., Duperron M.-G., Larsson S.C., Markus H.S. (2019). Clinical Significance of Magnetic Resonance Imaging Markers of Vascular Brain Injury: A Systematic Review and Meta-analysis. JAMA Neurol.

[b0085] Desikan R.S., Ségonne F., Fischl B., Quinn B.T., Dickerson B.C., Blacker D., Buckner R.L., Dale A.M., Maguire R.P., Hyman B.T., Albert M.S., Killiany R.J. (2006). An automated labeling system for subdividing the human cerebral cortex on MRI scans into gyral based regions of interest. Neuroimage.

[b0090] Donohue M.C., Gamst A.C., Edland S.D., Donohue M.M.C. (2013). Package ‘longpower’. Biometrics.

[b0095] Egle M., Hilal S., Tuladhar A.M., Pirpamer L., Hofer E., Duering M., Wason J., Morris R.G., Dichgans M., Schmidt R., Tozer D., Chen C., de Leeuw F.-E., Markus H.S. (2022). Prediction of dementia using diffusion tensor MRI measures: the OPTIMAL collaboration. J. Neurol. Neurosurg. Psychiatry.

[b0100] Erkinjuntti, T., 1994. Clinical criteria for vascular dementia the NINDS-AIREN criteria, in: Dementia. Doi: 10.1159/000106721. 10.1159/000106721 8087178

[b0105] Fazekas F., Chawluk J.B., Alavi A., Hurtig H.I., Zimmerman R.A. (1987). MR signal abnormalities at 1.5 T in Alzheimer’s dementia and normal aging. Am. J. Roentgenol..

[b0110] Folstein M.F., Folstein S.E., McHugh P.R. (1975). “Mini-mental state”. A practical method for grading the cognitive state of patients for the clinician. J. Psychiatr. Res..

[b0115] Fu J.L., Zhang T., Chang C., Zhang Y.Z., Li W.B. (2012). The value of diffusion tensor imaging in the differential diagnosis of subcortical ischemic vascular dementia and Alzheimer’s disease in patients with only mild white matter alterations on T2-weighted images. Acta radiol..

[b0120] Hilal S., Chai Y.L., van Veluw S., Shaik M.A., Ikram M.K., Venketasubramanian N., Richards A.M., Biessels G.J., Chen C. (2017). Association between subclinical cardiac biomarkers and clinically manifest cardiac diseases with cortical cerebral microinfarcts. JAMA Neurol.

[b0125] Jenkinson M., Beckmann C.F., Behrens T.E.J., Woolrich M.W., Smith S.M. (2012). Review FSL. Neuroimage.

[b0130] Jenkinson M., Smith S. (2001). A global optimisation method for robust affine registration of brain images. Med. Image Anal..

[b0135] Latora V., Marchiori M. (2001). Efficient behavior of small-world networks. Phys. Rev. Lett..

[b0140] Lawrence A.J., Chung A.W., Morris R.G., Markus H.S., Barrick T.R. (2014). Structural network efficiency is associated with cognitive impairment in small-vessel disease. Neurology.

[b0145] Lawrence, A.J., Brookes, R.L., Zeestraten, E.A., Barrick, T.R., Morris, R.G., Markus, H.S., 2015. Pattern and rate of cognitive decline in cerebral small vessel disease: A prospective study. PLoS One 10, 1–15. Doi: 10.1371/journal.pone.0135523. 10.1371/journal.pone.0135523 PMC4537104 26273828

[b0150] Lawrence, A.J., Zeestraten, E.A., Benjamin, P., Lambert, C.P., Morris, R.G., Barrick, T.R., Markus, H.S., 2018. Longitudinal decline in structural networks predicts dementia in cerebral small vessel disease. Neurology 90, e1898–e1910. Doi: 10.1212/WNL.0000000000005551. 10.1212/WNL.0000000000005551 PMC5962914 29695593

[b0155] Lawrence A.J., Patel B., Morris R.G., MacKinnon A.D., Rich P.M., Barrick T.R., Markus H.S., Baron J.-C. (2013). Mechanisms of Cognitive Impairment in Cerebral Small Vessel Disease: Multimodal MRI Results from the St George’s Cognition and Neuroimaging in Stroke (SCANS) Study. PLoS One.

[b0160] Lawrence A.J., Zeestraten E.A., Morris R.G., Lambert C., Markus H.S., Benjamin P., Mackinnon A.D., Barrick T.R., Brookes R.L. (2017). Change in multimodal MRI markers predicts dementia risk in cerebral small vessel disease. Neurology.

[b0165] Lê, S., Josse, J., Husson, F., 2008. FactoMineR: An R package for multivariate analysis. J. Stat. Softw. https://doi.org/10.18637/jss.v025.i01.

[b0170] Markus H.S., Egle M., Croall I.D., Sari H., Khan U., Hassan A., Harkness K., MacKinnon A., O’Brien J.T., Morris R.G., Barrick T.R., Blamire A.M., Tozer D.J., Ford G.A., Birns J., Davies J., Barkat A., Cappuccio F., Robinson T., Grey L., Briley D., Bhalla A., Hollocks M.J., Davies L.A., Cambridge V.C., Moynihan B., Tripper S., Dixit A., Davies J., Davis M., Ford G., Dafe C., McGirr J., Spillane M., Waugh D., Ellison-Handley B., Werring D., Banara A. (2021). PRESERVE: Randomized Trial of Intensive Versus Standard Blood Pressure Control in Small Vessel Disease. Stroke.

[b0175] McKhann G.M., Knopman D.S., Chertkow H., Hyman B.T., Jack C.R., Kawas C.H., Klunk W.E., Koroshetz W.J., Manly J.J., Mayeux R., Mohs R.C., Morris J.C., Rossor M.N., Scheltens P., Carrillo M.C., Thies B., Weintraub S., Phelps C.H. (2011). The diagnosis of dementia due to Alzheimer’s disease: Recommendations from the National Institute on Aging-Alzheimer’s Association workgroups on diagnostic guidelines for Alzheimer’s disease. Alzheimer’s Dement.

[b0180] Nam K.-W., Kwon H.-M., Lim J.-S., Han M.-K., Nam H., Lee Y.-S., Wang X. (2017). The presence and severity of cerebral small vessel disease increases the frequency of stroke in a cohort of patients with large artery occlusive disease. PLoS One.

[b0185] Nasreddine Z.S., Phillips N.A., Bédirian V., Charbonneau S., Whitehead V., Collin I., Cummings J.L., Chertkow H. (2005). The Montreal Cognitive Assessment, MoCA: a brief screening tool for mild cognitive impairment. J. Am. Geriatr. Soc..

[b0190] Nitkunan A., Barrick T.R., Charlton R.A., Clark C.A., Markus H.S. (2008). Multimodal MRI in Cerebral Small Vessel Disease. Stroke.

[b0195] O’Sullivan M., Morris R.G., Huckstep B., Jones D.K., Williams S.C.R., Markus H.S. (2004). Diffusion tensor MRI correlates with executive dysfunction in patients with ischaemic leukoaraiosis. J. Neurol. Neurosurg. Psychiatry..

[b0200] Pantoni L. (2010). Cerebral small vessel disease: from pathogenesis and clinical characteristics to therapeutic challenges. Lancet Neurol..

[b0205] Pasi M., Van Uden I.W.M., Tuladhar A.M., De Leeuw F.E., Pantoni L. (2016). White Matter Microstructural Damage on Diffusion Tensor Imaging in Cerebral Small Vessel Disease: Clinical Consequences. Stroke.

[b0210] Power M.C., Su D., Wu A., Reid R.I., Jack C.R., Knopman D.S., Coresh J., Huang J., Kantarci K., Sharrett A.R., Gottesman R.G., Griswold M.E., Mosley T.H. (2019). Association of white matter microstructural integrity with cognition and dementia. Neurobiol. Aging..

[b0215] Prins N.D., van Dijk E.J., den Heijer T., Vermeer S.E., Koudstaal P.J., Oudkerk M., Hofman A., Breteler M.M.B. (2004). Cerebral white matter lesions and the risk of dementia. Arch. Neurol..

[b0220] R Core Team (2019).

[b0225] Robin X., Turck N., Hainard A., Tiberti N., Lisacek F., Sanchez J.-C., Müller M. (2011). pROC: An open-source package for R and S+ to analyze and compare ROC curves. BMC Bioinformatics.

[b0230] Rubinov M., Sporns O. (2010). Complex network measures of brain connectivity: uses and interpretations. Neuroimage.

[b0235] Schmidt R., Scheltens P., Erkinjuntti T., Pantoni L., Markus H.S., Wallin A., Barkhof F., Fazekas F. (2004). White matter lesion progression: A surrogate endpoint for trials in cerebral small-vessel disease. Neurology.

[b0240] Schneider J.A., Arvanitakis Z., Bang W., Bennett D.A. (2007). Mixed brain pathologies account for most dementia cases in community-dwelling older persons. Neurology.

[b0245] Seiler S., Pirpamer L., Hofer E., Duering M., Jouvent E., Fazekas F., Mangin J.F., Chabriat H., Dichgans M., Ropele S., Schmidt R. (2014). Magnetization transfer ratio relates to cognitive impairment in normal elderly. Front. Aging Neurosci..

[b0250] Smith S.M., Jenkinson M., Johansen-Berg H., Rueckert D., Nichols T.E., Mackay C.E., Watkins K.E., Ciccarelli O., Cader M.Z., Matthews P.M., Behrens T.E.J. (2006). Tract-based spatial statistics: Voxelwise analysis of multi-subject diffusion data. Neuroimage.

[b0255] Smith E.E., Markus H.S. (2020). New Treatment Approaches to Modify the Course of Cerebral Small Vessel Diseases. Stroke.

[b0260] Song S.-K., Sun S.-W., Ramsbottom M.J., Chang C., Russell J., Cross A.H. (2002). Dysmyelination revealed through MRI as increased radial (but unchanged axial) diffusion of water. Neuroimage.

[b0265] Therneau, T.M., T. Lumley, 2015. Package ‘survival.’ R Top. Doc.

[b0270] Toledo J.B., Arnold S.E., Raible K., Brettschneider J., Xie S.X., Grossman M., Monsell S.E., Kukull W.A., Trojanowski J.Q. (2013). Contribution of cerebrovascular disease in autopsy confirmed neurodegenerative disease cases in the National Alzheimer’s Coordinating Centre. Brain.

[b0275] Tombaugh T. (2004). Trail Making Test A and B: Normative data stratified by age and education. Arch. Clin. Neuropsychol..

[b0280] Tournier, J.D., Calamante, F., Connelly, A., 2012. MRtrix: Diffusion tractography in crossing fiber regions. Int. J. Imaging Syst. Technol. Doi: 10.1002/ima.22005.

[b0285] Tu M.-C., Lo C.-P., Huang C.-F., Hsu Y.-H., Huang W.-H., Deng J.F., Lee Y.-C., Chao L. (2017). Effectiveness of diffusion tensor imaging in differentiating early-stage subcortical ischemic vascular disease, Alzheimer’s disease and normal ageing. PLoS One.

[b0290] Tuladhar A.M., Van Norden A.G.W., De Laat K.F., Zwiers M.P., Van Dijk E.J., Norris D.G., De Leeuw F.E. (2015). White matter integrity in small vessel disease is related to cognition. NeuroImage Clin..

[b0295] Tuladhar A.M., van Dijk E., Zwiers M.P., van Norden A.G.W., de Laat K.F., Shumskaya E., Norris D.G., de Leeuw F.-E. (2016). Structural network connectivity and cognition in cerebral small vessel disease. Hum. Brain Mapp..

[b0300] Tuladhar A.M., Van Uden I.W.M., Rutten-Jacobs L.C.A., Lawrence A., Van Der Holst H., Van Norden A., De Laat K., Van Dijk E., Claassen J.A.H.R., Kessels R.P.C., Markus H.S., Norris D.G., De Leeuw F.E. (2016). Structural network efficiency predicts conversion to dementia. Neurology.

[b0305] van den Brink, H., Doubal, F.N., Duering, M., 2022. Advanced MRI in cerebral small vessel disease. Int. J. Stroke 00, 174749302210918. Doi: 10.1177/17474930221091879. 10.1177/17474930221091879 PMC9806457 35311609

[b0310] van Leijsen E.M.C., van Uden I.W.M., Bergkamp M.I., van der Holst H.M., Norris D.G., Claassen J.A.H.R., Kessels R.P.C., de Leeuw F.-E., Tuladhar A.M. (2019). Longitudinal changes in rich club organization and cognition in cerebral small vessel disease. NeuroImage Clin.

[b0315] van Norden A.G.W., de Laat K.F., Gons R.A.R., van Uden I.W.M., van Dijk E.J., van Oudheusden L.J.B., Esselink R.A.J., Bloem B.R., van Engelen B.G.M., Zwarts M.J., Tendolkar I., Olde-Rikkert M.G., van der Vlugt M.J., Zwiers M.P., Norris D.G., de Leeuw F.E. (2011). Causes and consequences of cerebral small vessel disease. The RUN DMC study: A prospective cohort study. Study rationale and protocol. BMC Neurol..

[b0320] van Uden I.W.M., van der Holst H.M., Schaapsmeerders P., Tuladhar A.M., van Norden A.G.W., de Laat K.F., Norris D.G., Claassen J.A.H.R., van Dijk E.J., Richard E., Kessels R.P.C., de Leeuw F.E. (2015). Baseline white matter microstructural integrity is not related to cognitive decline after 5years: The RUN DMC study. BBA Clin..

[b0325] van Uden I.W.M., Tuladhar A.M., van der Holst H.M., van Leijsen E.M.C., van Norden A.G.W., de Laat K.F., Rutten-Jacobs L.C.A., Norris D.G., Claassen J.A.H.R., van Dijk E.J., Kessels R.P.C., de Leeuw F.-E. (2016). Diffusion tensor imaging of the hippocampus predicts the risk of dementia; the RUN DMC study. Hum. Brain Mapp..

[b0330] Wardlaw J.M., Smith C., Dichgans M. (2019). Small vessel disease: mechanisms and clinical implications. Lancet Neurol..

[b0335] Wheeler, R.E., 2010. Permutation Tests for Linear Models in R. R Doc.

[b0340] Williams O.A., Zeestraten E.A., Benjamin P., Lambert C., Lawrence A.J., Mackinnon A.D., Morris R.G., Markus H.S., Charlton R.A., Barrick T.R. (2017). Diffusion tensor image segmentation of the cerebrum provides a single measure of cerebral small vessel disease severity related to cognitive change. NeuroImage Clin..

[b0345] Williams O.A., Zeestraten E.A., Benjamin P., Lambert C., Lawrence A.J., Mackinnon A.D., Morris R.G., Markus H.S., Barrick T.R., Charlton R.A. (2019). Predicting Dementia in Cerebral Small Vessel Disease Using an Automatic Diffusion Tensor Image Segmentation Technique. Stroke.

[b0350] Winklewski P.J., Sabisz A., Naumczyk P., Jodzio K., Szurowska E., Szarmach A. (2018). Understanding the physiopathology behind axial and radial diffusivity changes-what do we Know?. Front. Neurol..

[b0355] Wu A., Sharrett A.R., Gottesman R.F., Power M.C., Mosley T.H., Jack C.R., Knopman D.S., Windham B.G., Gross A.L., Coresh J. (2019). Association of brain magnetic resonance imaging signs with cognitive outcomes in persons with nonimpaired cognition and mild Cognitive Impairment. JAMA Netw Open.

[b0360] Zeestraten E.A., Benjamin P., Lambert C., Lawrence A.J., Williams O.A., Morris R.G., Barrick T.R., Markus H.S., Boltze J. (2016). Application of diffusion tensor imaging parameters to detect change in longitudinal studies in cerebral small vessel disease. PLoS One.

[b0365] Zeestraten E.A., Lawrence A.J., Lambert C., Benjamin P., Brookes R.L., Mackinnon A.D., Morris R.G., Barrick T.R., Markus H.S. (2017). Change in multimodal MRI markers predicts dementia risk in cerebral small vessel disease. Neurology.

[b0370] Zwiers M.P. (2010). Patching cardiac and head motion artefacts in diffusion-weighted images. Neuroimage.

