# Supplementary material for: Determining the OPTIMAL DTI analysis method for application in cerebral small vessel disease
Source: Neuroimage Clin. 2022 Jul 13;35:103114. doi: 10.1016/j.nicl.2022.103114 (PMC9421487; doi:10.1016/j.nicl.2022.103114)
Supplement: Supplementary data 1 [file mmc1.docx]

**SUPPLEMENTARY MATERIAL**

**eTable 1. MRI acquisition parameters for SCANS, RUN DMC, HARMONISATION, ASPS-Fam and CADASIL**

|  |  | **SCANS** | **RUN DMC** | **HARMONISATION** | **ASPS-FAM** | **CADASIL** |
| --- | --- | --- | --- | --- | --- | --- |
| **Sequence** |  |  |  |  |  |  |
| T1 | TR [ms] | 11.5 | 22.50 | 23.00 | 1900 | 22 |
|  | TE [ms] | 5 | 3.68 | 1.9 | 2.19 | 6 |
|  | Slice [mm] | 1.1 | 1 | 1 | 1 | 1.2 |
| FLAIR | TR [ms] | 9000 | 9000 | 9000 | 10000 | 8402 |
|  | TE [ms] | 130 | 84 | 82 | 69 | 151 |
|  | Slice [mm] | 5 | 5 | 3 | 3 | 5 |
| DTI | TR [ms] | 15600 | 10200 | 6800 | 4900 | 8300 |
|  | TE [ms] | 93.4 | 95 | 85 | 81 | 96 |
|  | Slice [mm] | 2.5 | 2.5 | 3 | 3 | 5 |
|  | b-value [s/mm^2^] | 1000 | 900 | 1150 | 1000 | 1000 |
|  | Directions | 25 | 61 | 61 | 12 | 41 |

**eTable 2. Test scores used for computing Global Cognition or the trail-making test score (TMT-B)**

| Cohort | Cognitive Index | Task Name | Measure Description |
| --- | --- | --- | --- |
| SCANS | Global Cognition | TMT-B ^1^ | Trail-making Test-B: alternating letters and numbers as quickly as possible while still maintaining accuracy |
|  |  | SL-Verbal Fluency ^2^ | Timed generation of words beginning with letter: FAS/ BHR |
|  |  | mWCST ^3^ | Card Sorting Test involving flexible shifting from learned dimensions |
|  |  | BMIPB SOIP ^4^ | Speeded cancellation of second highest of five two-digit numbers |
|  |  | Digit Symbol ^5^ | Speeded transcoding task |
|  |  | Grooved Pegboard ^4^ | Pick-up, rotation and placement of small pegs. |
|  |  | Digit Span task ^6^ | Immediate recall of digit strings (forwards & backwards) |
|  |  | Logical Memory ^6^ | Immediate and delayed recall of short stories |
|  |  | Visual Reproduction ^6^ | Immediate and delayed reproduction of line drawings |
| RUN DMC | Global Cognition | MST ^7^ | 1-letter Paper-and-Pencil Memory Scanning task: Reaction-time task on detecting memorised letters |
|  |  | DSST ^8^ | Letter–Digit Substitution Task involving match letters to numbers according to a key |
|  |  | RAVLT ^9^ | Rey Auditory Verbal Learning Test involving verbal memory |
|  |  | ROCF ^9,10^ | Rey Complex Figure Task involves reproducing a complicated line drawing, first by copying it freehand (recognition), and then drawing from memory (recall) |
|  |  | Stroop ^11,12^ | Stroop Color Word Test (short form) |
|  |  | VF ^13^ | Verbal fluency about naming animals and professions |
|  |  | VSAT ^14^ | Verbal Series Attention Test include forward and reverse generation of arithmetic series, days of the week, and months of the year; number-letter sequencing; and auditory vigilance for a spoken target letter |
| PRESERVE | Global Cognition | TMT-A ^1^ | Trail-making Test–A: connecting a set of 25 dots as quickly as possible while still maintaining accuracy |
|  |  | TMT-B ^1^ | Trail-making Test-B: alternating letters and numbers as quickly as possible while still maintaining accuracy |
|  |  | WAIS-III ^6^ | Wechsler Adult Intelligence Coding test involving coding numbers with characters according to a key |
|  |  | FAS ^2^ | Verbal fluency Letter subtask involving naming letters as soon as possible  Verbal fluency Animals subtask involving naming animals as soon as possible |
|  |  | RAVLT ^9^ | Rey Auditory Verbal Learning Test involving verbal memory |
| HARMONISATION | Global Cognition | FAB ^15^ | Frontal Assessment Battery testing executive function |
|  |  | Maze Task ^16^ | Draw around the maze, keeping the pen tip within the maze |
|  |  | Digit span task ^6^ | Participant repeats numbers in the same order and later in the reverse order as read aloud by the examiner |
|  |  | Visual memory span task ^6^ | Patient is asked to redraw a list of stimuli presented to him |
|  |  | Auditory detection task ^17^ | Patients are asked to respond as quickly as possible to presented auditory signals |
|  |  | BNT ^18^ | Boston Naming Test. A test of confrontation naming where patients are asked to name objects presented visually as two dimensional line drawings in a booklet. |
|  |  | VF ^19^ | Verbal fluency task. Assesses spontaneous verbal production. Patients are asked to come up with as many words as possible about a predefined category in a fixed period of time |
|  |  | SDMT ^20^ | Symbol Digit Modality Test. patients are presented with rows of digits and are asked to substitute the corresponding from a key provided above |
|  |  | Digit Cancellation task ^21^ | The subject receives one of more digits he must cross out from a presented list of values |
|  |  | WMS-R Visual ^22^ | Wechsler Memory Scale—Revised (WMS-R) Visual Reproduction Copy task |
|  |  | Clock Drawing task ^23^ | Patient is asked to draw a clock |
|  |  | WAIS-R Block task ^24^ | The patient is asked to replicate a pattern of blocks that the test examiner presents to them |
|  |  | Word List Recall task ^25^ | List of 10 words is presented and immediate recall, delayed recall and delayed recognition is assessed |
|  |  | Story Recall task ^17^ | The subject is asked to recall details of a story that is read to him |
|  |  | Picture Recall task ^6^ | The subject is asked to recall details of one picture among a list of pictures that are shown to him |
| ASPS-Fam | Global Cognition | G-Factor ^26^ | A principal component measure involving figural and verbal memory of the Lern und Gedaechnis Test, Trail-making Test-B, Digit Span backward, Complex reaction time task and Purdue Pegboard Test |
| CADASIL | Executive function | TMT-B ^27^ | Trail-making Test-B: alternating letters and numbers as quickly as possible while still maintaining accuracy |

**References**

1. Reitan RM. The relation of the Trail Making Test to organic brain damage. *J. Consult. Psychol.* 1955;

2. Delis DC, Kaplan E, Kramer JH. Delis-Kaplan executive function scale. . *San Antonio, TX Psychol. Corp.* 2001;

3. Nagahama Y, Okina T, Suzuki N, Matsuzaki S, Yamauchi H, Nabatame H, Matsuda M. Factor structure of a modified version of the Wisconsin Card Sorting Test: An analysis of executive deficit in Alzheimer’s disease and mild cognitive impairment. *Dement. Geriatr. Cogn. Disord.* 2003;

4. Lawrence AJ, Brookes RL, Zeestraten EA, Barrick TR, Morris RG, Markus HS. Pattern and rate of cognitive decline in cerebral small vessel disease: A prospective study. *PLoS One*. 2015;10:1–15.

5. Dumont R, Willis JO, Veizel K, Zibulsky J. Wechsler Adult Intelligence Scale-Fourth Edition. In: Encyclopedia of Special Education. 2014.

6. Wechsler D. Wechsler adult intelligence scale - Third Edition (WAIS-III). *San Antonio*. 1997;

7. Brand N. Information processing in depression and anxiety. *Psychol. Med.* 1987;

8. Van Der Elst W, Van Boxtel M, Van Breukelen G, Jolles J. The Letter Digit Substitution Test: Normative data for 1,858 healthy participants aged 24-81 from the Maastricht Aging Study (MAAS): Influence of age, education, and sex. *J. Clin. Exp. Neuropsychol.* 2006;

9. Rey A. L’examen psychologique dans les cas d’encéphalopathie traumatique. *Arch. Psychol. (Geneve).* 1941;

10. Osterrieth PA. Le test de copie d’une figure complexe; contribution à l’étude de la perception et de la mémoire. *Arch. Psychol. (Geneve).* 1944;

11. Jensen AR, Rohwer WD. The stroop color-word test: A review. *Acta Psychol. (Amst).* 1966;

12. Stroop JR. Studies of interference in serial verbal reactions. *J. Exp. Psychol.* 1935;

13. Van Der Elst W, Van Boxtel MPJ, Van Breukelen GJP, Jolles J. Normative data for the Animal, Profession and Letter M Naming verbal fluency tests for Dutch speaking participants and the effects of age, education, and sex. *J. Int. Neuropsychol. Soc.* 2006;

14. R.K. M, N. C. Verbal Series Attention Test: Clinical utility in the assessment of dementia. *Clin. Neuropsychol.* 1996;

15. Dubois B, Slachevsky A, Litvan I, Pillon B. The FAB: A frontal assessment battery at bedside. *Neurology*. 2000;

16. Worcester DA. The Porteus Maze Test and Intelligence. *Psychol. Bull.* 1951;

17. Van Veluw SJ, Hilal S, Kuijf HJ, Ikram MK, Xin X, Yeow TB, Venketasubramanian N, Biessels GJ, Chen C. Cortical microinfarcts on 3T MRI: Clinical correlates in memory-clinic patients. *Alzheimer’s Dement.* 2015;11:1500–1509.

18. Mack WJ, Freed DM, Williams BW, Henderson VW. Boston Naming Test: Shortened versions for use in Alzheimer’s disease. *Journals Gerontol.* 1992;

19. Isaacs B, Kennie AT. The set test as an aid to the detection of dementia in old people. *Br. J. Psychiatry*. 1973;

20. Smith a. Symbol Digit Modalities Test (SDMT). *Neuropsychol. Assess.* 2004;

21. Diller L, Ben Yishay Y, Gerstman LJ. Studies in Cognition and Rehabilitation in Hemiplegia. *NEW YORK UNIV.MED.CENT.* 1974;

22. Wechsler D. Wechsler memory scale - Third edition administration and scoring manual. *San Antonio, TX Psychol. Corp.* 1997;

23. Sunderland T, Hill JL, Mellow AM, Lawlor BA, Gundersheimer J, Newhouse PA, Grafman JH. Clock Drawing in Alzheimer’s Disease: A Novel Measure of Dementia Severity. *J. Am. Geriatr. Soc.* 1989;

24. Wechsler D. Wechsler Adult Intelligence Scale (WAIS-3R). *Psychol. Corp.* 1997;

25. Sahadevan S, Lim PPJ, Tan NJL, Chan SP. Diagnostic performance of two mental status tests in the older Chinese: Influence of education and age on cut-off values. *Int. J. Geriatr. Psychiatry*. 2000;

26. Amin Al Olama A, Wason JMS, Tuladhar AM, van Leijsen EMC, Koini M, Hofer E, Morris RG, Schmidt R, de Leeuw FE, Markus HS. Simple MRI score aids prediction of dementia in cerebral small vessel disease. *Neurology*. 2020;94:e1294–e1302.

27. Tombaugh TN. Trail Making Test A and B: Normative data stratified by age and education. *Arch. Clin. Neuropsychol.* 2004;

**eTable 3. MRI acquisition in the multicenter study PRESERVE**

| **Axial DTI** | **Site 1** | **Site 2** | **Site 3** | **Site 4** | **Site 5** |
| --- | --- | --- | --- | --- | --- |
| 3 T Scanner(s) | Phillips Achieva TX | Phillips Achieva, Phillips Archieva TX | Phillips Achieva TX | Phillips Ingenia | Siemens Verio Siemens Magnetom Prismafit |
| TR | 6850ms | 6850ms | 6850ms | 9100ms | 11500ms |
| TE | 75ms | 75ms | 75ms | 82ms | 93ms |
| In-plane FOV | 224x224mm2 | 224x224mm2 | 224x224mm2 | 224x224ms2 | 192x192mm2 |
| No slices | 60 | 60 | 60 | 60 | 75 |
| Nob0 | 8 | 8 | 8 | 8 | 2 |
| Max Gradient Strength | 80mT/m | 80mT/m | 80mT/m | 45mT/m | 45/80mT/m |
| Parallel imaging factor | 3 | 3 | 3 | 3 | 2 |
| No headcoil channels | 8 | 8 | 8 | 15 | 32 |
| Number of diffusion gradient directions  (b= 1000 s mm ^-2^) | 32 | 32 | 32 | 32 | 32 |

**eFigure 1. Cognitive profiles between patient cohorts. In SCANS, RUN DMC, ASPS-Fam and CADASIL, the cognitive measure Global or Executive function were split into 3 tertials.** The patients’ MMSE scores were assigned to the tertials and used in the boxplot (panel A). In HARMONISATION and PRESERVE, the MOCA instead of the MMSE scores were used (panel B). In SCANS, RUN DMC, and HARMONISATION, the patients’ MMSE or MOCA scores were additionally split into patients converting vs. not converting to dementia at a later time point (panels C and D).

MMSE= Mini Mental State Examination, MOCA= Montreal Cognitive Assessment,

tertials 1, 2,3 refer to the lowest cognitive score range, medium cognitive score range and highest cognitive score range

| **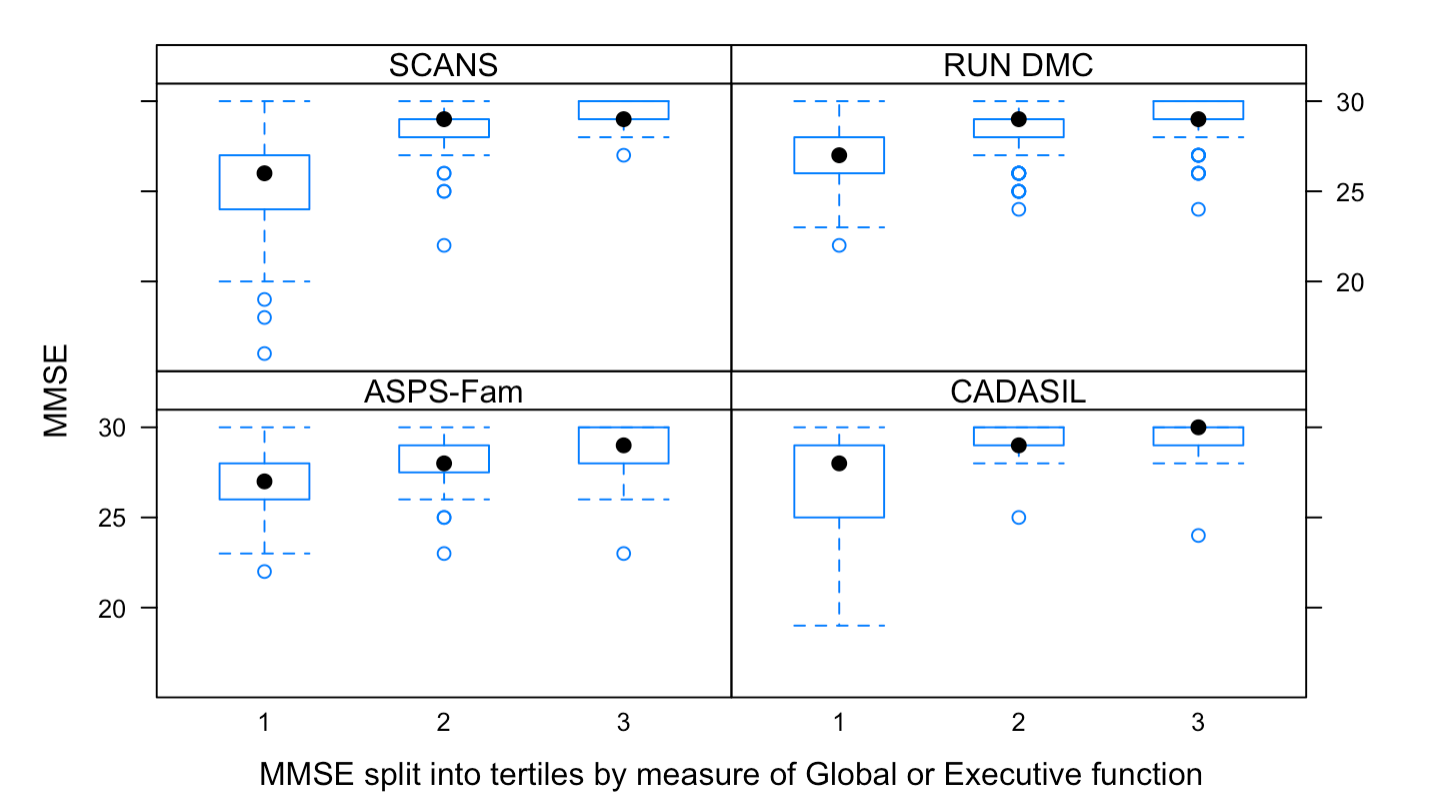A** | **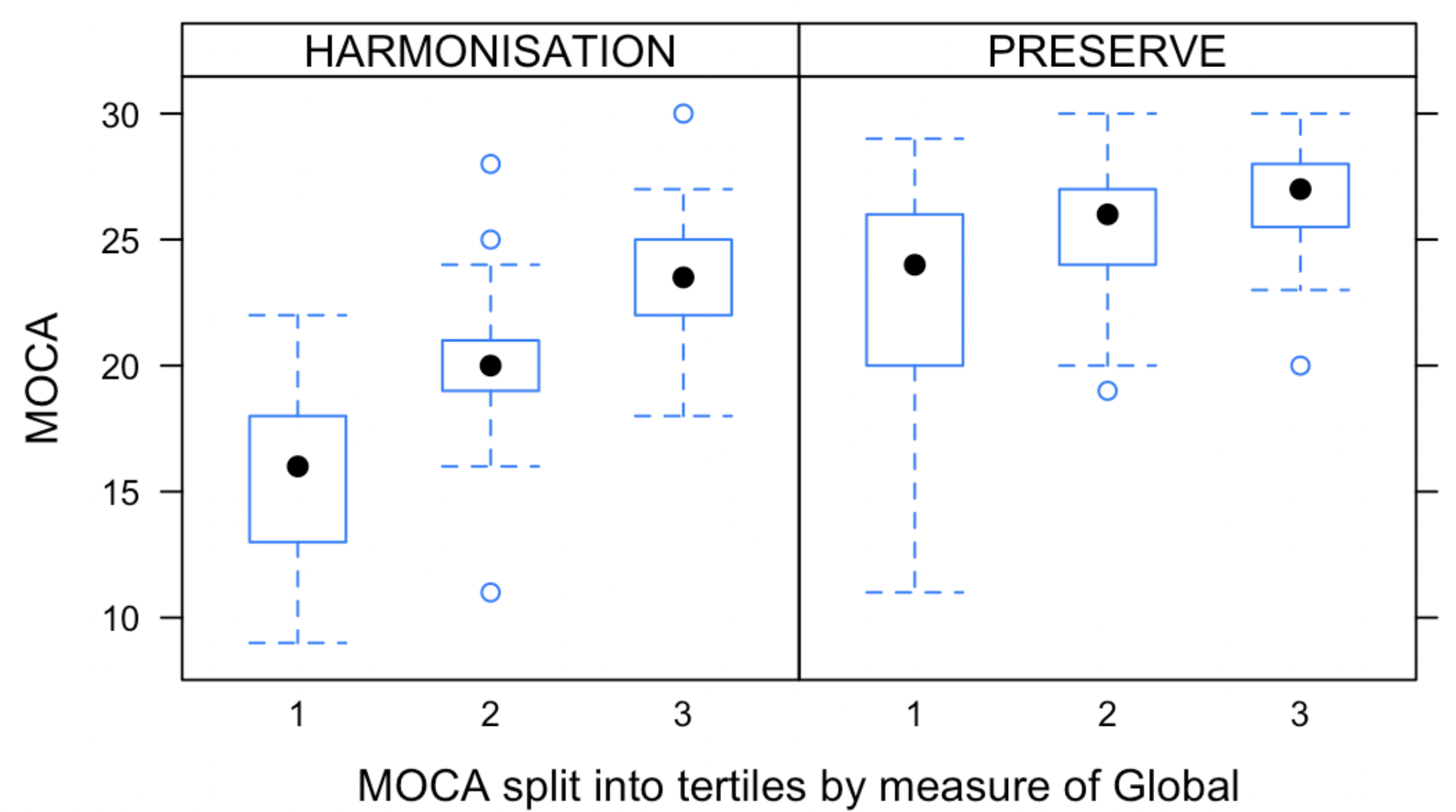B** |
| --- | --- |
| **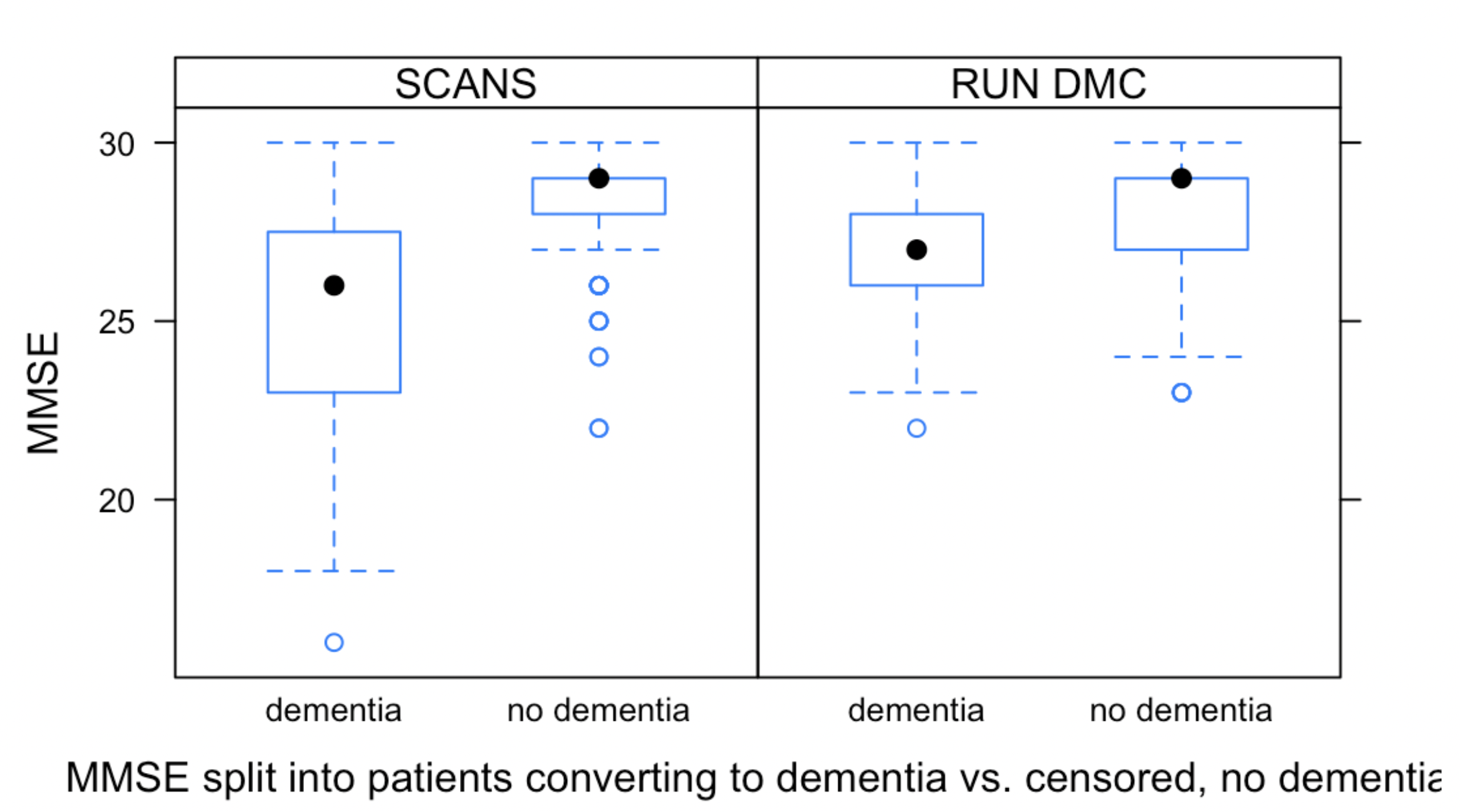C** | **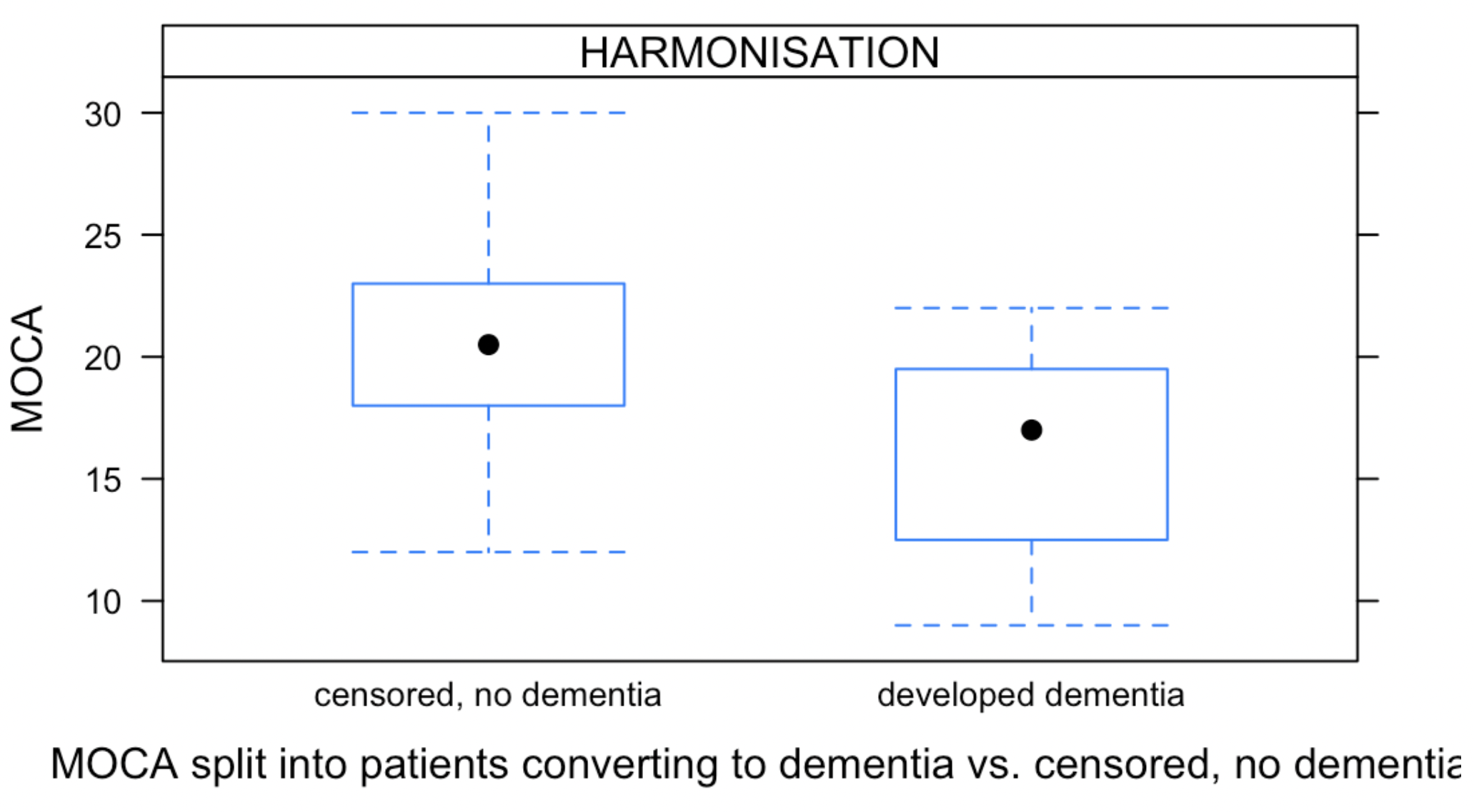D** |

**eTable 4. Clinical information of the subject's reference brain in each cohort used for DSEG** θ

DSEG θ= diffusion tensor segmentation θ, CMB= cerebral microbleeds, CADASIL= Cerebral autosomal dominant arteriopathy with subcortical infarcts and leukoencephalopathy

| **Cohorts** | **Clinical information of the individual** |
| --- | --- |
| SCANS | Age= 53, sex= male,  CMB count= 0,  lacune count= 1,  clinical diagnosis= lacunar stroke |
| RUN DMC | Age= 56, sex= female,  CMB count=0,  lacune count= 0 |
| HARMONISATION | Age=61, sex= female,  CMB count=0  lacune count=1 |
| PRESERVE | Age=83, sex= male,  CMB count= 0  lacune count=1,  clinical diagnosis= lacunar stroke |
| ASPS-Fam | Age=69, sex= female,  CMB count= 0  lacune count=0, |
| CADASIL | Age=43, sex= male  CMB count= 0  lacune count=1,  clinical diagnosis= CADASIL |

**eFigure 2**: Correlation heatmap between WM DTI histogram markers and percentage of explained variance explained by the principal components. The explained variance was lowest in RUN DMC and highest in HARMONISATION.

| **SCANS** | **RUN DMC** | **HARMONISATION** |
| --- | --- | --- |
| 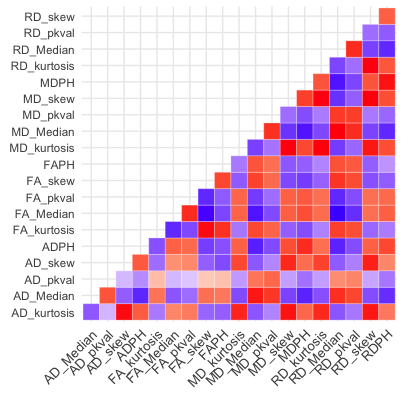 | 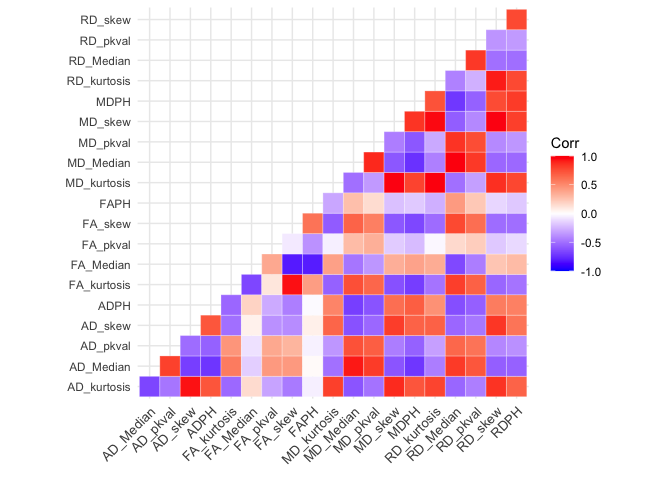 | 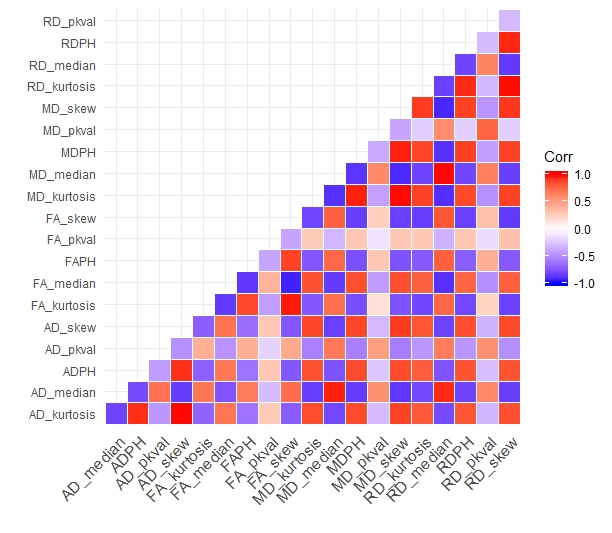 |


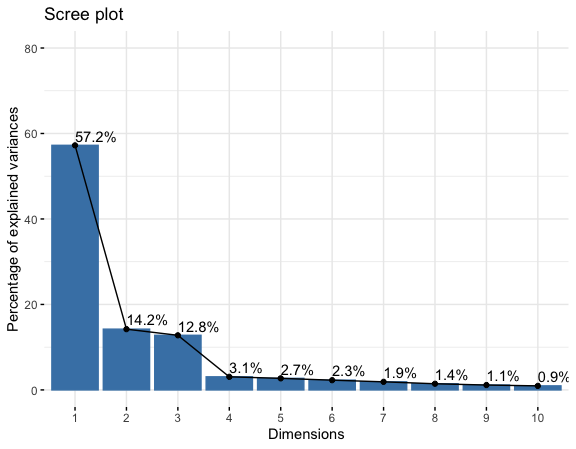

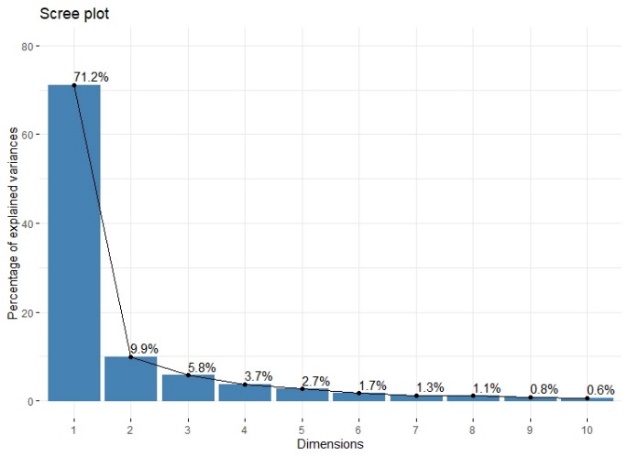

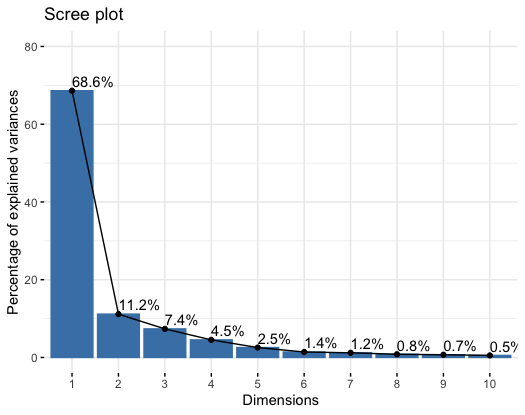


Red and blue color on the heatmap refers to a positive and negative association between 2 DTI histogram measures respectively. The strength of the association is illustrated by the marking of the color’s intensity.

| **PRESERVE** | **ASPS-Fam** | **CADASIL** |
| --- | --- | --- |
| 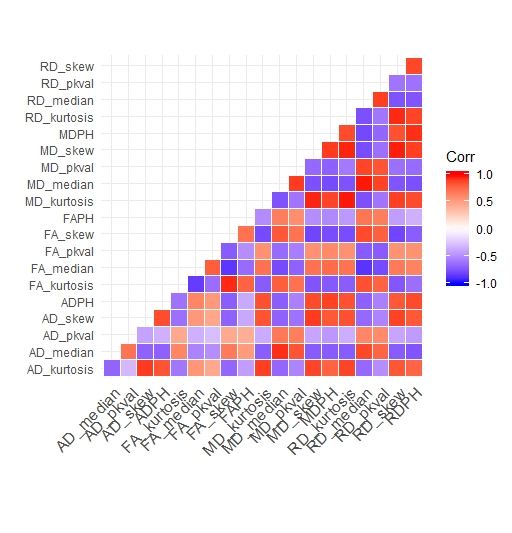 | 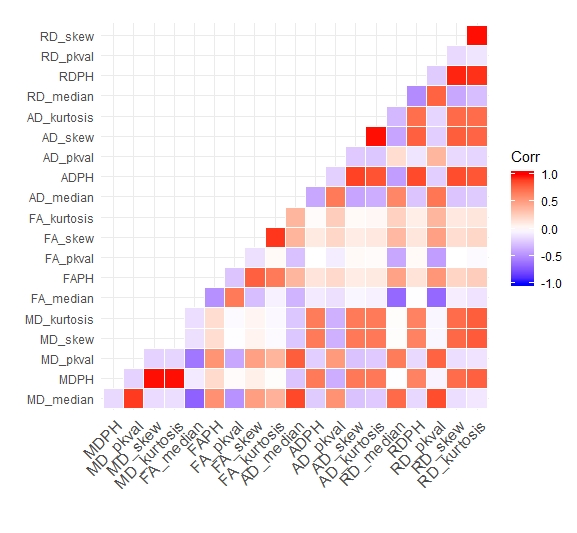 | 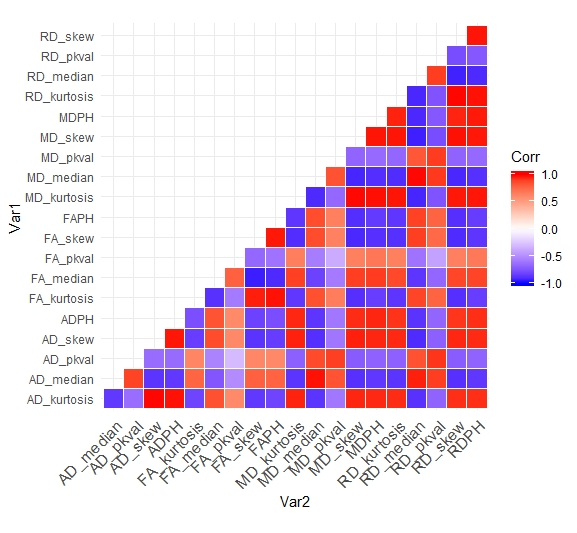 |
| 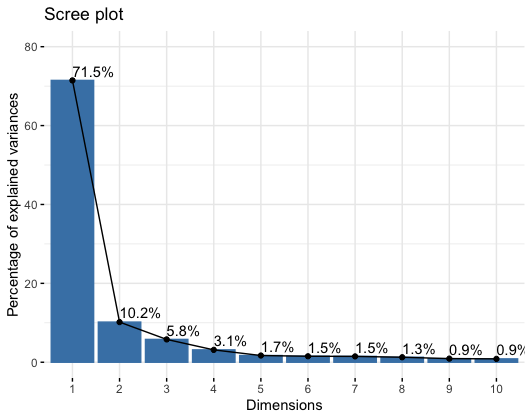 | 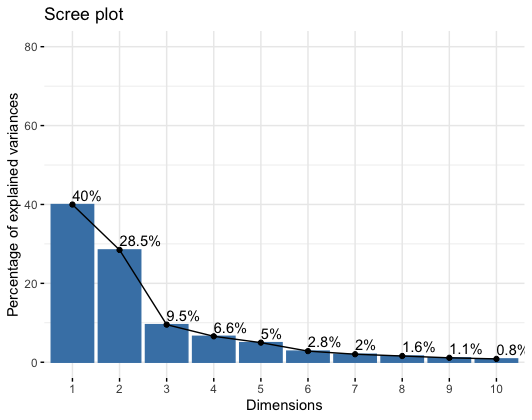 |  |


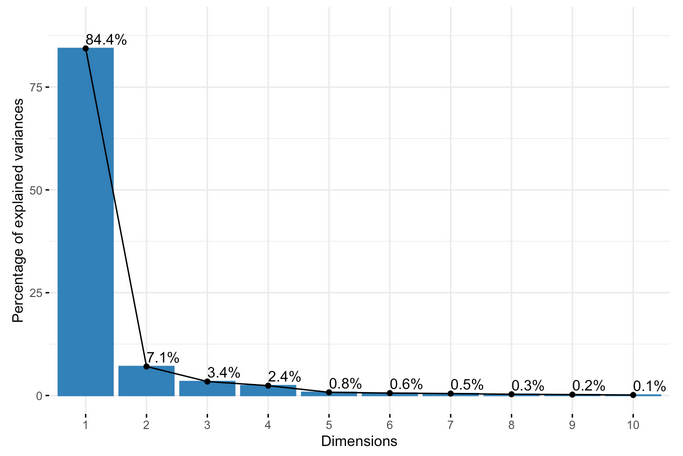
**eFigure 3** Correlation heatmap between WM DTI histogram markers and percentage of explained variance explained by the principal components. The explained variance significantly higher in the severe SVD cohort (PRESERVE) and monogenic SVD cohort (CADASIL) than in the community cohort ASPS-Fam.

Red and blue color on the heatmap refers to a positive and negative association between 2 DTI histogram measures respectively. The strength of the association is illustrated by the marking of the color’s intensity.

eTable 5a. Full linear regression model between DTI, clinical markers, i.e. age, sex and premorbid IQ, and Global Cognition in SCANS

Global= Global cognition, NART= premorbid IQ, MD median= mean diffusivity median of the WM histogram, PC1= scores of the first principal component, PSMD= peak width of skeletonized mean diffusivity, DSEG θ = diffusion tensor image segmentation θ, Geff= global efficiency network measure, β= standardized regression coefficient, 95% CI= 95% confidence interval, AIC= Akaike information criterion, P-Value= statistical value of significance with p < 0.05.

|  | **Global** | | | | | | | | | |
| --- | --- | --- | --- | --- | --- | --- | --- | --- | --- | --- |
| *Predictors* | *β*  *(95% CI)* | *P-Value* | *β*  *(95% CI)* | *P-Value* | *β*  *(95% CI)* | *P-Value* | *β*  *(95% CI)* | *P-Value* | *β*  *(95% CI)* | *P-Value* |
| NART | 0.61  (0.48- 0.75) | **<0.001** | 0.61 (0.48 – 0.74) | **<0.001** | 0.63 (0.51 – 0.76) | **<0.001** | 0.64 (0.51 – 0.76) | **<0.001** | 0.60 (0.48 – 0.73) | **<0.001** |
| Sex [male] | -0.33  (-0.62- -0.05) | **0.02** | -0.36 (-0.64 – -0.08) | **0.01** | -0.31 (-0.59 – -0.03) | **0.03** | -0.12 (-0.40 – 0.17) | 0.43 | -0.50 (-0.78 – -0.22) | **<0.001** |
| Age | -0.12  (-0.26- 0.02) | 0.08 | -0.11 (-0.24 – 0.03) | 0.12 | -0.13 (-0.26 – 0.01) | 0.07 | 0.07 (-0.09 – 0.23) | 0.40 | -0.11 (-0.24 – 0.02) | 0.11 |
| MD median | -0.25  (-0.38- -0.12) | **<0.001** |  |  |  |  |  |  |  |  |
| PC1 |  |  | -0.30 (-0.43 – -0.18) | **<0.001** |  |  |  |  |  |  |
| PSMD |  |  |  |  | -0.30 (-0.42 – -0.17) | **<0.001** |  |  |  |  |
| DSEG θ |  |  |  |  |  |  | -0.38 (-0.53 – -0.23) | **<0.001** |  |  |
| Geff |  |  |  |  |  |  |  |  | 0.35 (0.22 – 0.48) | **<0.001** |
| R^2^ / R^2^ adjusted | 0.535 / 0.518 | | 0.562 / 0.546 | | 0.561 / 0.545 | | 0.572 / 0.556 | | 0.585 / 0.569 | |
| AIC | 245.19 | | 238.33 | | 238.67 | | 235.75 | | 232.40 | |

eTable 5b. Full linear regression model between DTI, clinical markers, i.e. age, sex and education, and Global Cognition in RUN DMC

Global= Global cognition, Educ= education, MD median= mean diffusivity median of the WM histogram, PC1= scores of the first principal component, PSMD= peak width of skeletonized mean diffusivity, DSEG θ = diffusion tensor image segmentation θ, Geff= global efficiency network measure, β= standardized regression coefficient, 95% CI= 95% confidence interval, AIC= Akaike information criterion, P-Value= statistical value of significance with p < 0.05.

|  | **Global** | | | | | | | | | |
| --- | --- | --- | --- | --- | --- | --- | --- | --- | --- | --- |
| *Predictors* | *β*  *(95% CI)* | *P-Value* | *β*  *(95% CI)* | *P-Value* | *β*  *(95% CI)* | *P-Value* | *β*  *(95% CI)* | *P-Value* | *β*  *(95% CI)* | *P-Value* |
| (Intercept) | 0.08 (-0.03 – 0.18) | 0.15 | 0.03 (-0.08 – 0.13) | 0.59 | 0.02 (-0.09 – 0.12) | 0.75 | 0.02 (-0.09 – 0.14) | 0.66 | 0.05 (-0.05 – 0.16) | 0.30 |
| Age | -0.32 (-0.41 – -0.23) | **<0.001** | -0.30 (-0.39 – -0.21) | **<0.001** | -0.31 (-0.40 – -0.23) | **<0.001** | -0.37 (-0.47 – -0.28) | **<0.001** | -0.32 (-0.41 – -0.24) | **<0.001** |
| Educ | 0.41 (0.34 – 0.48) | **<0.001** | 0.40 (0.33 – 0.47) | **<0.001** | 0.42 (0.34 – 0.49) | **<0.001** | 0.41 (0.34 – 0.49) | **<0.001** | 0.40 (0.33 – 0.48) | **<0.001** |
| Sex [Male] | -0.14 (-0.28 – 0.00) | 0.06 | -0.05 (-0.20 – 0.09) | 0.47 | -0.03 (-0.18 – 0.11) | 0.67 | -0.05 (-0.20 – 0.11) | 0.57 | -0.10 (-0.24 – 0.04) | 0.16 |
| MD Median | -0.22 (-0.31 – -0.14) | **<0.001** |  |  |  |  |  |  |  |  |
| PC1 |  |  | -0.25 (-0.34 – -0.16) | **<0.001** |  |  |  |  |  |  |
| PSMD |  |  |  |  | -0.24 (-0.33 – -0.16) | **<0.001** |  |  |  |  |
| DSEG θ |  |  |  |  |  |  | -0.12 (-0.22 – -0.02) | **0.02** |  |  |
| Geff |  |  |  |  |  |  |  |  | 0.22 (0.14 – 0.31) | **<0.001** |
| R^2^ / R^2^ adjusted | 0.455/ 0.450 | | 0.460/ 0.455 | | 0.461/ 0.456 | | 0.430/ 0.424 | | 0.456/ 0.451 | |
| AIC | 981.32 | | 977.51 | | 976.24 | | 1001.23 | | 980.45 | |

eTable 5c.Full linear regression models between DTI, clinical markers, i.e. age, sex and education, and Global Cognition in HARMONISATION

|  | **Global** | | | | | | | | | | |
| --- | --- | --- | --- | --- | --- | --- | --- | --- | --- | --- | --- |
| *Predictors* | *β*  *(95% CI)* | *P-Value* | *β*  *(95% CI)* | *P-Value* | *β*  *(95% CI)* | *P-Value* | *β*  *(95% CI)* | *P-Value* | *β*  *(95% CI)* | *P-Value* |  |
| Age | -0.15 (-0.31 – 0.00) | 0.05 | -0.15 (-0.30 – 0.00) | 0.05 | -0.18 (-0.33 – -0.04) | **0.01** | -0.01 (-0.19 – 0.17) | 0.89 | -0.26 (-0.40 – -0.11) | **<0.001** |  |
| Sex [Male] | 0.54 (0.25 – 0.84) | **<0.001** | 0.59 (0.29 – 0.89) | **<0.001** | 0.56 (0.27 – 0.85) | **<0.001** | 0.63 (0.33 – 0.92) | **<0.001** | 0.43 (0.12 – 0.73) | **0.01** |  |
| Educ | 0.45 (0.30 – 0.59) | **<0.001** | 0.43 (0.28 – 0.57) | **<0.001** | 0.42 (0.28 – 0.57) | **<0.001** | 0.46 (0.32 – 0.60) | **<0.001** | 0.43 (0.28 – 0.58) | **<0.001** |  |
| MD median | -0.31 (-0.47 – -0.15) | **<0.001** |  |  |  |  |  |  |  |  |  |
| PC1 |  |  | -0.33 (-0.49 – -0.17) | **<0.001** |  |  |  |  |  |  |  |
| PSMD |  |  |  |  | -0.33 (-0.47 – -0.18) | **<0.001** |  |  |  |  |  |
| DSEG θ |  |  |  |  |  |  | -0.44 (-0.62 – -0.25) | **<0.001** |  |  |  |
| Geff |  |  |  |  |  |  |  |  | 0.15 (0.01 – 0.30) | **0.04** |  |
| R^2^ / R^2^ adjusted | 0.435 / 0.417 | | 0.443 / 0.424 | | 0.455 / 0.437 | | 0.462 / 0.444 | | 0.388 / 0.368 | |  |
| AIC | 296.58 | | 294.92 | | 291.98 | | 290.44 | | 306.64 | |  |

Global= Global cognition, Educ= education, MD median= mean diffusivity median of the WM histogram, PC1= scores of the first principal component, PSMD= peak width of skeletonized mean diffusivity, DSEG θ = diffusion tensor image segmentation θ, Geff= global efficiency network measure, β= standardized regression coefficient, 95% CI= 95% confidence interval, AIC= Akaike information criterion, P-Value= statistical value of significance with p < 0.05.

eTable 5d. Full linear regression models between DTI, clinical markers, i.e. age, sex and premorbid IQ, and Global Cognition in PRESERVE

|  | **Global** | | | | | | | | | |
| --- | --- | --- | --- | --- | --- | --- | --- | --- | --- | --- |
| *Predictors* | *β*  *(95% CI)* | *P-Value* | *β*  *(95% CI)* | *P-Value* | *β*  *(95% CI)* | *P-Value* | *β*  *(95% CI)* | *P-Value* | *β*  *(95% CI)* | *P-Value* |
| Age | 0.31  (0.14 – 0.48) | **<0.001** | 0.32 (0.14 – 0.49) | **<0.001** | 0.32 (0.15 – 0.49) | **<0.001** | 0.24 (0.06 – 0.42) | **0.01** | 0.22 (0.03 – 0.40) | **0.02** |
| NART | 0.37  (0.20 – 0.53) | **<0.001** | 0.37 (0.20 – 0.53) | **<0.001** | 0.36 (0.20 – 0.53) | **<0.001** | 0.37 (0.19 – 0.55) | **<0.001** | 0.39 (0.21 – 0.57) | **<0.001** |
| Sex [Male] | 0.20  (-0.14 – 0.54) | 0.24 | 0.23 (-0.12 – 0.57) | 0.20 | 0.31 (-0.02 – 0.64) | 0.07 | 0.31 (-0.05 – 0.68) | 0.09 | 0.39 (0.02 – 0.76) | **0.04** |
| MD median | -0.39  (-0.57 – -0.22) | **<0.001** |  |  |  |  |  |  |  |  |
| PC1 |  |  | 0.37 (0.20 – 0.54) | **<0.001** |  |  |  |  |  |  |
| PSMD |  |  |  |  | -0.41 (-0.57 – -0.24) | **<0.001** |  |  |  |  |
| DSEG θ |  |  |  |  |  |  | -0.19 (-0.37 – -0.01) | **0.04** |  |  |
| Geff |  |  |  |  |  |  |  |  | -0.19 (-0.36 – -0.01) | **0.04** |
| R^2^ / R^2^ adjusted | 0.439 / 0.389 | | 0.426 / 0.375 | | 0.457 / 0.409 | | 0.340 / 0.282 | | 0.340 / 0.281 | |
| AIC | 241.89 | | 244.21 | | 238.73 | | 257.98 | | 258.03 | |

Global= Global cognition, NART= premorbid IQ, MD median= mean diffusivity median of the WM histogram, PC1= scores of the first principal component, PSMD= peak width of skeletonized mean diffusivity, DSEG θ = diffusion tensor image segmentation θ, Geff= global efficiency network measure, β= standardized regression coefficient, 95% CI= 95% confidence interval, AIC= Akaike information criterion, P-Value= statistical value of significance with p < 0.05.

eTable 5e. Full linear regression models between DTI, clinical markers, i.e. age, sex and education, and Global Cognition in ASPS-Fam

|  | **Global** | | | | | | | | | |
| --- | --- | --- | --- | --- | --- | --- | --- | --- | --- | --- |
| *Predictors* | *β*  *(95% CI)* | *P-Value* | *β*  *(95% CI)* | *P-Value* | *β*  *(95% CI)* | *P-Value* | *β*  *(95% CI)* | *P-Value* | *β*  *(95% CI)* | *P-Value* |
| Age | -0.47  (-0.58 – -0.37) | **<0.001** | -0.51 (-0.62 – -0.41) | **<0.001** | -0.52 (-0.63 – -0.41) | **<0.001** | -0.54 (-0.63 – -0.44) | **<0.001** | -0.48 (-0.57 – -0.38) | **<0.001** |
| Sex [Male] | 0.01 (-0.18 – 0.20) | 0.92 | 0.04 (-0.15 – 0.24) | 0.66 | 0.04 (-0.15 – 0.24) | 0.66 | 0.03 (-0.17 – 0.22) | 0.79 | -0.04 (-0.23 – 0.16) | 0.70 |
| Educ | 0.33 (0.24 – 0.43) | **<0.001** | 0.35 (0.25 – 0.45) | **<0.001** | 0.35 (0.25 – 0.45) | **<0.001** | 0.36 (0.26 – 0.45) | **<0.001** | 0.33 (0.23 – 0.43) | **<0.001** |
| MD median | -0.14  (-0.24 – -0.04) | **0.01** |  |  |  |  |  |  |  |  |
| PC1 |  |  | -0.04 (-0.14 – 0.06) | 0.38 |  |  |  |  |  |  |
| PSMD |  |  |  |  | -0.03 (-0.13 – 0.08) | 0.64 |  |  |  |  |
| DSEG θ |  |  |  |  |  |  | -0.14 (-0.23 – -0.05) | **<0.001** |  |  |
| Geff |  |  |  |  |  |  |  |  | 0.18 (0.09 – 0.28) | **<0.001** |
| R^2^ / R^2^ adjusted | 0.525 / 0.516 | | 0.511 / 0.503 | | 0.510 / 0.501 | | 0.529 / 0.521 | | 0.538 / 0.530 | |
| AIC | 488.48 | | 494.77 | | 495.32 | | 486.35 | | 482.02 | |

Global= Global cognition, Educ= education, MD median= mean diffusivity median of the WM histogram, PC1= scores of the first principal component, PSMD= peak width of skeletonized mean diffusivity, DSEG θ = diffusion tensor image segmentation θ, Geff= global efficiency network measure, β= standardized regression coefficient, 95% CI= 95% confidence interval, AIC= Akaike information criterion, P-Value= statistical value of significance with p < 0.05.

eTable 5f. Full linear regression models between DTI, clinical markers, i.e. age, sex and education, and executive function in CADASIL

|  | **TMT-B** | | | | | | | | | |
| --- | --- | --- | --- | --- | --- | --- | --- | --- | --- | --- |
| *Predictors* | *β*  *(95% CI)* | *P-Value* | *β*  *(95% CI)* | *P-Value* | *β*  *(95% CI)* | *P-Value* | *β*  *(95% CI)* | *P-Value* | *β*  *(95% CI)* | *P-Value* |
| Age | 0.10  (-0.22 – 0.41) | 0.54 | 0.14 (-0.21 – 0.48) | 0.42 | 0.27 (-0.04 – 0.58) | 0.09 | 0.08 (-0.22 – 0.38) | 0.60 | 0.03 (-0.29 – 0.35) | 0.86 |
| Sex [Male] | 0.08  (-0.61 – 0.45) | 0.76 | -0.07 (-0.61 – 0.46) | 0.79 | -0.02 (-0.50 – 0.46) | 0.93 | 0.10 (-0.41 – 0.62) | 0.69 | -0.34 (-0.95 – 0.28) | 0.27 |
| Educ | 0.24  (-0.06 – 0.55) | 0.11 | 0.25 (-0.06 – 0.56) | 0.11 | 0.23 (-0.05 – 0.50) | 0.10 | 0.24 (-0.05 – 0.54) | 0.11 | 0.23 (-0.09 – 0.55) | 0.15 |
| MD Median | -0.50  (-0.82 – -0.18) | **<0.001** |  |  |  |  |  |  |  |  |
| PC1 |  |  | 0.50 (0.15 – 0.85) | **0.01** |  |  |  |  |  |  |
| PSMD |  |  |  |  | -0.70 (-1.01 – -0.39) | **<0.001** |  |  |  |  |
| DSEG θ |  |  |  |  |  |  | -0.50 (-0.80 – -0.20) | **<0.001** |  |  |
| Geff |  |  |  |  |  |  |  |  | 0.45 (0.09 – 0.82) | **0.02** |
| R^2^ / R^2^ adjusted | 0.292 / 0.227 |  | 0.272 / 0.206 | | 0.411 / 0.357 | | 0.313 / 0.251 | | 0.243 / 0.174 | |
| AIC | 133.15 | | 134.50 | | 124.11 | | 131.65 | | 136.43 | |

Global= Global cognition, Educ= education, MD median= mean diffusivity median of the WM histogram, PC1= scores of the first principal component, PSMD= peak width of skeletonized mean diffusivity, DSEG θ = diffusion tensor image segmentation θ, Geff= global efficiency network measure, β= standardized regression coefficient, 95% CI= 95% confidence interval, AIC= Akaike information criterion, P-Value= statistical value of significance with p < 0.05.

eTable 6a. Full Cox regression models between baseline imaging marker, clinical markers and later dementia conversion in SCANS

|  | **Dementia conversion** | | | | | | | | | |
| --- | --- | --- | --- | --- | --- | --- | --- | --- | --- | --- |
| *Markers* | *HR*  *(95% CI)* | *P-Value* | *HR*  *(95% CI)* | *P-Value* | *HR*  *(95% CI)* | *P-Value* | *HR*  *(95% CI)* | *P-Value* | *HR*  *(95% CI)* | *P-Value* |
| Age | 1.73  (0.96 – 3.11) | 0.07 | 1.71 (0.94 – 3.11) | 0.08 | 1.79 (0.98 – 3.28) | 0.06 | 0.94 (0.50 – 1.77) | 0.86 | 1.68 (0.94 – 3.03) | 0.10 |
| Sex [Male] | 5.76  (1.23 – 26.85) | 0.03 | 6.11 (1.32 – 28.18) | **0.02** | 5.73 (1.26 – 25.95) | **0.02** | 2.15 (0.42 – 10.98) | 0.36 | 8.56 (1.90 – 38.66) | **0.01** |
| NART | 0.71  (0.43 – 1.16) | 0.17 | 0.64 (0.39 – 1.05) | 0.08 | 0.61 (0.37 – 1.03) | **0.06** | 0.74 (0.45 – 1.22) | 0.24 | 0.59 (0.36 – 0.97) | **0.04** |
| MD median | 2.19  (1.51 – 3.16) | **<0.001** |  |  |  |  |  |  |  |  |
| PC1 |  |  | 2.28 (1.51 – 3.44) | **<0.001** |  |  |  |  |  |  |
| PSMD |  |  |  |  | 1.74 (1.29 – 2.34) | **<0.001** |  |  |  |  |
| DSEG θ |  |  |  |  |  |  | 3.52 (2.09 – 5.92) | **<0.001** |  |  |
| Geff |  |  |  |  |  |  |  |  | 0.37 (0.23 – 0.61) | **<0.001** |
| AIC | 138.37 | | 139.60 | | 143.70 | | 128.21 | | 138.39 | |

NART= premorbid IQ, MD Median= mean diffusivity median of the WM histogram, PC1= scores of the first principal component, PSMD= peak width of skeletonized mean diffusivity, DSEG θ = diffusion tensor image segmentation θ, Geff= global efficiency network measure, AIC= Akaike information criterion, HR= hazard ratio, AIC= Akaike information criterion, 95% CI= 95% confidence interval, P-Value= statistical value of significance with p < 0.05

eTable 6b Full Cox regression model between baseline imaging marker, clinical markers and later dementia conversion in RUN DMC

|  | **Dementia conversion** | | | | | | | | | |
| --- | --- | --- | --- | --- | --- | --- | --- | --- | --- | --- |
| *Markers* | *HR*  *(95% CI)* | *P-Value* | *HR*  *(95% CI)* | *P-Value* | *HR*  *(95% CI)* | *P-Value* | *HR*  *(95% CI)* | *P-Value* | *HR*  *(95% CI)* | *P-Value* |
| Age | 3.15 (2.08 – 4.76) | **<0.001** | 2.84 (1.87 – 4.31) | **<0.001** | 3.14 (2.11 – 4.68) | **<0.001** | 3.18 (2.06 – 4.90) | **<0.001** | 2.97 (1.98 – 4.45) | **<0.001** |
| Sex [Male] | 1.15 (0.65 – 2.04) | 0.62 | 1.01 (0.57 – 1.78) | 0.98 | 0.94 (0.53 – 1.68) | 0.83 | 0.90 (0.48 – 1.69) | 0.74 | 1.03 (0.58 – 1.82) | 0.93 |
| Educ | 0.97 (0.74 – 1.27) | 0.81 | 0.98 (0.74 – 1.28) | 0.86 | 0.96 (0.73 – 1.26) | 0.75 | 0.98 (0.75 – 1.28) | 0.87 | 0.99 (0.76 – 1.30) | 0.97 |
| MD Median | 1.33 (1.00 – 1.76) | 0.05 |  |  |  |  |  |  |  |  |
| PC1 |  |  | 1.57 (1.15 – 2.14) | **<0.001** |  |  |  |  |  |  |
| PSMD |  |  |  |  | 1.45 (1.14 – 1.83) | **<0.001** |  |  |  |  |
| DSEG θ |  |  |  |  |  |  | 1.33 (0.91 – 1.93) | 0.14 |  |  |
| Geff |  |  |  |  |  |  |  |  | 0.64 (0.46 – 0.89) | **0.01** |
| AIC | 515.69 | | 511.60 | | 511.51 | | 517.17 | | 512.59 | |

Educ= education, MD Median= mean diffusivity median of the WM histogram, PC1= scores of the first principal component, PSMD= peak width of skeletonized mean diffusivity, DSEG θ = diffusion tensor image segmentation θ, Geff= global efficiency network measure, AIC= Akaike information criterion, HR= hazard ratio, AIC= Akaike information criterion, 95% CI= 95% confidence interval, P-Value= statistical value of significance with p < 0.05

eTable 6c. Full Cox regression model between baseline imaging marker, clinical markers and later dementia conversion in HARMONISATION

|  | **Dementia conversion** | | | | | | | | | |
| --- | --- | --- | --- | --- | --- | --- | --- | --- | --- | --- |
| *Markers* | *HR*  *(95% CI)* | *P-Value* | *HR*  *(95% CI)* | *P-Value* | *HR*  *(95% CI)* | *P-Value* | *HR*  *(95% CI)* | *P-Value* | *HR*  *(95% CI)* | *P-Value* |
| Age | 1.24 (0.76 – 2.05) | 0.39 | 1.25 (0.75 – 2.08) | 0.40 | 1.32 (0.82 – 2.13) | 0.25 | 1.05 (0.59 – 1.89) | 0.86 | 1.60 (0.96 – 2.68) | 0.07 |
| Sex [Male] | 0.30 (0.11 – 0.82) | **0.02** | 0.29 (0.11 – 0.82) | **0.02** | 0.29 (0.10 – 0.81) | **0.02** | 0.32 (0.12 – 0.84) | **0.02** | 0.38 (0.14 – 0.98) | **0.05** |
| Educ | 0.84 (0.53 – 1.35) | 0.48 | 0.85 (0.53 – 1.35) | 0.49 | 0.82 (0.51 – 1.31) | 0.41 | 0.79 (0.49 – 1.28) | 0.34 | 0.88 (0.57 – 1.36) | 0.57 |
| MD Median | 1.78 (1.08 – 2.93) | **0.02** |  |  |  |  |  |  |  |  |
| PC1 |  |  | 1.74 (1.03 – 2.92) | **0.04** |  |  |  |  |  |  |
| PSMD |  |  |  |  | 1.73 (1.13 – 2.65) | **0.01** |  |  |  |  |
| DSEG θ |  |  |  |  |  |  | 1.94 (1.06 – 3.53) | **0.03** |  |  |
| Geff |  |  |  |  |  |  |  |  | 0.79 (0.49 – 1.30) | 0.36 |
| AIC | 173.51 | | 174.17 | | 172.71 | | 173.67 | | 177.58 | |

Educ= education, MD Median= mean diffusivity median of the WM histogram, PC1= scores of the first principal component, PSMD= peak width of skeletonized mean diffusivity, DSEG θ = diffusion tensor image segmentation θ, Geff= global efficiency network measure, AIC= Akaike information criterion, HR= hazard ratio, AIC= Akaike information criterion, 95% CI= 95% confidence interval, P-Value= statistical value of significance with p < 0.05

| **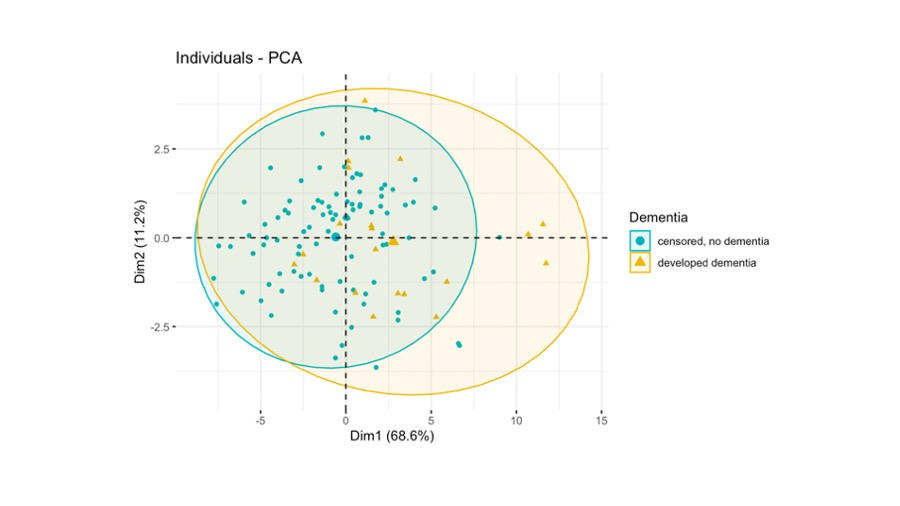**  (A) | **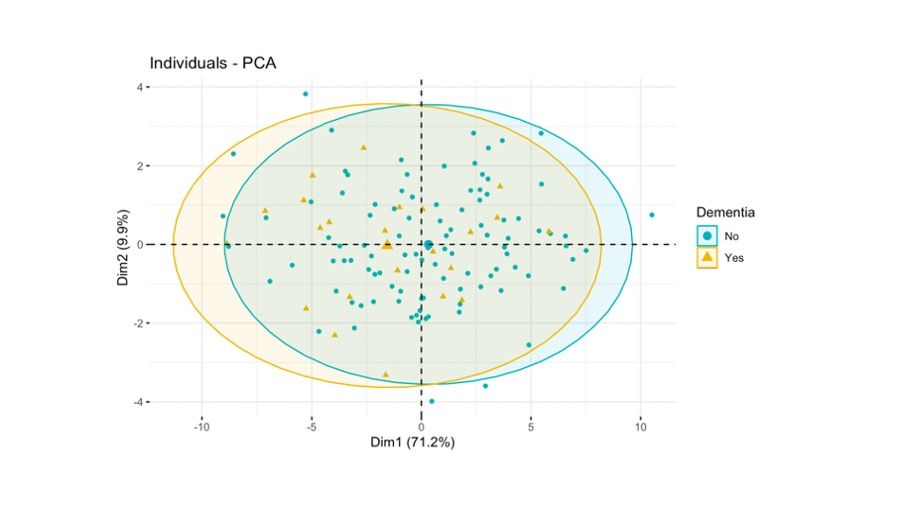**  (B)  (B)  (C) | **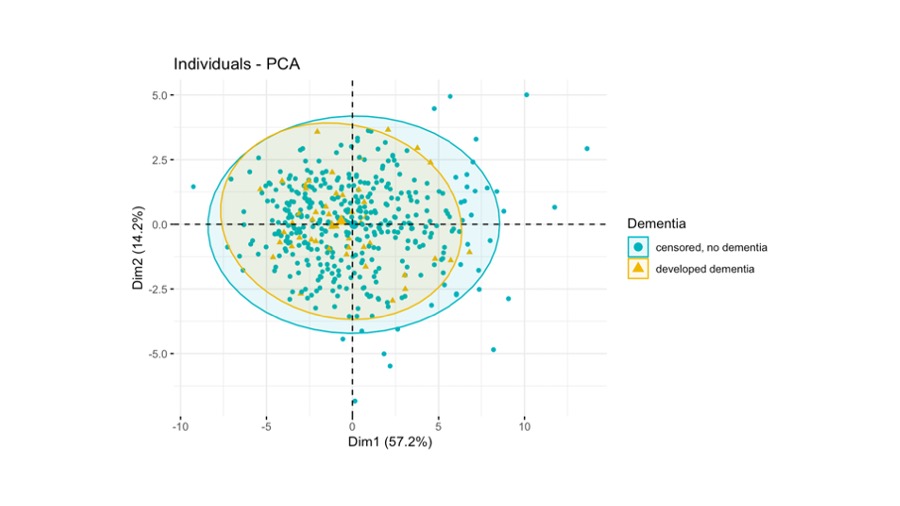**  (C) |
| --- | --- | --- |
| Panel (A), panel (B) and panel (C) show dementia cases and no dementia cases on the first and second principal component in SCANS (A), RUN DMC (B) and HARMONISATION (C). Panel (D) and panel (E) show no dementia cases and the dementia subtype cases on the first and second principal components in RUN DMC (D) and HARMONISATION (E)  Dim 1= 1st principal component, Dim 2= 2^nd^ principal component, AD= Alzheimer’s disease, VD= Vascular Dementia, AD/VD= Mixed dementia with Alzheimer’s and vascular dementia, LBD= Lewy body dementia | **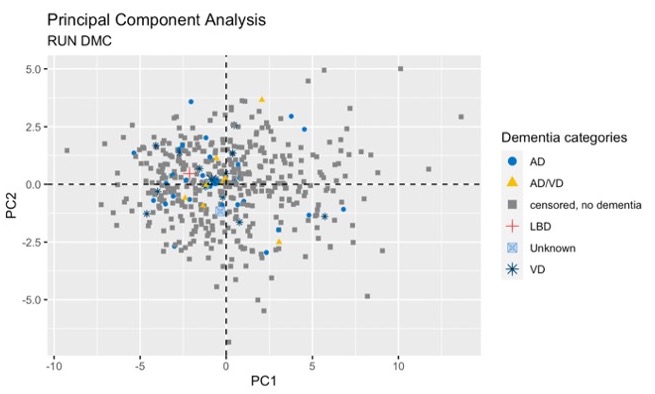**  (D) | **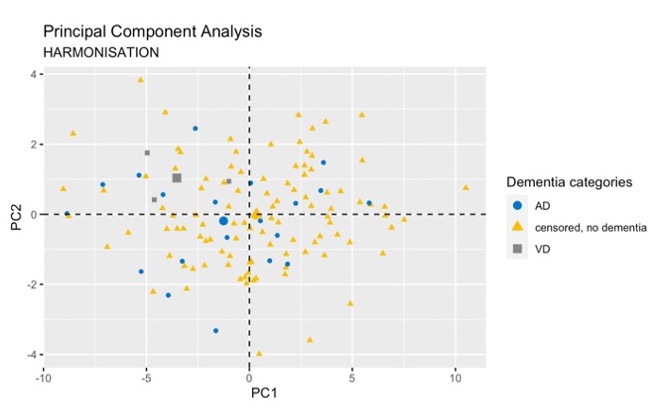**  (E) |

**eFigure 4. There were no patterns showing that dementia cases or dementia subtypes were better represented on the second instead of the first principal component dimension.**

**eFigure 5. Rates of dementia conversion in the cohorts with dementia conversion. The rate of dementia conversion was higher in HARMONISATION than in SCANS or RUN DMC**

**
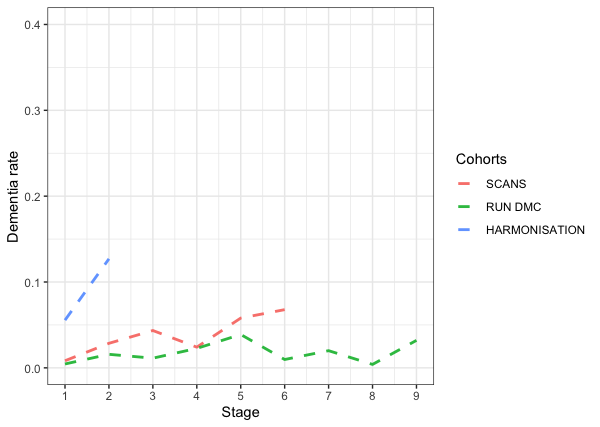
**

Stage 1= Baseline- year 1, Stage 2= year 1-2, Stage 3= year 2-3, Stage 4= year 3-4, Stage 5= year 4- 5, Stage 6= year 5-6, Stage 7= year 6-7, Stage 8= year 7-8, Stage 9= year 8-9

**eFigure 6. Differences between vascular dementia (VD) and Alzheimer’s disease (AD) over 3 years intervals in RUN DMC.** The DTI baseline measures PC1, MD median, PSMD were overall higher and Geff lower for VD than AD subtype across the different time intervals.

MD Median= mean diffusivity median of the WM histogram, PC1= scores of the first principal component, PSMD= peak width of skeletonized mean diffusivity, DSEG θ = diffusion tensor image segmentation θ, Geff= global efficiency network measure, VD= vascular dementia, AD/VD= mixed Alzheimer’s and vascular dementia, AD= Alzheimer’s disease

| **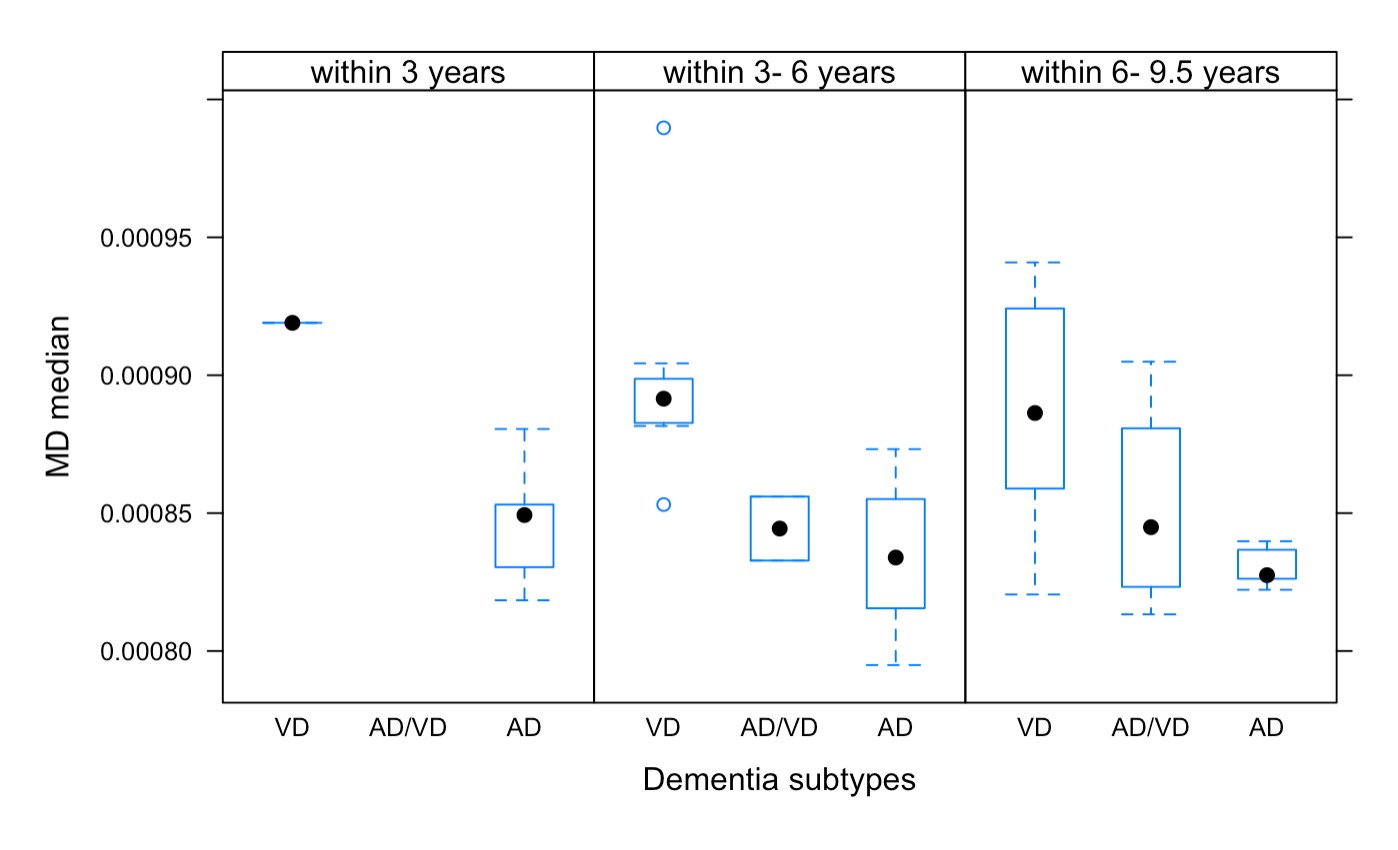**  (A) | **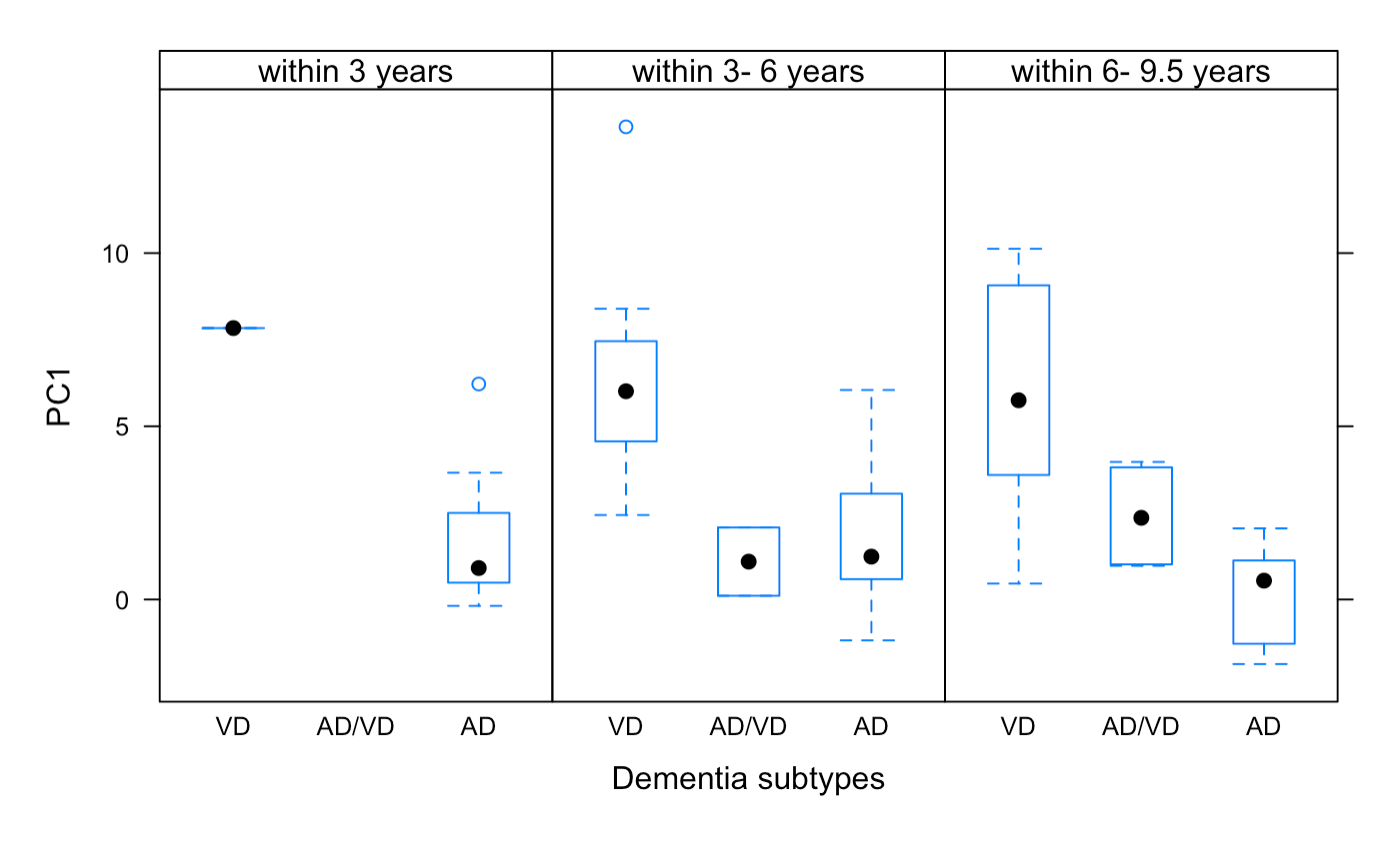**  (B) | **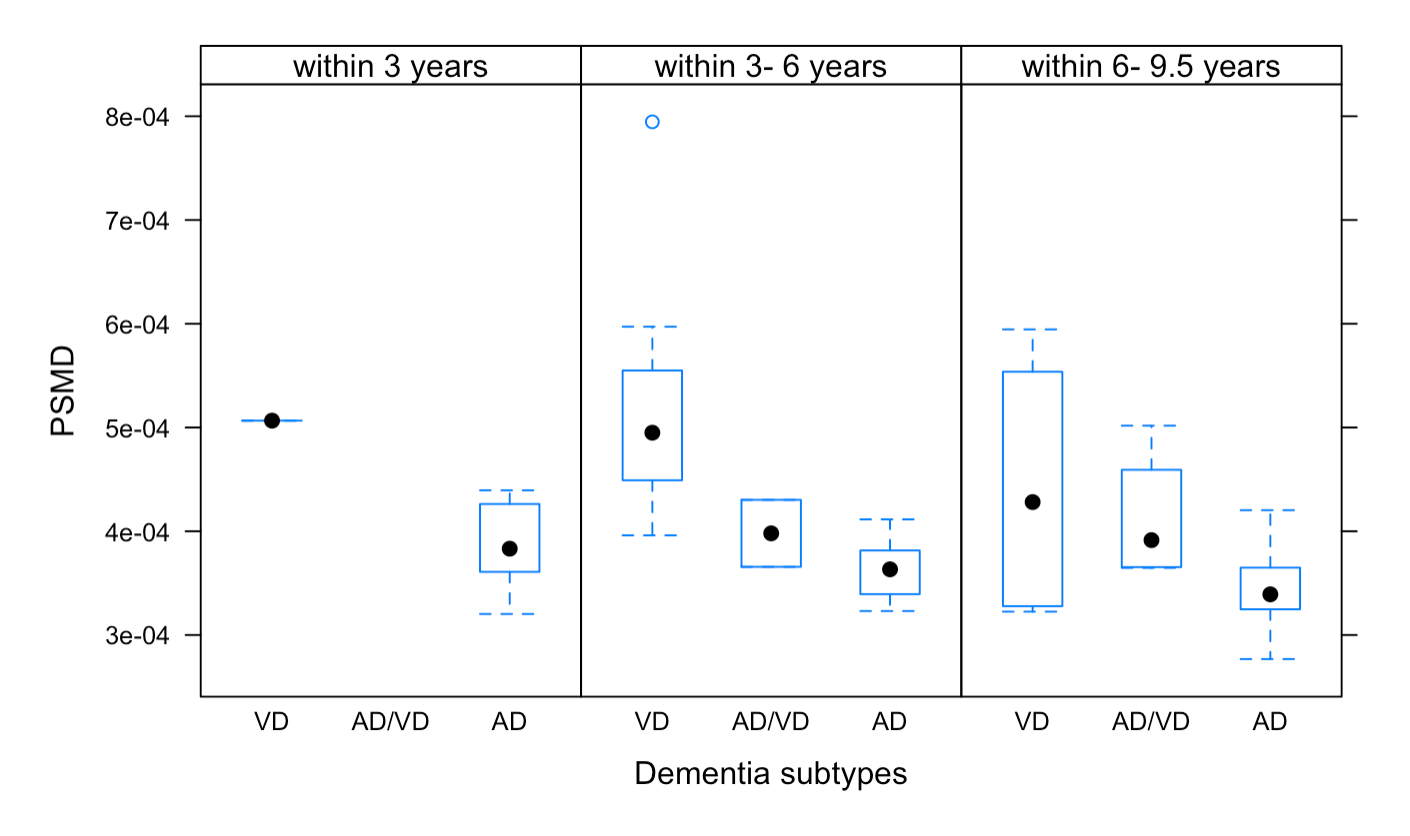**  (C) |
| --- | --- | --- |
| **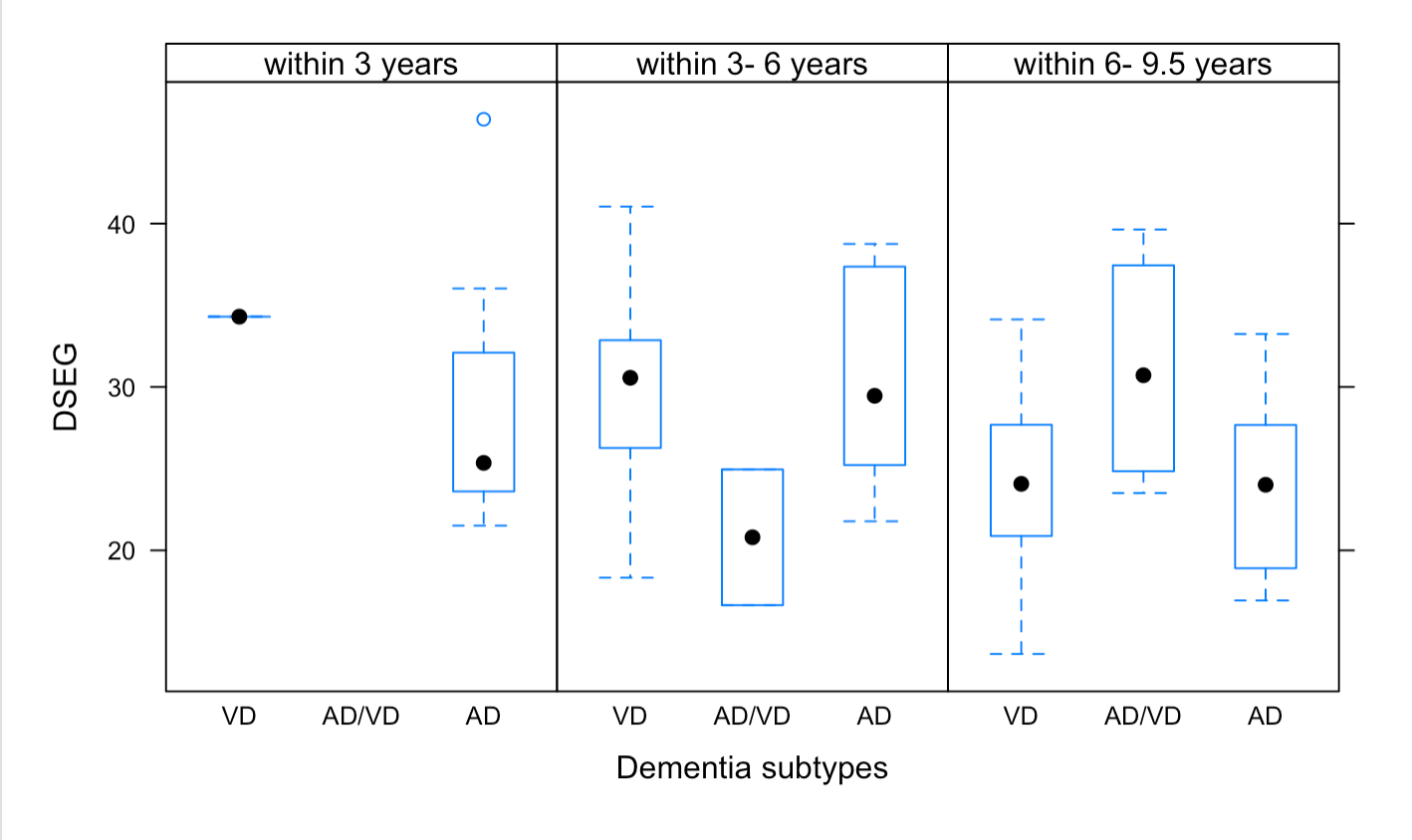**  (D) | **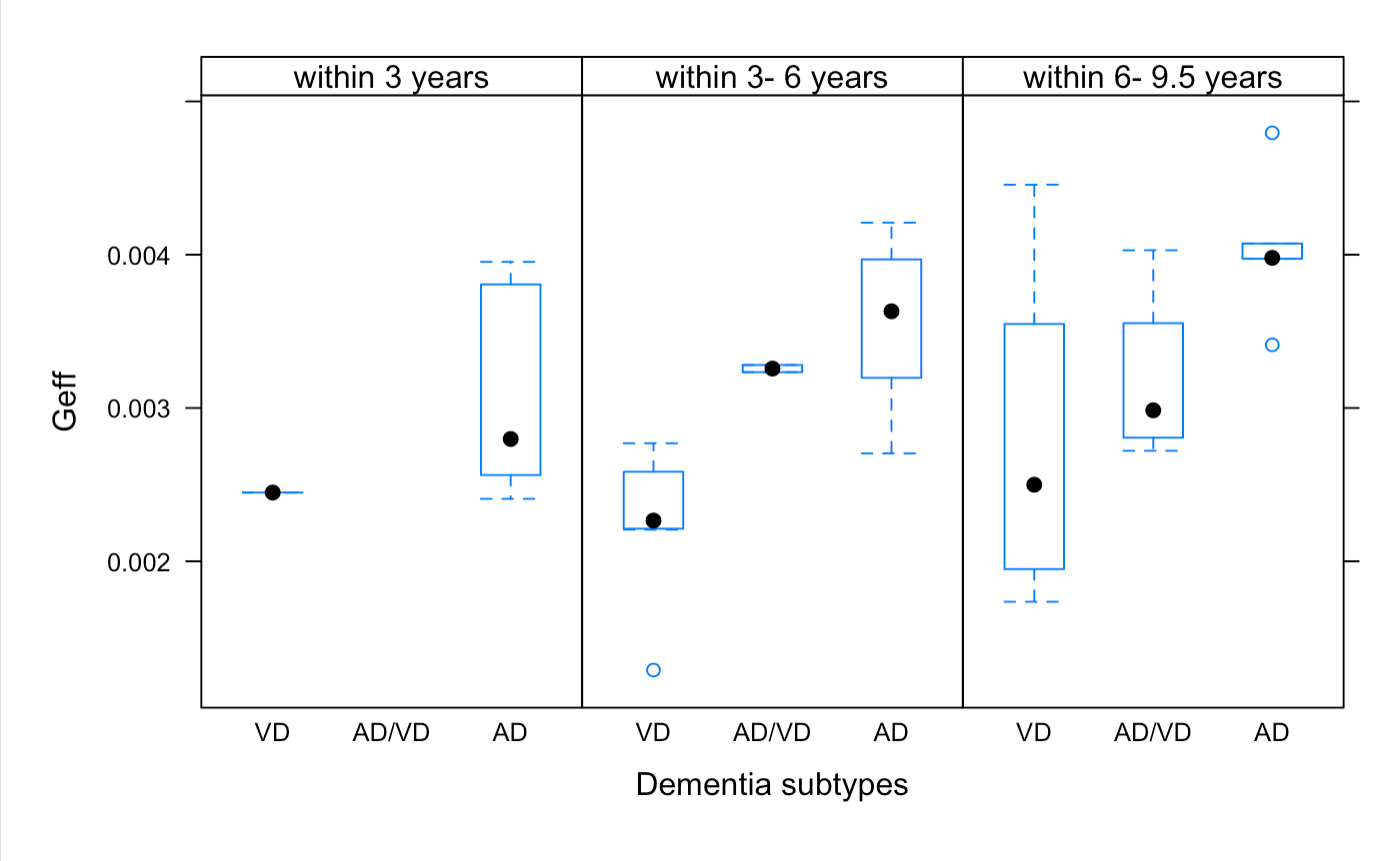**  (E) | Panel A-E show the baseline DTI measures for each of the dementia subtypes across the 3-years time intervals.  AD= Alzheimer’s disease, VD= Vascular Dementia, AD/VD= Mixed dementia with Alzheimer’s and vascular dementia |

**eTable 7: Change in the WM DTI histogram measures in PRESERVE**

| **DTI all WM marker** | **Mean baseline value (SD)** | **Mean 2 year change (SD)** | **P-Value** |
| --- | --- | --- | --- |
| **MD pkval** | 7.64e-04  (4.12e-05) | 3.70e-06  (2.31e-05) | 0.15 |
| **MD PH** | 1.33e-02  (2.45e-03) | -8.29e-04  (1.22e-03) | **3.39e-08** |
| **MD Median** | 7.88e-04  (4.61e-05) | 8.31e-06  (1.70e-05) | **3.35e-05** |
| **MD kurtosis** | 10.00  (4.83) | -1.41  (1.76) | **3.04e-10** |
| **MD skew** | 2.37  (0.58) | -0.23  (0.28) | **3.67e-10** |
| **FA pkval** | 0.31  (4.84e-02) | -3.49e-03  (3.97e-02) | **4.31e-01** |
| **FA PH** | 3.22e-03  (2.38e-04) | -3.48e-05  (1.77e-04) | **8.03e-02** |
| **FA Median** | 3.34e-01  (2.81e-02) | -3.46e-03  (1.29e-02) | **1.83e-02** |
| **FA kurtosis** | 0.52  (0.34) | 4.83e-02  (0.18) | **1.51e-02** |
| **FA skew** | 0.67  (0.15) | 2.27e-02  (6.93e-02) | **4.22e-03** |
| **AxD pkval** | 1.03e-03  (5.51e-05) | 1.40e-05  (5.14e-05) | **0.02** |
| **AxD PH** | 7.50e-03  (8.46e-04) | -2.78e-04  (5.00e-04) | **3.7e-06** |
| **AxD Median** | 1.10e-03  (5.17e-05) | 1.11e-05  (2.31e-05) | **4.8e-05** |
| **AxD kurtosis** | 3.73  (1.43) | -1.66  (0.80) | **< 2e-16** |
| **AxD skew** | 1.38  (0.26) | -0.36  (0.19) | **< 2e-16** |
| **RD pkval** | 6.12e-04  (4.62e-05) | 4.70e-06  (2.80e-05) | 0.14 |
| **RD PH** | 1.18e-02  (1.60e-03) | -4.94e-04  (8.03e-04) | **4.52e-07** |
| **RD Median** | 6.33e-04  (4.70e-05) | 7.90e-06  (1.69e-05) | **7.74e-05** |
| **RD kurtosis** | 8.50  (3.56) | -0.28  (1.41) | **8.26e-02** |
| **RD skew** | 2.08  (0.43) | -3.87e-02  (0.23) | 0.14 |

MD= mean diffusivity, FA= fractional anisotropy, AxD= axial diffusivity, RD= radial diffusivity, pkval= peak value, PH= normalized peak height, P-Value= statistical value of significance with p < 0.05

**eTable 8a Change in WM DTI measures over time in SCANS**

| **DTI all WM marker** | **Estimated mean baseline value (SE)** | **Estimated mean annual change (SE)** | **Wald-test** | **P-Value** |
| --- | --- | --- | --- | --- |
| **MD pkval** | 7.70e-04 (3.18e-06) | 2.94e-06 (6.50e-07) | 4.52 | **1.39e-05** |
| **MD PH** | 1.52e-02 (2.84e-04) | -3.84e-04 (3.28e-05) | -11.70 | **< 0.001** |
| **MD Median** | 7.98e-04 (4.07e-06) | 5.43e-06 (6.06e-07) | 8.96 | **< 0.001** |
| **MD kurtosis** | 15.22  (0.66) | -0.32  (0.13) | -2.36 | **0.02** |
| **MD skew** | 2.75  (0.06) | 0.02  (0.01) | 1.44 | 0.15 |
| **FA pkval** | 0.27  (0.01) | -6.18e-03 (1.80e-03) | -3.43 | **< 0.001** |
| **FA PH** | 3.05e-03 (2.17e-05) | 1.32e-06 (5.42e-06) | 0.24 | 0.807 |
| **FA Median** | 0.29  (2.86e-03) | -2.16e-03 (4.41e-04) | -4.90 | **< 0.001** |
| **FA kurtosis** | 0.52  (0.03) | 0.02  (6.62e-03) | 2.23 | **0.03** |
| **FA skew** | 0.68  (0.01) | 0.01  (2.39e-03) | 4.54 | **< 0.001** |
| **AxD pkval** | 1.01e-03 (3.62e-06) | 3.71e-06 (1.16e-06) | 3.19 | **0.002** |
| **AxD PH** | 8.44e-03 (8.90e-05) | -1.28e-04 (1.20e-05) | -10.63 | **< 0.001** |
| **AxD kurtosis** | 5.20  (0.15) | 0.25  (0.04) | 6.56 | **< 0.001** |
| **AxD skew** | 1.51  (2.43e-02) | 6.32e-02 (7.05e-03) | 8.96 | **< 0.001** |
| **RD pkval** | 6.42e-04 (3.84e-06) | 4.67e-06 (1.39e-06) | 3.37 | **0.001** |
| **RD PH** | 1.26e-02 (1.81e-04) | -2.77e-04 (2.44e-05) | -11.34 | **< 0.001** |
| **RD Median** | 6.65e-04 (4.57e-06) | 6.30e-06 (1.31e-06) | 4.80 | **< 0.001** |
| **RD kurtosis** | 12.06  (0.46) | -0.11  (0.11) | -1.10 | 0.27 |
| **RD skew** | 2.37  (0.04) | 0.04  (0.01) | 3.70 | **< 0.001** |

MD= mean diffusivity, FA= fractional anisotropy, AxD= axial diffusivity, RD= radial diffusivity, pkval= peak value, PH= normalized peak height, P-Value= statistical value of significance with p < 0.05

**eTable 8b Change in WM DTI histogram measures over time in RUN DMC**

| **DTI all WM histogram marker** | **Mean baseline value (SD)** | **Mean 5 year change (SD)** | **Paired t-test** | **P-Value** |
| --- | --- | --- | --- | --- |
| **MD pkval** | 7.81e-04 (3.43e-05) | -2.12e-06 (2.55e-05) | 1.37 | 0.17 |
| **MD PH** | 1.36e-02 (2.20e-03) | -1.49e-04 (1.20e-03) | 2.04 | **0.04** |
| **MD Median** | 7.98e-04 (3.44e-05) | 3.43e-06 (1.25e-05) | -4.50 | **1.01e-05** |
| **MD kurtosis** | 18.53 (6.39) | 6.72e-03  (4.49) | -0.025 | 0.98 |
| **MD skew** | 3.10  (0.61) | 7.21e-02 (0.43) | -2.78 | **0.01** |
| **FA pkval** | 0.33  (6.70e-02) | -3.10e-03 (7.46e-02) | 0.68 | 0.50 |
| **FA PH** | 3.36e-03 (2.97e-04) | 9.60e-05 (2.76e-04) | -5.72 | **2.89e-08** |
| **FA Median** | 0.34  (2.87e-02) | 1.85e-03 (2.09e-02) | -1.45 | 0.15 |
| **FA kurtosis** | 0.43  (0.34) | 4.84e-02 (0.21) | -3.80 | **1.81e-04** |
| **FA skew** | 0.67  (0.14) | 1.48e-02 (8.60e-02) | -2.82 | **5.17e-03** |
| **AxD pkval** | 1.06e-03 (5.07e-05) | 3.42e-06 (5.04e-05) | -1.12 | 0.27 |
| **AxD PH** | 8.80e-03 (8.80e-04) | 4.13e-05 (8.29e-04) | -0.82 | 0.41 |
| **AxD Median** | 1.12e-03 (3.87e-05) | 7.96e-06 (2.00e-05) | -6.54 | **3.08e-10** |
| **AxD kurtosis** | 9.02  (2.42) | 0.29  (2.06) | -2.30 | **0.02** |
| **AxD skew** | 2.04  (0.33) | 9.25e-02 (0.29) | -5.23 | **3.45e-07** |
| **RD pkval** | 6.29e-04 (4.35e-05) | 3.16e-07 (3.31e-05) | -0.16 | 0.88 |
| **RD PH** | 1.02e-02 (1.16e-03) | 1.40e-05 (8.68e-04) | -0.27 | 0.79 |
| **RD Median** | 6.42e-04 (3.79e-05) | 2.04e-06 (1.57e-05) | -2.14 | **0.03** |
| **RD kurtosis** | 1.28  (3.92) | 0.70  (3.47) | -3.29 | **0.01** |
| **RD skew** | 2.38  (0.41) | 0.14  (0.38) | -6.05 | **4.79e-09** |

MD= mean diffusivity, FA= fractional anisotropy, AxD= axial diffusivity, RD= radial diffusivity, pkval= peak value, PH= normalized peak height, P-Value= statistical value of significance with p < 0.05

**eTable 8c Change in WM DTI histogram measures over time in HARMONISATION**

| **DTI all WM marker** | **Mean baseline value (SD)** | **Mean 2 year change (SD)** | **Paired t-test** | **P-Value** |
| --- | --- | --- | --- | --- |
| **MD pkval** | 7.68e-04 (3.76e-05) | 8.25e-06 (3.02e-05) | -3.08 | **2.58e-03** |
| **MD PH** | 0.012 (2.13e-03) | -6.30e-04 (1.26e-03) | 5.65 | **1.00e-07** |
| **MD Median** | 8.82e-04 (6.08e-05) | 2.24e-05 (3.62e-05) | -6.96 | **1.65e-10** |
| **MD kurtosis** | 8.71  (2.52) | -0.69  (1.33) | 5.80 | **4.97e-08** |
| **MD skew** | 2.94  (0.41) | -0.12  (0.22) | 6.22 | **6.76e-09** |
| **FA pkval** | 0.07  (0.02) | -1.10e-03 (0.02) | 0.75 | 0.45 |
| **FA PH** | 5.32e-03 (6.53e-04) | 1.76e-04 (5.83e-04) | -3.39 | **9.37e-04** |
| **FA Median** | 0.19  (0.02) | -5.33e-03 (0.02) | 3.53 | **5.77e-04** |
| **FA kurtosis** | 0.99  (0.82) | 0.13  (0.63) | -2.36 | **0.02** |
| **FA skew** | 1.28  (0.26) | 0.06  (0.19) | -3.22 | **1.64e-03** |
| **AxD pkval** | 1.03e-03 (5.30e-05) | 1.10e-05 (5.14e-05) | -2.41 | **0.02** |
| **AxD PH** | 8.22e-03 (1.00e-03) | -2.52e-04 (7.83e-04) | 3.63 | **4.08e04** |
| **AxD Median** | 1.13e-03 (5.49e-05) | 2.01e-05 (2.86e-05) | -7.93 | **1.00e-12** |
| **AxD kurtosis** | 3.72  (0.924) | -0.27  (0.60) | 5.13 | **1.09e-06** |
| **AxD skew** | 2.16  (0.22) | -0.07  (0.14) | 5.78 | **5.66e-08** |
| **RD pkval** | 6.49e-04 (4.78e-05) | 1.40e-05 (4.15e-05) | -3.80 | **2.26e-04** |
| **RD PH** | 8.94e-03 (1.38e-03) | -4.05e-04 (8.74e-04) | 5.23 | **7.00e-07** |
| **RD Median** | 7.80e-04 (6.57e-05) | 2.34e-05 (3.99e-05) | -6.62 | **9.50e-10** |
| **RD kurtosis** | 5.09  (1.51) | -0.37  (0.82) | 5.05 | **1.53e-06** |
| **RD skew** | 2.35  (0.31) | -0.09  (0.17) | 5.61 | **1.25e-07** |

MD= mean diffusivity, FA= fractional anisotropy, AxD= axial diffusivity, RD= radial diffusivity, pkval= peak value, PH= normalized peak height, P-Value= statistical value of significance with p < 0.05

**eFigure 7**: Correlation heatmap & Percentage of explained variance by the Principal component in change in WM DTI histogram measures. The percentage of explained variance was higher in HARMONISATION than in SCANS or RUN DMC

| **SCANS** | **RUN DMC** | **HARMONISATION** |
| --- | --- | --- |
| 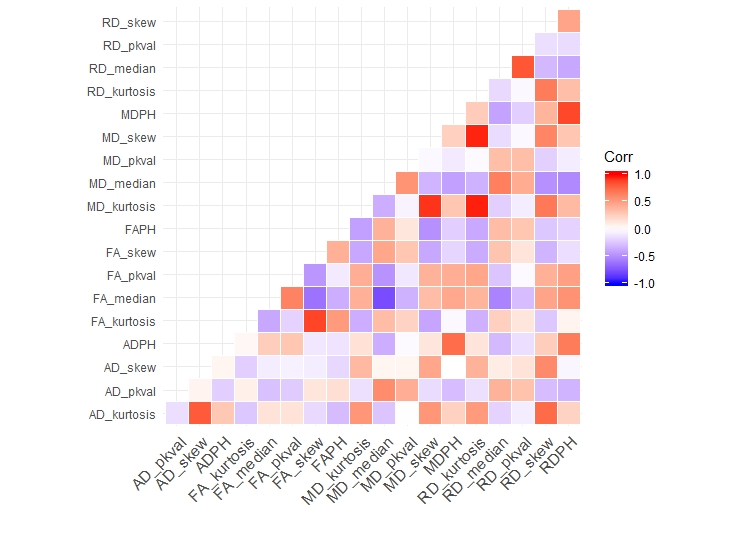 | 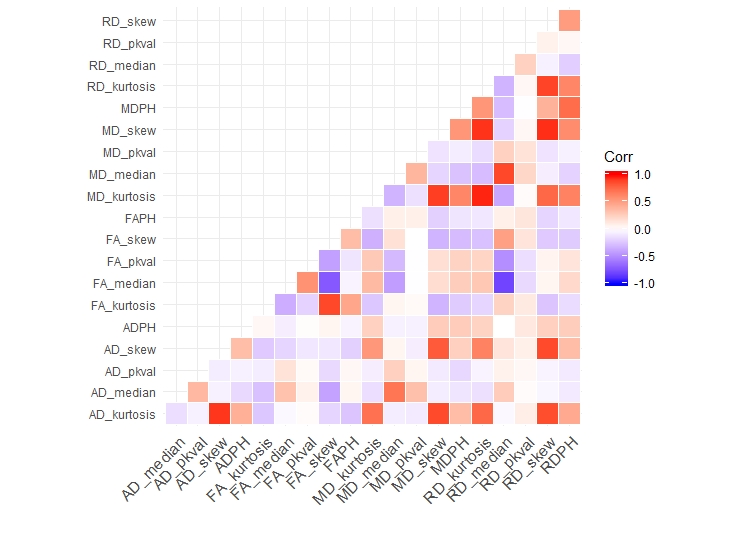 | 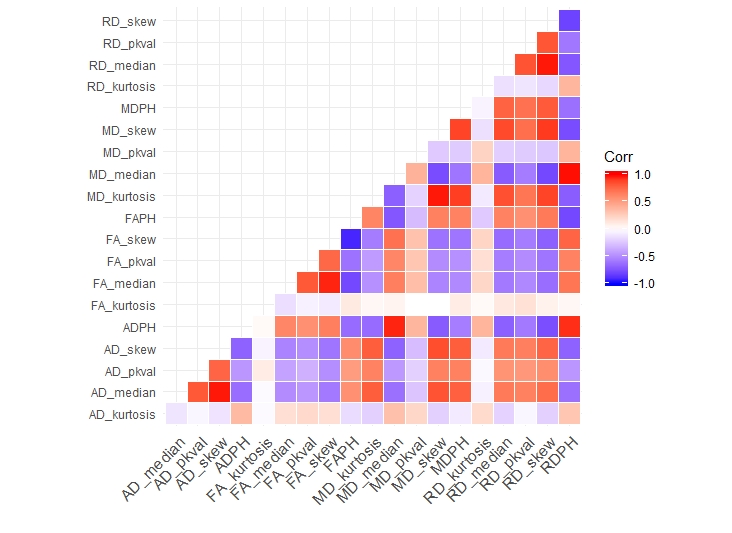 |
| **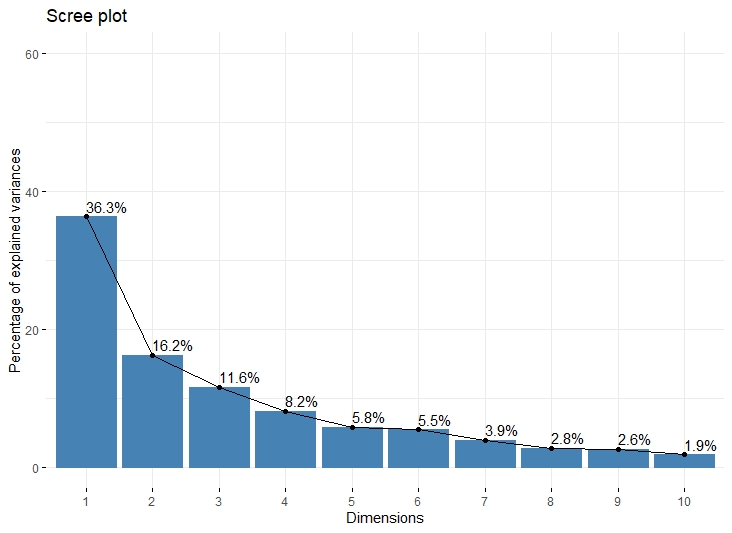** | **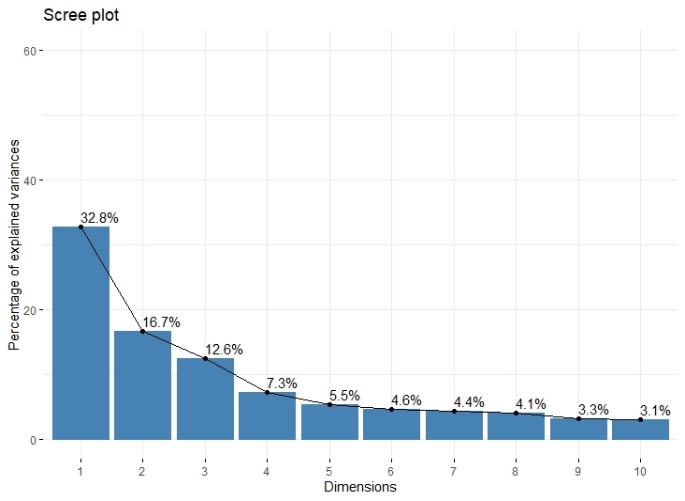** | 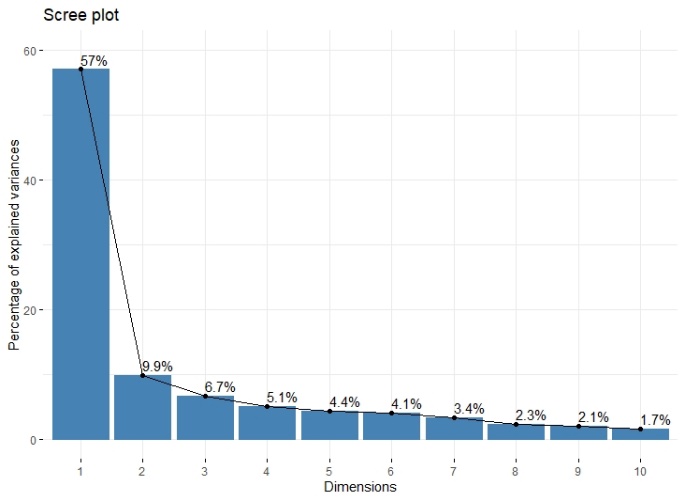 |

Red and blue color on the heatmap refers to a positive and negative association between 2 DTI histogram measures respectively. The strength of the association is illustrated by the marking of the color’s intensity

**eFigure 8. There were no site-related differences for DTI change when controlling for baseline DTI measure in the multicenter study PRESERVE.**

| DTI measure | Differences in mean levels of site with 95% family-wise confidence level |
| --- | --- |
| MD median | 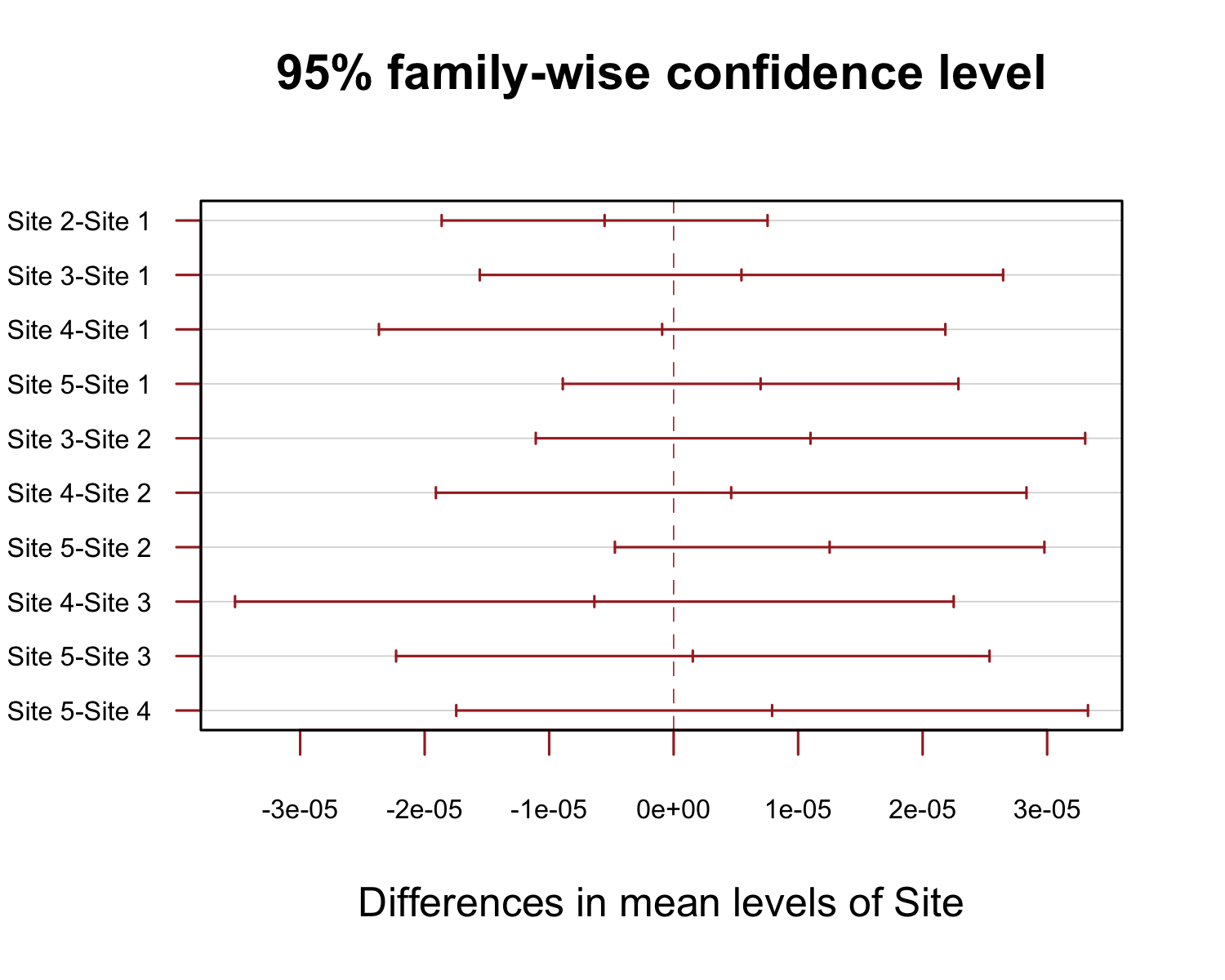 |
| PC1 | 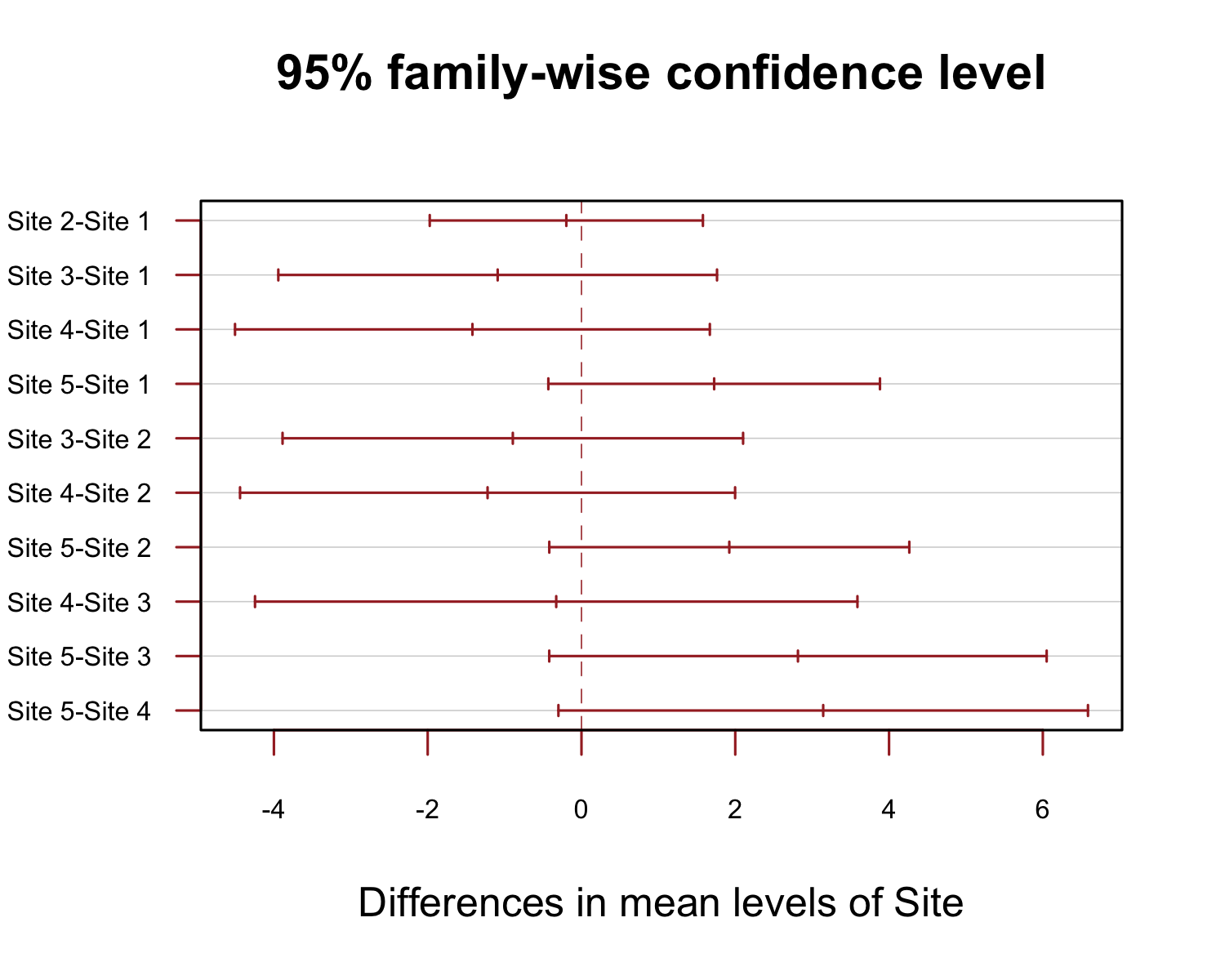 |
| PSMD | 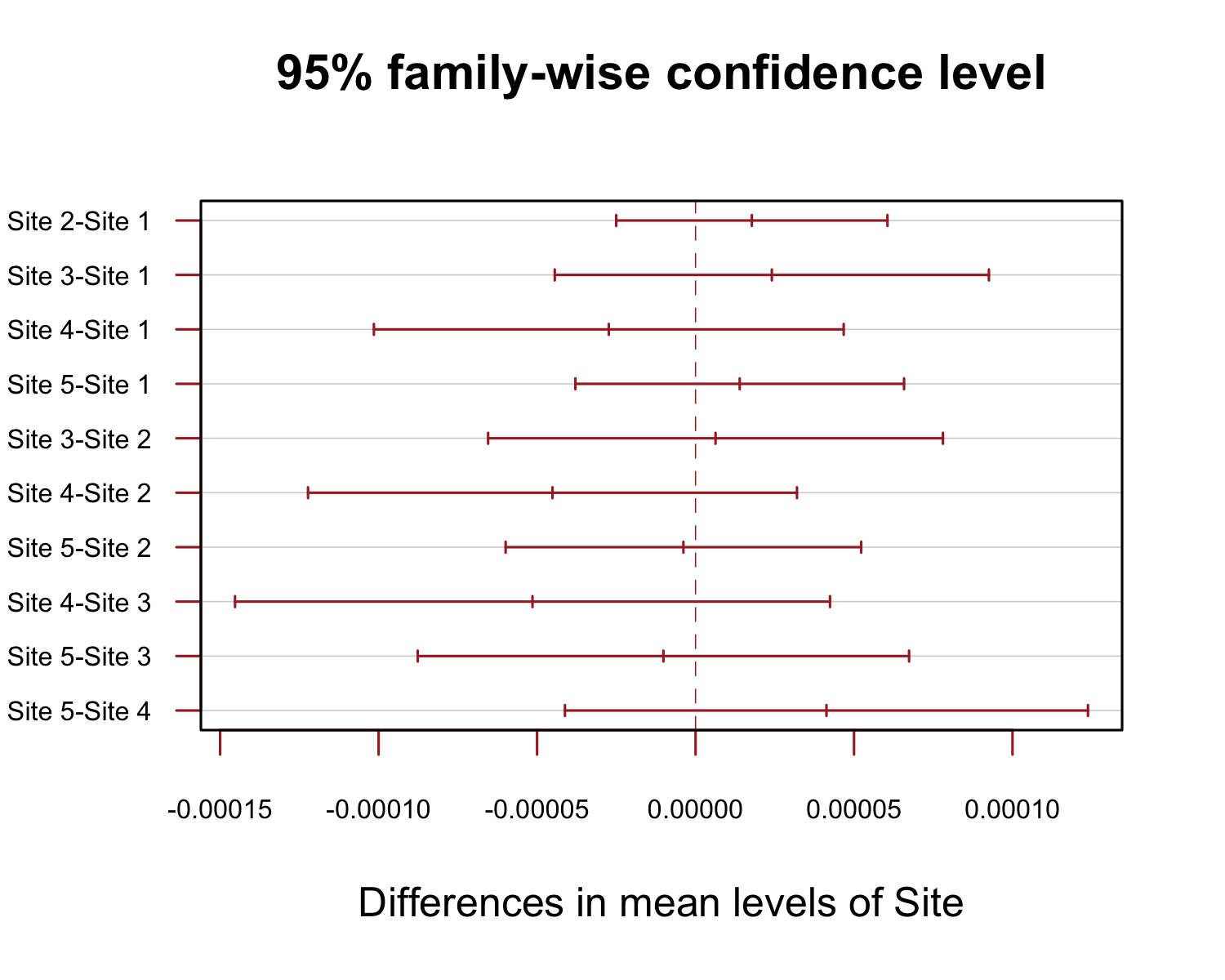 |
| DSEG θ | 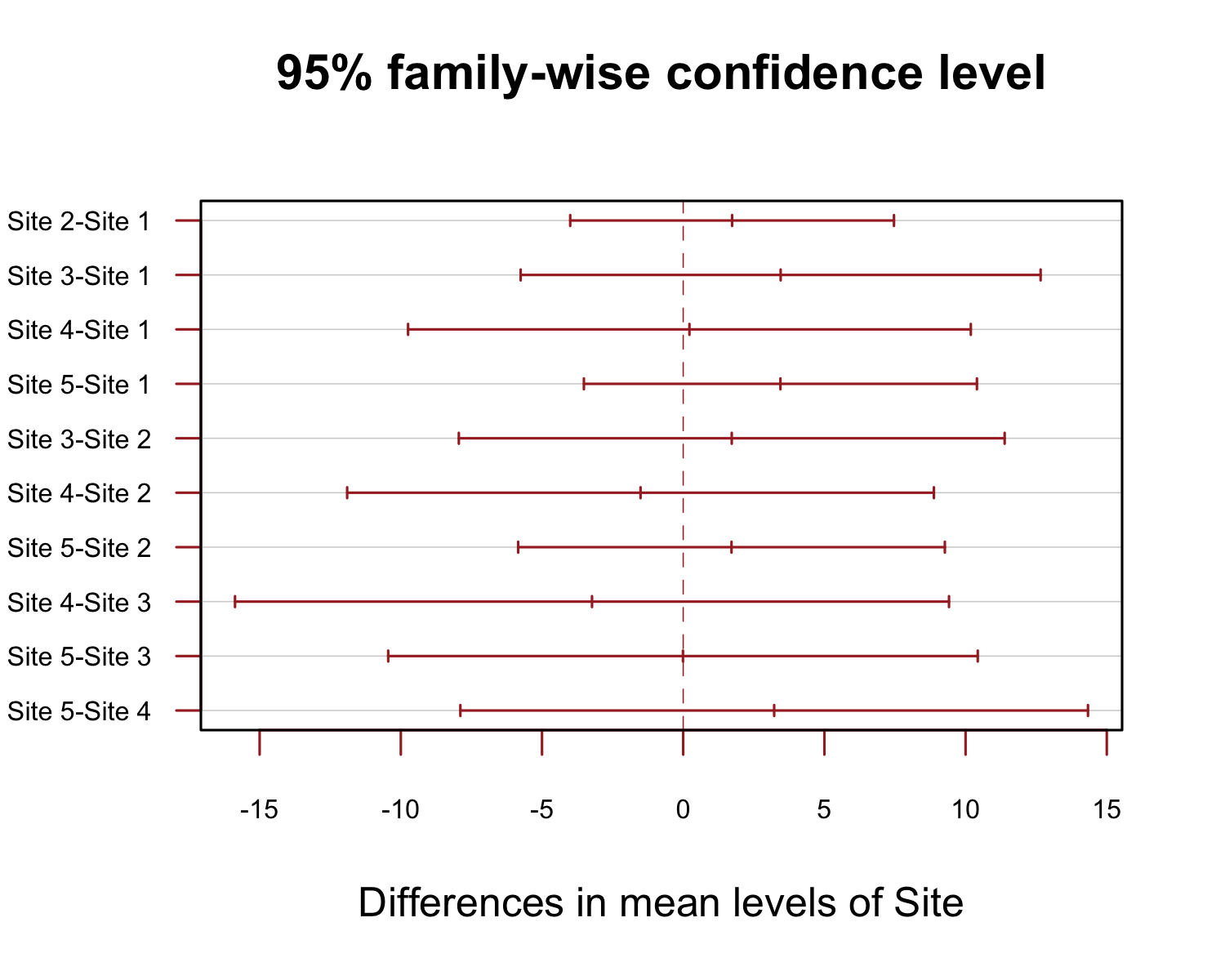 |
| Geff | 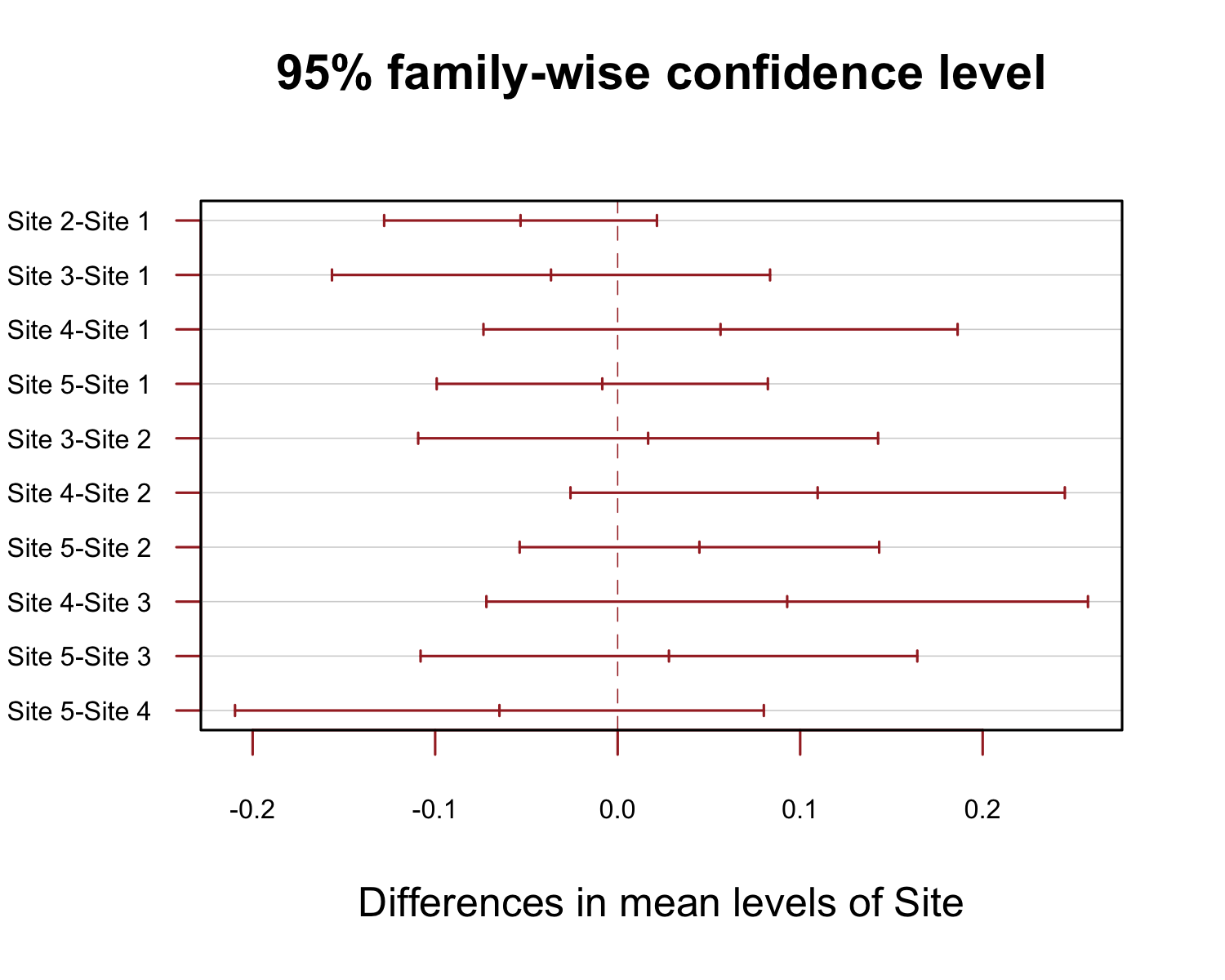 |

eTable 9. Estimated change in DTI markers after removal of imaging measures post dementia diagnosis in SCANS

|  | **Estimated mean baseline value (SE)** | **Estimated mean annual change (SE)** | **Wald-test** | **p-value** |
| --- | --- | --- | --- | --- |
| MD Median | 7.98e-04  (3.92e-06) | 5.37e-06  (5.42e-07) | 9.92 | **< 0.001** |
| PSMD | 3.78e-04  (1.05e-05) | 1.36e-05  (1.89e-06) | 7.21 | **<0.001** |
| DSEG θ | 20.19  (0.84) | 1.17  (0.09) | 13.42 | **<0.001** |
| Geff | 8.12  (0.23) | -0.18  (0.03) | -6.68 | **<0.001** |

MD Median= mean diffusivity median of the all WM histogram, PC1= scores of the first principal component, PSMD= peak width of skeletonized mean diffusivity, DSEG= diffusion tensor image segmentation, Geff= global efficiency network measure, SE= standard error, statistically significant p < 0.05

**eTable 10. Firth's Bias-Reduced Logistic Regression between DTI’s change and dementia conversion in RUN DMC.** There was no significant association between change in DTI and dementia conversion.

|  | **Dementia conversion** | | |
| --- | --- | --- | --- |
|  | *RUN DMC* | | |
| *Change*  *Markers* | *Estimate*  *(95% CI)* | *Chi-square* | *P-Value* |
| MD median | -0.085  (-0.670- 0.444) | 0.094 | 0.760 |
| PC1 | 0.251  (-0.527- 0.899) | 0.416 | 0.519 |
| PSMD | 0.151  (-0.381- 0.569) | 0.390 | 0.532 |
| DSEG θ | 0.381  (-0.273- 1.109) | 1.261 | 0.261 |
| Geff | -0.269  (-0.836- 0.348) | 0.794 | 0.373 |

MD Median= mean diffusivity median of the all WM histogram, PC1= scores of the first principal component, PSMD= peak width of skeletonized mean diffusivity, DSEG= diffusion tensor image segmentation, Geff= global efficiency network measure, β= standardized regression coefficient, 95% CI= 95% confidence interval, statistically significant p < 0.05

**eFigure 9. Change in Mean Skeletonized mean Diffusivity (MSMD) vs. Peak width Skeletonized mean Diffusivity (PSMD) in PRESERVE**. In contrast to PSMD (panel B), there was a significant difference in MSMD (panel A) between baseline and follow-up 2 years.

| 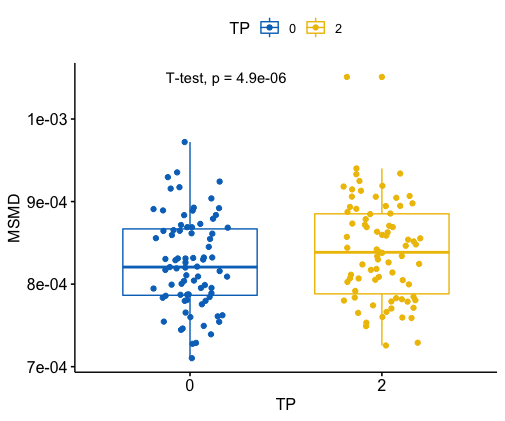A) |
| --- |
| 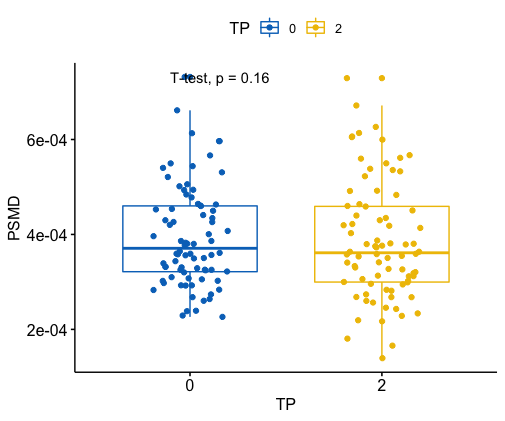B) |

MSMD= Mean skeletonized mean diffusivity, PSMD= Peak width skeletonized mean diffusivity, T-test= paired t-test, TP= time point
